# An explanation of the relationship between mass, metabolic rate and characteristic length for placental mammals

**DOI:** 10.7717/peerj.1228

**Published:** 2015-09-03

**Authors:** Charles C. Frasier

**Keywords:** Morphology, Basal metabolic rate (BMR), Placental mammals, Mitochondria, Froude dynamic similarity, Strouhal dynamic similarity, Body mass, Characteristic length, Sturdiness factor, Phylogenetic groups

## Abstract

The Mass, Metabolism and Length Explanation (MMLE) was advanced in 1984 to explain the relationship between metabolic rate and body mass for birds and mammals. This paper reports on a modernized version of MMLE. MMLE deterministically computes the absolute value of Basal Metabolic Rate (BMR) and body mass for individual animals. MMLE is thus distinct from other examinations of these topics that use species-averaged data to estimate the parameters in a statistically best fit power law relationship such as BMR = a(bodymass)^*b*^. Beginning with the proposition that BMR is proportional to the number of mitochondria in an animal, two primary equations are derived that compute BMR and body mass as functions of an individual animal’s characteristic length and sturdiness factor. The characteristic length is a measureable skeletal length associated with an animal’s means of propulsion. The sturdiness factor expresses how sturdy or gracile an animal is. Eight other parameters occur in the equations that vary little among animals in the same phylogenetic group. The present paper modernizes MMLE by explicitly treating Froude and Strouhal dynamic similarity of mammals’ skeletal musculature, revising the treatment of BMR and using new data to estimate numerical values for the parameters that occur in the equations. A mass and length data set with 575 entries from the orders Rodentia, Chiroptera, Artiodactyla, Carnivora, Perissodactyla and Proboscidea is used. A BMR and mass data set with 436 entries from the orders Rodentia, Chiroptera, Artiodactyla and Carnivora is also used. With the estimated parameter values MMLE can calculate characteristic length and sturdiness factor values so that every BMR and mass datum from the BMR and mass data set can be computed exactly. Furthermore MMLE can calculate characteristic length and sturdiness factor values so that every body mass and length datum from the mass and length data set can be computed exactly. Whether or not MMLE can calculate a sturdiness factor value so that an individual animal’s BMR and body mass can be simultaneously computed given its characteristic length awaits analysis of a data set that simultaneously reports all three of these items for individual animals. However for many of the addressed MMLE homogeneous groups, MMLE can predict the exponent obtained by regression analysis of the BMR and mass data using the exponent obtained by regression analysis of the mass and length data. This argues that MMLE may be able to accurately simultaneously compute BMR and mass for an individual animal.

## Introduction

Most theoretical treatments of Basal Metabolic Rate (BMR) have focused on the exponent in a relationship of the form BMR = *aW^b^* where *W* is body mass. Two concepts have dominated. One concept is geometric similarity in which the value of the exponent *b* = 2/3. The theoretical explanations for this value of the exponent generally involve a balance between heat production and its loss through the body surface (Sarrus & Rameaux in the 1830s as cited by White & Kearney, 2014; Hulbert & Else, 2004; Clarke, Rothery & Isaac, 2010; Roberts, Lightfoot & Porter, 2010; White, 2010; Roberts, Lightfoot & Porter, 2011; Seymour & White, 2011). In the other concept *b* = 3/4 (Kleiber, 1932; Kleiber, 1961). This has been known as “Kleiber’s Law”. A theoretical explanation for *b* = 3/4 was proposed based on a fractal network for a body’s resource supply system (West, Brown & Enquist, 1997; West, Brown & Enquist, 1999). More recently theoretical explanations involving resource distribution networks for which the value of the exponent b can be as small as 2/3 or as large as 3/4 have been proposed (Banavar et al., 2010). The metabolic-level boundaries hypothesis proposes that the exponent b is proportional to the proportions of influence of volume and surface area boundary constraints and thus has a value between 2/3 and 1.0 (Glazier, 2010).

More microscopically, it has been proposed that an animal’s BMR is proportional to the total number of mitochondria in its tissues (Smith, 1956) or to the sum of the metabolic rates of its constituent cells (Kozlowski, Konarzewski & Gawelczyk, 2003). At an even smaller scale it has been proposed in membrane pacemaker theory (Hulbert & Else, 1999) that BMR is governed by the degree of polyunsaturation of membrane phospholipids or, in the Quantum Metabolism theory (Agutter & Tuszynski, 2011), by molecular-cellular processes.

Recent analyses of BMR, body mass data have argued that there is no single exponent b that applies to a phylogenetic class such as mammals. The relationship is more complicated than BMR = *aW^b^* (Kolokotrones et al., 2010). It has also been argued that there are different exponents for different phylogenetic groups such as mammal orders (White, Blackburn & Seymour, 2009; Capellini, Venditti & Barton, 2010) or different orders among insects, fish, reptiles and birds as well as mammals (Isaac & Carbone, 2010).

The dynamic energy budget (DEB) hypothesis (Sousa et al., 2010; White et al., 2011; Maino et al., 2014) can predict the absolute value of BMR rather than the exponent b in the relationship aW^*b*^.

In 1984 the author of the current paper proposed the Mass, Metabolism and Length Explanation (MMLE) theory that also predicts the absolute value of BMR rather than just the exponent b in the relationship BMR = *aW^b^* (Frasier, 1984). A purpose of developing MMLE theory was to relate BMR to measurable skeletal dimensions with the hope of estimating the BMR of extinct animals. Starting with the Smith (1956) proposal that an animal’s BMR is proportional to the total number of mitochondria in its tissues the theoretical derivation took a course that resulted in equations for predicting the absolute value of body mass as a function of a skeletal length dimension as well as predicting the absolute value of BMR as a function of that skeletal dimension. Used together these equations can predict the absolute value of BMR as a function of body mass. This paper will frequently be referred to as the ‘original paper’ in the present paper.

McNab (1988) questioned what should be the best measure of body size against which to scale various functions such as BMR. He presented good reasons why body mass is more variable than linear dimensions in adult mammals. Nevertheless McNab concluded that he would use body mass for want of a better measure of body size. Mass may be an even less reliable measure of body size among bats in which it may vary by as much as 15% to 50% daily (Iriarte-Diaz et al., 2012).

The simplest relationship between body mass and a skeletal length dimension would be geometric similarity in which body mass would be proportional to the cube of the length or, equivalently, the length would be proportional to the cube root of the mass. However Galileo, as cited in Christiansen (1999) and Biewener (2005), recognized in 1638 that geometric similarity implied that small animals would have to be mechanically overbuilt or large animals would have to operate near the limit of mechanical failure. Elastic similarity was proposed as a theoretical solution to this problem (McMahon, 1973; McMahon, 1975) but its predictions did not agree with data (Alexander et al., 1979; Christiansen, 1999). More recently an explanation based on the need of long bones to resist bending and compressive stress has been proposed (Garcia & da Silva, 2004).

It has been recognized that in a relationship relating a skeletal length, l, to body mass raised to an exponent, *W^x^*, the exponent may be different for large terrestrial mammals compared to small terrestrial mammals (Christiansen, 1999; Biewener, 2005). Campione & Evans (2012) found that the regression between the total circumference of the humerus and femur to body mass exhibits the strongest relationship in that the relationship has the highest coefficient of determination values; and the lowest mean percent prediction error, standard error of the estimate and Akaike Information Criterion values of all bivariate regression models for extant mammals and reptiles.

These theoretical explanations for the relationship between skeletal length dimensions and body mass are based on the stresses the bones and their supporting tissues must accommodate. MMLE theory went in a different direction by examining the rate of energy use in skeletal muscle tissues during activity and the relationship between muscle energy use and energy use by a body’s other tissues in the basal metabolic rate state.

Since the original publication of MMLE the amount of BMR, body mass data has vastly expanded as have the resources with which to analyze it. So the purpose of this paper is to use these resources to revisit MMLE theory and test its ability to predict the absolute values of BMR and body mass for individual animals with the new data. MMLE theory will also be modified as necessary.

The running/walking members of the orders Artiodactyla, Carnivora, Perissodactyla and Proboscidea were addressed in the original paper. The present analysis will add Rodentia and Chiroptera (bats) as well as redo the runners/walkers analysis with new data. Rodentia are a small animal counterbalance to the large runners/walkers and they comprise over 40% of placental mammal species. A comprehensive theory should be able to address flying animals as well as terrestrials and bats comprise another 20% of placental mammal species. Swimming animals should also be addressed but there is too little BMR and body mass data to generate reliable results for the orders Pinnipedia, Cetacea and Sirenia. While not addressing the entirety of the placental mammals, the orders that are addressed do represent over two-thirds of the species.

## Summary of MMLE Theory

Article S2 presents an abbreviated version of MMLE theory as originally formulated (Frasier, 1984) together with some necessary modifications. This section is a summary of Article S2.

Nearly all of the energy flux that is measured as basal metabolic rate (BMR) is generated by mitochondria. Although there are other ways to measure it, BMR is most commonly measured by oxygen consumption (Hulbert & Else, 2004). The oxygen is consumed by processes that pump protons across the mitochondrion inner membrane. Heat is produced when some of the protons leak back across the membrane in a controlled fashion as in brown fat or as an uncontrolled basal leak. Otherwise the protons cause the phosphorylation of adenosine diphosphate (ADP) to adenosine triphosphate (ATP) as they return across the inner membrane (Jastroch et al., 2010). ATP is the fuel that powers animal tissues.

MMLE strives to predict the absolute value of the BMR of an animal rather than the exponent b or the constant a in the relationship *aW^b^*. It calculates BMR by summing the energy allocation to an animal’s tissues. The energy allocated to a tissue type is proportional to the number of mitochondria in the tissue. Thus MMLE tries to count the mitochondria in the tissues that compose an animal and then sum these counts for the entire animal. This approach to calculating an animal’s BMR was proposed by (Smith, 1956).

It is a signature feature of MMLE theory that the vertebrate body is represented as a combination of masses instead of a single mass. There are at least two masses: (1) the skeletal musculature which is governed by dynamic similarity and in which the mitochondria are approximately uniformly distributed; and (2) the non-skeletal musculature in which the mitochondria are concentrated in surfaces that surround material with few mitochondria. The heart, kidneys, liver and brain are the principal non-skeletal muscle tissues.

Being a surface, the non-skeletal muscle surface can be mathematically described as the square of a length multiplied by an appropriate constant. Any length could be used as long as the constant is adjusted to make the relationship exact. For MMLE theory the selected length is one that is related to propulsion dynamics. This selected length is called the ‘characteristic length’, *l*. The proportionality constant includes the ‘sturdiness factor’, *s*. *s* is non-dimensional.

In Article S1 an equation is derived for an animal’s body mass, *W*, in terms of *l* and *s*:

(1) \begin{eqnarray*} W=(s l)^{2}{G}_{m}/k f e+((s l)^{2}m{G}_{o}/e)^{1/y}. \end{eqnarray*}

Also an equation is derived for an animal’s BMR in terms of *l* and *s*:

(2) \begin{eqnarray*} B M R={G}_{r}(s l)^{2} \end{eqnarray*}

*G_m_*/*k* is the skeletal muscle mass constant. *G_o_* is the non-skeletal muscle constant. *G_r_* is the resting metabolic rate constant. *G_m_*, *G_o_* and *G_r_* are universal constants that should apply to all vertebrates. *y* is the non-skeletal muscle mass exponent. *m* is a dimensionality factor that adjusts the physical dimensions of this expression to mass. *m* is determined by *y*. *y* and *m* should have the same value for all animals in a phylogenetic group. k is the locomotion constant. k is a function of the type of dynamic similarity that applies to the type of propulsion used by an animal. k should be similar for all vertebrates that are dynamically similar. The fundamental propulsion frequency, *f*, should be the same function of the characteristic length, *l*, for all vertebrates that are dynamically similar. The mitochondrion capability coefficient, *e*, is a constant whose value should be approximately identical for all vertebrates in the same phylogenetic group with the same body temperature. The characteristic length, *l*, and the sturdiness factor, *s*, have unique values for each individual animal.

*G_o_* is defined so that m is dimensionless with a value of 1.0 for geometrically similar non-skeletal musculature for which *y* = 2/3. *G_m_* and k were defined so that k is non-dimensional with a value of 1.0 for running/walking placental mammals.

After analyzing data for running/walking mammals, rodents and bats a general formulation for the fundamental propulsion frequency appears to be *f* = *c*/*l^r^* where c is the propulsion frequency proportionality constant and *r* is the propulsion frequency exponent with a value between 0.5 and 1.0 for species in these mammal orders. Substituting this expression for *f* in Eq. (1) yields:

(3) \begin{eqnarray*} W={s}^{2}{l}^{(2+r)}{G}_{m}/k c e+((s l)^{2}m{G}_{o}/e)^{1/y} \end{eqnarray*}

Animals that are dynamically similar have similar values for the exponent, *r*.

When the gravitational force dominates the dynamics of animals’ movement, two animals are dynamically similar when the ratio of gravitational force to inertial force is the same at corresponding stages of their motions. The animals are Froude similar and they have equal Froude numbers, *F*, where *F* = *u*^2^/*gl* and *u* is speed, *l* is the characteristic length and *g* is the acceleration of gravity (Alexander & Jayes, 1983; Alexander, 2005). Running/walking mammals are Froude similar (Alexander et al., 1979; Alexander, 2005; Raichlen, Pontzer & Shapiro, 2013).

Strouhal similarity obtains when inertial forces are proportional to oscillatory forces. Similarity implies equal Strouhal numbers, St, where St = *fl*/*u* and *f* is the frequency, *l* is the characteristic length and u is speed. The Strouhal number governs a series of vortex growth and shedding regimes for foils undergoing pitching and heaving motions thereby describing the tail or wing kinematics of swimming or flying animals (Taylor, Nudds & Thomas, 2003).

Reynolds similarity obtains when inertial forces are proportional to viscous forces. Similarity implies equal Reynolds numbers, *R*, where *R* = *ulρ*/*ν* and *u* is speed, *l* is characteristic length, *ρ* is fluid density and *ν* is fluid viscosity (Alexander, 2005). Bats are the only animals examined in the present paper for which viscous drag, and hence Reynolds similarity, might be important. Strouhal similarity does apply to bats. If both Reynolds and Strouhal similarity simultaneously apply, then by solving for speed, *u*, in the definitions of Reynolds and Strouhal numbers, the frequency, *f*, is seen to be proportional to the inverse of the characteristic length squared, or *f* = *Rν*/St*ρl*^2^. This sort of dependence of the frequency on the characteristic length was not observed. It should be noted, however, that the characteristic length for viscous drag and that for vortex growth and shedding could be different body dimensions.

Two animals are geometrically similar if one can be made identical to the other by multiplying all its linear dimensions by the same factor (Alexander, 2005). Properties of geometric similarity include surface area, *S*, being proportional to the square of the characteristic length, *l*^2^, and simultaneously volume, *V*, being proportional to the cube of the characteristic length, *l*^3^. Since mass, *W*, is proportional to volume, mass is also proportional to *l*^3^. From Eq. (3) geometric similarity of the skeletal musculature means that the fundamental propulsion frequency exponent *r* = 1.0. The fundamental frequency constant, c, in Eq. (3) has the dimension of speed. If the non-skeletal musculature is also geometrically similar with *y* = 2/3, then the entire animal will be geometrically similar.

Froude and Strouhal dynamic similarity are separately compatible with geometric similarity.

If both Froude and Strouhal similarity simultaneously apply then equating speed in the definitions of the Froude and Strouhal numbers results with the frequency, *f*, being proportional to the pendulum frequency, (*g*/*l*)^0.5^. Substituting this expression for f in Eq. (1) shows that mass, *W*, is not proportional to *l*^3^ and thus geometric similarity does not apply. From Eqs. (2) and (3) geometric similarity means that BMR is proportional to body mass raised to the 2/3 power, *W*^2/3^. Simultaneous Froude and Strouhal similarity implies that BMR is proportional to body mass raised to a power greater than 2/3.

Hereafter, when it is stated that geometric similarity applies it also means that either Froude or Strouhal dynamic similarity also applies.

**Figure 1:**
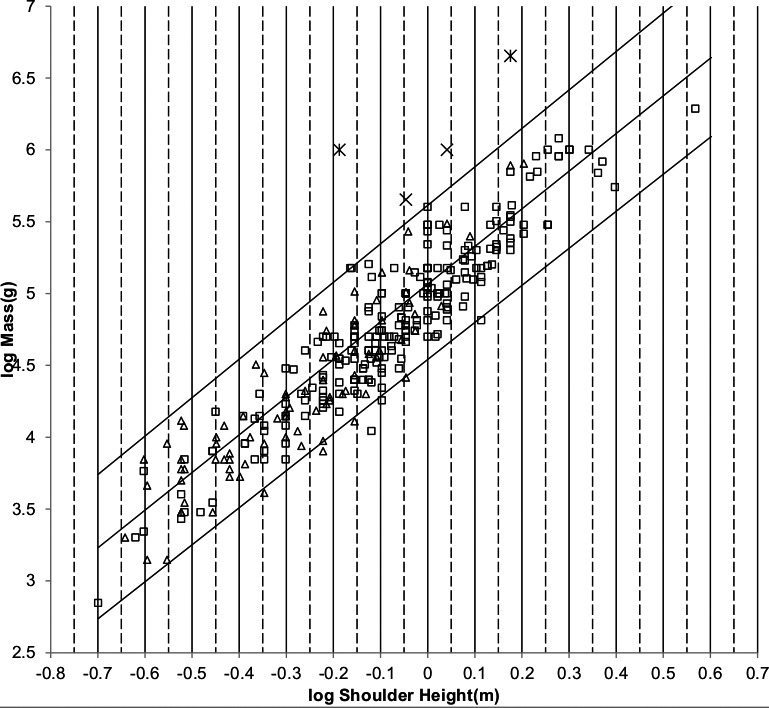
Log body mass as a function of log shoulder height for running/walking Artiodactyla and Carnivora. Data are from Nowak (1999). The upper and lower slanted solid lines are MMLE sturdiness factor boundaries for *y* = 2/3. The upper boundary was generated with a sturdiness factor, *s*, of the square root of 3, (3)^0.5^. The lower boundary was generated with *s* = (3)^−0.5^. The middle slanted line was generated with *s* = 1.0. The slanted lines are for Froude–Strouhal dynamic similarity. The Artiodactyla mass and shoulder height data are marked by open squares. The Carnivora mass and shoulder height data are marked by open triangles. Excluding *Hippopatamus amphibus* marked by crossed Xes and domestic cattle marked by Xes, ${R}_{M}^{2}=0.9997$. The solid vertical lines demark the AVG method first set of cohorts. The dashed vertical lines demark the second set of cohorts.

The sturdiness factor is best understood by looking at Fig. 1. Figure 1 plots 314 samples of log body mass versus log shoulder height for running/walking mammals from the orders Artiocactyla and Carnivora obtained from (Nowak, 1999). Shoulder height is a good surrogate for characteristic length for running/walking mammals. The data in Fig. 1 spread over an area in the two dimensional log shoulder height, log body mass space. It was found in the original paper that most of the area over which the data spreads would be bounded by an upper line computed using Eq. (3) with the sturdiness factor set to the square root of 3, (3)^0.5^, and a lower line computed with the sturdiness factor set to (3)^−0.5^. These boundaries are plotted as the upper and lower slanting lines in Fig. 1. Excluding *Hippopotamus amphibius* and domestic cattle, over 97% of the data plotted in Fig. 1 are contained between these boundary lines.

The data bordering the upper line are for sturdy animals such as a large American black bear (*Ursus americanus*) with *W* = 270 kg, *l* = 0.91 m and *s* = 1.63 or a large water chevrotain (*Hyemoschus aquaticus*) with *W* = 15 kg, *l* = 0.355 m and *s* = 1.35. The data bordering the lower are for gracile animals such as a small bob cat (*Felis rufus*) with *W* = 4.1 kg, *l* = 0.45 m and *s* = 0.556 or a large roe deer (*Capreolus pygargus*) with *W* = 50 kg, *l* = 1.0 m and *s* = 0.687. (Note that posture and limb design do not necessarily reflect the concept of ‘gracile’ as used herein.) At the same characteristic length an animal with a greater sturdiness factor is more massive than an animal with a lesser sturdiness factor—hence the nomenclature ‘sturdiness’ factor.

## Materials and Methods

Surrogate characteristic length and body mass data are available in Nowak (1999). It has been entered into Microsoft Excel spreadsheets that are available as Data S1, Data S2 and Data S3. The author of the present paper is unaware of any substantial collection of BMR and characteristic length data. There is substantial BMR and body mass data in McNab (2008). These data are also available with additional data as a Microsoft Excel spreadsheet in the Supplementary Information for Kolokotrones et al. (2010) at www.nature.com/nature/journal/v464/n7289/suppinfo/nature08920.html which was the source that was used for the present paper. To use this data, Eqs. (1) and (2) can be used together to numerically express BMR as a function of body mass.

For running/walking placental mammals 226 individual mass and shoulder height samples were obtained for Artiodactyla (Data S1). *Hippopotamus amphibius* and *Bos Taurus* (aurochs and domestic cattle) were excluded from the analysis. Aurochs are extinct and domestic cattle have been bred for human purposes rather than survival in the wild. *H. amphibius* is an  amphibious animal well suited to a semi aquatic existence. It is a poor swimmer. It uses a means of locomotion in water that is quite different from that used by other amphibious mammals. Submerged, it runs/walks on the bottom of the body of water through which it is traveling (Nowak, 1999). Buoyancy alters the ratio of gravitational force to inertial force making similarity of *H. amphibius* with more terrestrial runners/walkers suspect.

84 individual mass and shoulder height samples were obtained for Carnivora (Data S1). The Mustelidae were mostly excluded because shoulder height was not given except for wolverines (*Gulo gulo*) and honey badgers (*Mellivora capensis*). Shoulder height was not given for most of the smaller Carnivora so that the smallest sample was a raccoon (*Procyon*) with a mass of 2,000 g and a shoulder height of 0.228m.

26 individual mass and shoulder height samples were obtained for Perissodactyla. Eight individual samples were obtained for Proboscidea (Data S1).

To do Phylogenetically Informed (PI) regression analyses, the individual samples were species-averaged resulting in 129 artiodactyl, 43 carnivoran, 14 perissodactyl and three proboscid species-averaged samples. The BMR and body mass species-averaged data set contained 20 samples for Artiodactyla and 58 samples for Carnivora.

For Rodentia (Nowak, 1999) provides individual data on approximate minimum and maximum masses in grams (g) and head and body lengths in millimeters (mm). The length data are mostly for genera with very little data for species. There is also very little data on shoulder height. 203 individual and 105 taxon-averaged mass, head and body length samples were obtained (Data S2). Kolokotrones et al. (2010) provides 267 species-averaged BMR in watts (*W*) and mass in grams (*g*) samples.

For Chiroptera (bats) (Nowak, 1999) provides data in grams for approximate minimum and maximum body masses. It also provides in millimeters approximate head and body lengths, forearm lengths and tail lengths when a tail is present. The data are mostly for genera with very little data for species. There is little data on wing span. 350 individual and 176 taxon-averaged body mass, head and body length and forearm length samples were obtained (Data S3). 85 species-averaged BMR and body mass samples were obtained. BMR is given in watts and mass is given in grams.

Determining numerical values for the universal constants *G_m_*, *G_o_* and *G_r_* requires three equations. Since Eq. (3) is just a version of Eq. (1) another equation is required. Numerical values for the constants *G_m_* and *G_o_* could be obtained by solving Eq. (3) for two different samples of body mass and characteristic length, l. MMLE uses a more general approach.

An approximation to Eqs. (1) and (3) is:

(4) \begin{eqnarray*} W=d{l}^{x} \end{eqnarray*}

*x* is the approximate mass exponent and d is the approximate mass multiplicative constant. This expression is useful for establishing the approximate relationship between body mass, *W*, and characteristic length, *l*, by regression analysis of *W*, *l* data for a group of animals. For least squares regression a figure of merit for how well the expression represents the data is the coefficient of determination, *R*^2^. The coefficient of determination measures the fraction of the dependent variable’s variance that is explained by the expression.

Least squares regression is performed on the logarithmic version of Eq. (4), log(*W*) = log(*d*) + *x*log(*l*), to obtain best estimate values for log(*d*) and *x*.

Another useful expression is obtained by equating the derivatives with respect to log(*l*) of the logarithms of Eqs. (3) and (4) to obtain, for *s* = 1.0 and geometrically similar non-skeletal musculature where *y* = 2/3, an equation for the exponent *x* in Eq. (4):

(5) \begin{eqnarray*} x=2.0+r+(1-r)({G}_{o}/e)^{3/2}/({l}^{(r-1)}{G}_{m}/k e c+({G}_{o}/e)^{3/2}) \end{eqnarray*}

Eq. (5) is the third equation needed to establish numerical values for the universal constants *G_m_*, *G_o_* and *G_r_*.

The usual way to get the best estimate value for the parameter *x* in equations like Eq. (4) has been by regression analysis of the logarithmic version of the equation. The analyses have used species-averages in which a datum is the average value for a collection of individuals from a single species. In ordinary least squares (OLS) and reduced major axis (RMA) regression analyses it has been assumed that the species-averaged data are statistically independent. Phylogenetically informed (PI) analyses assume that the data are not independent but covary with the degree of phylogenetic relatedness between species. These regression methods are reviewed in detail by (White & Kearney, 2014).

By Eqs. (2) and (3) MMLE deterministically computes the absolute value of BMR and body mass for individual samples rather than a statistical best fit average value for a collection of individuals from a single species. MMLE is thus compatible with data sets that contain individual animal data. It is also compatible with species-averaged data sets by considering each species-averaged datum to correspond with at least one individual member of the species.

If a group of animals have nearly identical values for all the parameters occurring in Eqs. (2) and (3) except for characteristic length, *l*, and sturdiness factor, s, then BMR and body mass can be regressed on characteristic length with sturdiness factor boundaries as illustrated in Fig. 1. As long as the only parameters in Eq. (2) and (3) that distinguish one individual animal from another are characteristic length and sturdiness factor the individuals included in the analysis may be from different species, genera, families, and even orders. Such a group of animals is called a MMLE homogeneous group herein.

The AVG regression method exploits this feature of MMLE to use individual animal data such as the unmodified data from Nowak (1999). The AVG regression method is unique to MMLE. A detailed explanation of the method is given in Article S1.

The appendix to the original paper shows that the OLS regression relationship between body mass and characteristic length is given by the equivalent of Eq. (3) evaluated with a sturdiness factor value of *s* = 1.0 for a MMLE homogeneous group. The *s* = 1.0 regression relationship is plotted by the middle slanting line in Fig. 1. For any particular characteristic length, the mean of the logs of all body masses with that characteristic length that are uniformly distributed between the upper and lower sturdiness factor boundaries is the log of the body mass computed by Eq. (3) evaluated with a sturdiness factor of *s* = 1.0. Using the AVG regression method, MMLE takes advantage of this property to get linear regression relationships between log body mass regressed on log characteristic length with coefficients of determination, *R*^2^, that are very nearly 1.0.

The next section describes using the AVG regression method on characteristic length, body mass data for running/walking placental mammals in the orders Artiodactyla and Carnivora from Nowak (1999) to estimate numerical values for the parameter *x* in Eqs. (4) and (5) and then the parameters *G_m_*/*k* and *G_o_* in Eqs. (1) and (3). The numerical values for *G_m_*/*k* and *G_o_* apply to all placental mammals.

BMR and characteristic length data to estimate a numerical value for the parameter *G_r_* in Eq. (2) was unavailable. Species-averaged BMR and body mass data was available from Kolokotrones et al. (2010).

By Eqs. (2) and (4),

(6) \begin{eqnarray*} \mathrm{BMR}={G}_{r}(W/d)^{2/x}. \end{eqnarray*}

Since *d* and *x* are estimated by regression analysis this expression is reliable only if the coefficient of determination, *R*^2^, is nearly unity for the analysis that estimated *d* and *x*. In the next section it is shown that *R*^2^ = 0.9932 for the AVG regression analysis that estimated *d* and *x* for running/walking placental mammals in the orders Artiodactyla and Carnivora. Although there are complications that will be discussed in the next section a reasonable estimate for the numerical value of *G_r_* is obtained.

**Table 1:** Results of regression analyses for running/walking placental mammals. The regression expressions are: Log(dependent variable) = slope × log(independent variable) + intercept. AVG means the cohort averaging regression method. PI(*n*) means the phylogenetic informed regression method using BayesTraits and the number in parentheses is the estimated value of lambda.

Order or family	Regression type	Independent variable	Dependent variable	Slope	Intercept	*R* ^2^	Number samples
Artiodactyla + Carnivora	AVG	Height (m)	Mass (g)	2.6112	5.0584	0.9932	NA
	PI(0.89	Height(m)	Mass (g)	2.4893	5.076	0.8435	172
All	AVG	Height (m)	Mass (g)	2.8711	5.1677	0.9886	NA
	PI(0.92)	Height (m)	Mass (g)	2.4875	5.2517	0.8424	189
Ruminant Artiodactyla	PI(0.0)	Mass (g)	BMR (watts)	0.7805	−1.8058	0.9742	19
Carnivora	PI(0.87)	Mass (g)	BMR (watts)	0.7579	−1.8826	0.909	59
Carnivora less Mustelidae	PI(0.61)	Mass(g)	BMR (watts)	0.758	−1.8937	0.9053	46
Mustelidae less Enhydra	PI(1.0)	Mass (g)	BMR (watts)	0.6852	−1.4688	0.9653	12
Perissodactyla	PI(1.0)	Height (m)	Mass (g)	1.784	5.4961	0.8046	14

**Notes.**

“All” in the Order or Family column means the combination of Artiodactyla, Carnivora, Perissodactyla and Proboscidea. Height (m) is shoulder height in meters. Mass (g) is body mass in grams. BMR (watts) is basal metabolic rate in watts. NA means Not Applicable.

To solve for a numerical value of *G_r_* an allometric expression of the form BMR = *aW^b^* is obtained by regression analysis. If Eq. (6) is reliable then *b* = 2/*x* and *a* = *G_r_*/*d*^2/*x*^.

Phylogenetically Informed (PI) Generalized Least Squares regression was used to obtain the needed BMR = *aW^b^* expression. The PI Generalized Least Squares regression analyses were conducted using the BayesTraits computer program (Pagel, Meade & Barker, 2004). PI methods are used to control for an assumed lack of statistical independence among species (Freckleton, Harvey & Pagel, 2002; White & Kearney, 2014).

The Microsoft Windows version of BayesTraits together with the companion programs BayesTrees and BayesTreesConverter available at www.evolution.rdg.ac.uk were used. For Rodentia and bats the all mammals phylogenetic tree that is available as supplemental information from the online version of Fritz, Bininda-Emonds & Purvis (2009) at http://onlinelibrary.wiley.com/doi/10.111/j.1461-0248.2009.01307.x.suppinfo was used. For running/walking mammals the updated tree for Carnivora that is available from the online version of Nyakatura & Bininda-Emonds (2013) at www.ncbi.nlm.nih.gov/pmc/articles/PMC3307490/ was grafted onto the all mammals tree. The analyses were performed with the continuous regression model and maximum likelihood analysis type. The inputs were set to estimate the parameter lambda (see Article S1).

Lambda is found by maximum likelihood. It usually varies between 0.0 and 1.0 indicating increasing phenotypic similarity with increasing phylogenetic relatedness. It is a multiplier of the off-diagonal elements of the Generalized Least Squares variance–covariance matrix. Lambda = 0.0 indicates evolution of traits that is independent of phylogeny, while lambda = 1.0 indicates that traits are evolving according to Brownian motion. Intermediate values indicate that traits have evolved according to a process in which the effect of phylogeny is weaker than in the Brownian model (Pagel, 1999; Freckleton, Harvey & Pagel, 2002; Pagel, Meade & Barker, 2004; Capellini, Venditti & Barton, 2010). BayesTraits results generated with lambda = 0.0 are the same as those obtained with Ordinary Least Squares (OLS) linear regression. Results generated with lambda = 1.0 are the same as those generated by phylogenetically independent contrasts (Capellini, Venditti & Barton, 2010). Occasionally lambda was estimated to be greater than 1.0. This can be interpreted as traits that are more similar than what is predicted by Brownian motion (Freckleton, Harvey & Pagel, 2002).

Since BayesTraits estimates maximum likelihood for a hypothesis (such as the applicable value of lambda), the log likelihood ratio for two hypotheses can be computed. By convention a value of 4.0 or greater for the ratio is taken as evidence that one of the hypotheses explains the data significantly better than the other (Pagel, 1999).

PI regression analyses were performed for both BMR regressed on body mass and body mass regressed on characteristic length. The species-averaged BMR, body mass data from Kolokotrones et al. (2010) was inputted directly to BayesTraits. The body mass, characteristic length data from Nowak (1999) are mostly maximum and minimum measurements of individuals from the same species or genera in which a datum is the measurements for the largest or smallest individual measured in the taxon. Body mass, characteristic length data were taxon-averaged before being inputted to BayesTraits.

Together with the dynamic similarity implied by the mode of locomotion for a group of animals, PI regression relationships are helpful for partitioning populations into MMLE homogeneous groups to which the AVG regression technique can be applied as when a geometrically similar partition can be identified by a log(mass) regressed on log(length) slope of 3.0 or a log(BMR) regressed on log(mass) slope of 2/3. Since the only parameters in Eq. (3) that differ between individual members of a MMLE homogeneous group are the characteristic length, *l*, and the sturdiness factor, *s*, further consideration of the relatedness of members should not be necessary to use Eq. (3).

Regression analysis measures the error for a datum as the distance between the datum and a line established by the regression analysis. The line minimizes the sum of the squares of the errors.

In MMLE theory the OLS regression line can be replaced by a ‘band’. The band is the area enclosed by the sturdiness factor boundaries. Recognizing this fact, *R*^2^ can be replaced by a MMLE version that is denoted “${R}_{M}^{2}$”. ${R}_{M}^{2}$ is computed in the same ways as *R*^2^ except that the ‘error’ for a datum that falls between the sturdiness factor boundaries is zero and the ‘error’ for a datum that falls outside the sturdiness factor boundaries is the distance between the datum and the nearest sturdiness factor boundary. ${R}_{M}^{2}$ is interpreted as a measure of how well data clusters within the area bounded by the sturdiness factor boundaries. ${R}_{M}^{2}$ is useful for estimating values for the parameters that appear in Eq. (3) when they may differ from the values established for running/walking placental mammals.

Excluding *Hippopotamus amphibius* and domestic cattle, ${R}_{M}^{2}=0.9997$ for the data in Fig. 1. ${R}_{M}^{2}$ is very nearly unity because the data considered in the present paper mostly lie between the sturdiness factor boundaries or very near to a boundary. Coverage, *R*, which is the fraction of the data that lies between the boundaries, is another figure of merit that is usually smaller than ${R}_{M}^{2}\cdot R=0.9774$ for the data in Fig. 1.

By examining individual animal metabolic rates and masses, Hudson, Isaac & Reuman (2013) recently reported substantial metabolic rate heterogeneity at the species level and commented that this is a fact that cannot be revealed by species-averaged data sets. It was further commented that individual data might be more important than species-averaged data in determining the outcome of ecological interactions and hence selection. Heterogeneity is predicted by Eqs. (2) and (3) due to variation of characteristic length and sturdiness factor among the individuals composing a species. The AVG regression method is compatible with species level heterogeneity.

Sturdiness factor boundaries and ${R}_{M}^{2}$ can also apply to the BMR, body mass data from Kolokotrones et al. (2010). The boundaries are calculated by using Eq. (3) with the bounding sturdiness factors to calculate the body mass and using Eq. (2) with the bounding sturdiness factors to calculate the BMR for characteristic lengths that span the range of interest. The associated value of ${R}_{M}^{2}$ can then computed using the BMR as a function of body mass boundaries.

The calculated numerical values reported in the present paper are given with four significant digits to the right of the decimal point. It is suspected that the data used is not accurate enough to support this precision.

## Running/Walking Placental Mammals Results

This is an abbreviated version of the analysis of running/walking placental mammals. The detailed analysis is available in Article S1.

The original paper established the numerical values for the constants in the MMLE equations by AVG analysis of 163 samples of the running/walking members of the orders Artiodactyla, Carnivora, Perrisodactyla and Proboscidea from Walker (1968). The four orders were analyzed together as a MMLE homogeneous group because their mode of locomotion was similar and it was expected that they would be dynamically similar so that the fundamental frequency of propulsion in Eq. (3) would scale similarly. Dynamic similarity rather than genetic similarity was considered more controlling of the relationship between mass and characteristic length in Eq. (3) because these orders are terrestrial runners/walkers that have evolved together in a predator/prey arms race. The genomes among these orders that have survived are the ones that have produced dynamically similar phenotypes suited to survival in terrestrial environments. The Artiodactyla are genetically more similar to the aquatic swimming Cetacea than they are to the other terrestrial running/walking orders (Bininda-Emonds et al., 2007) but their manner of locomotion is dynamically more similar to the other runners/walkers than it is to the swimming Cetacea. The Carnivora are more genetically similar to the aquatic swimming Pinnepedia than they are to the other terrestrial running/walking orders (Nyakatura & Bininda-Emonds, 2013) but their manner of locomotion is also more dynamically similar to the other runners/walkers than it is to the swimming Pinnepedia.

The fundamental frequency of propulsion was established as the pendulum frequency obtained when both Froude and Strouhal dynamic similarity apply simultaneously. Limb length was considered to be the characteristic length but little limb length data was available. Shoulder height data was more available, so shoulder height was adopted as an approximation of the characteristic length, *l*. The results were *x* = 2.66, *G_m_*/*k* = 295,000 g/m^2^ s and *G_o_* = 1,353 g^0.667^/m^2^ in the units used in the present paper.

BMR was predicted to scale as body mass raised to the 2/*x* power. Since *x* = 2.66 this meant that BMR scaled as body mass raised to the 0.75 power in agreement with Kleiber’s law. The numerical values of the parameters in the version of Kleiber’s law obtained by Economos (1982) using (Kleiber, 1961) data with additional BMR, body mass data were used to estimate the constant *G_r_* in Eq. (2) to be 142 watts/m^2^.

An expanded version of the methodology in the original paper was used to determine the constants in Eqs. (1), (2) and (3) in the present paper. AVG and PI regressions of the new body mass on shoulder height data were used to determine the exponent in Eq. (4). This regression relationship was then to be used to determined body mass at a ‘middle’ shoulder height. Then Eqs. (3) and (5) were solved simultaneously for the constants *G_m_*/*k* and *G_o_* occurring in the equations using unity sturdiness factor and unity mitochondrion capability quotient. The method for determining the constant *G_r_* in Eq. (2) was more complicated.

Although Raichlen, Pontzer & Shapiro (2013) find that hip joint to limb center of mass is a better length for establishing the pendulum frequency, shoulder height was again used as an approximation of the characteristic length due to its greater availability.

Table 1 shows the AVG and PI regression analysis results obtained with the running/walking mammal data. For mass regressed on shoulder height, there were too few samples of Proboscidea to perform a meaningful PI regression analysis and the AVG first and second cohort sets did not converge. The PI slope and intercept for Perissodactyla differed significantly from those for Carnivora and Artiodactyla and the AVG first and second cohort sets did not converge. However the slope and intercept for Carnivora and Artiodactyla were not significantly different. They were also not significantly different from the slope and intercept obtained from PI regression analysis of the combination of Carnivora and Artiodactyla and the AVG first and second cohort sets did converge. This strengthened the conjecture that Carnivora and Artiodactyla were dynamically similar with a fundamental propulsion frequency in Eq. (3) that scales similarly with characteristic length. For these reasons Carnivora and Artiodactyla were considered to be a Froude–Strouhal MMLE homogeneous group. They were analyzed together. Proboscidea and Prissodactyla were considered separately.

From the Artiodactyla + Carnivora AVG mass on shoulder height regression slope the exponent, *x*, for Eq. (4) is 2.61. Since total body mass scales with an exponent, 2.61, that is greater than the exponent with which the skeletal muscle mass scales, 2.5, the non-skeletal muscle mass must scale with an exponent greater than 2.61. The simplest assumption is geometric similarity so that the non-skeletal muscle mass scales with an exponent of 3.0. The corresponding value for *y* is 2/3. Simultaneously solving Eqs. (4) and (5) results in *G_m_*/*k* = 274,000 g/m^2^ s and *G_o_* = 900 g^0.667^/m^2^.

The PI mass on shoulder height regression slope for Artiodactyla + Carnivora is not significantly different from 2.5 as the log likelihood ratio for the Table 1 slope and 2.5 is less than 4.0. Since for Froude–Strouhal similarity the skeletal musculature scales with an exponent of 2.5, this implies that the non-skeletal musculature also scales with an exponent of 2.5 which means that y has the non-geometric value of *y* = 0.8 in Eq. (3). The equivalent of Eq. (5) with *y* = 0.8 and *r* = 0.5 only states that *x* = 2.5 and provides no information for estimating *G_m_*/*k* and *G_o_*. Additionally Eq. (3) must be used to estimate the dimensionality factor, *m*.

*G_m_*/*k* should not change if *y* changes. Using the previously established values of *G_m_*/*k* and *G_o_* with *y* = 0.8 in Eq. (3) results with a value for the dimensionality factor of *m* = 4.425 g^0.133^.

The new values for *G_m_*/*k* and *G_o_* are less than the values computed in the original paper. The 163 samples in the original paper included two proboscideans and 11 perissodactyls whereas the 310 samples in the present paper were entirely of Artiodactyla or Carnivora.

**Figure 2:**
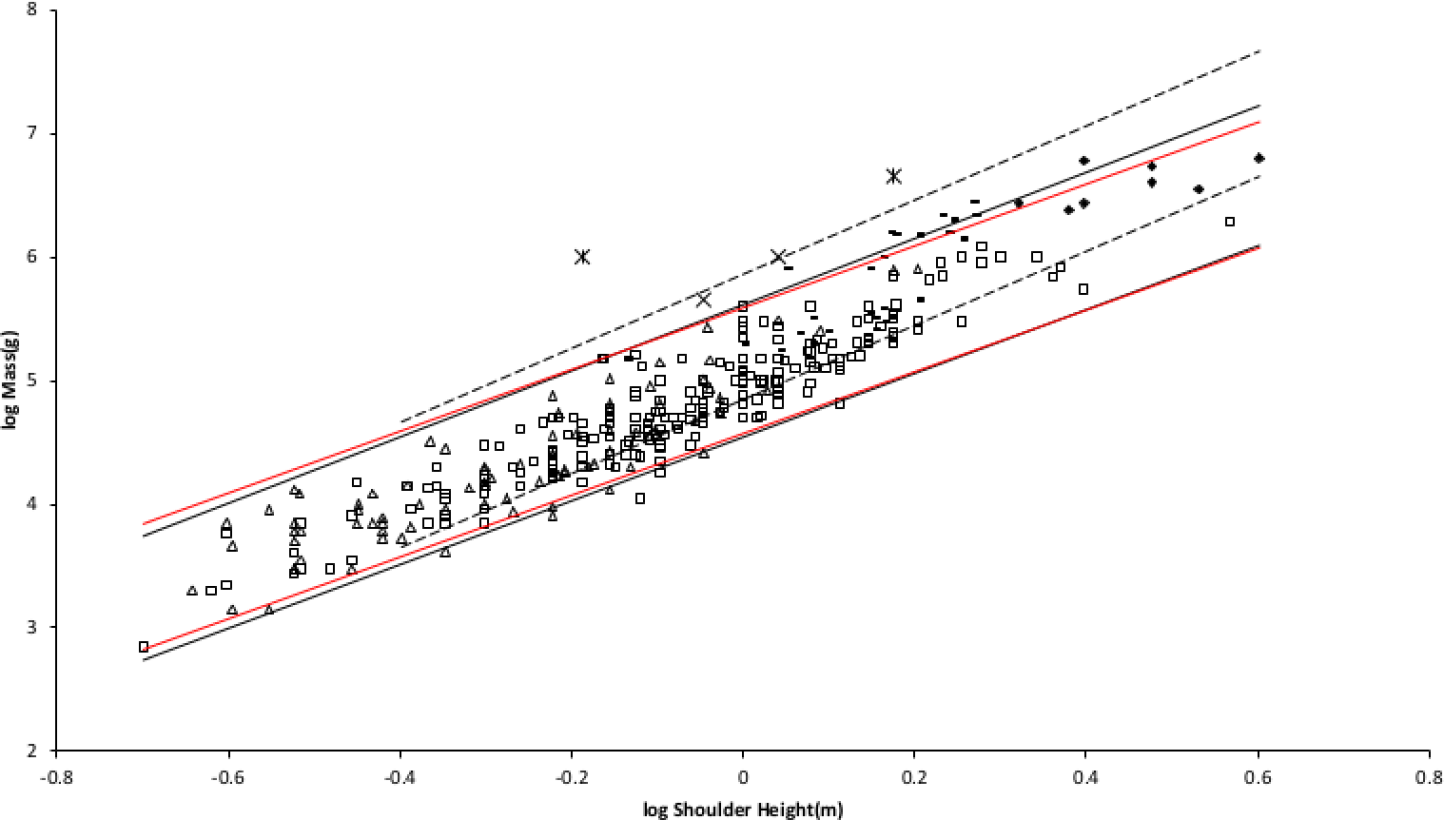
Log body mass as a function of log shoulder height for running/walking placental mammals. Data are from Nowak (1999). The solid and dashed lines are MMLE sturdiness factor boundaries. The upper boundaries were generated with a sturdiness factor *s* = (3)^0.5^. The lower boundaries were generated with *s* = (3)^−0.5^. The solid boundary lines are for Froude–Strouhal dynamic similarity. The black solid lines are for *y* = 2/3. The colored solid lines are for *y* = 0.8. The dashed boundary lines are for geometric similarity. The colored boundary lines, the geometric similarity boundary lines and the Perissodactyla and Proboscidea mass, shoulder height data have been added to the artiodactyl and carnivoran data displayed in Fig. 1. For Artiodactyla and Carnivora ${R}_{M}^{2}=0.9997$ with respect to the solid black boundaries and ${R}_{M}^{2}=0.9992$ with respect to the colored boundaries. For Perissodactyla and Proboscidea ${R}_{M}^{2}=1.0$ with respect to the geometric similarity boundaries. Perissodactyls are marked with solid rectangles. Proboscideans are marked with solid diamonds. Aritiodactyla data are marked with open squares. Carnivora data are marked with open triangles. Crossed Xes mark *Hippopotamus amphibious*. Xes mark domestic cattle.

Figure 2 shows the MMLE mass as a function of shoulder height sturdiness factor boundaries for simultaneous Froude–Strouhal dynamic similarity as computed by Eq. (3) evaluated with the new values for *G_m_*/*k* and *G_o_* for both *y* = 2/3 and for *y* = 0.8. The data spans this full range of sturdiness factor for both values of *y*. The boundaries are hardly distinguishable for the two y values.

The relationship between mass and shoulder height for Perissodactyla and Proboscidea may be better explained by geometric similarity with a fundamental frequency of 1.4 m/s divided by shoulder height (see Article S1).

There are doubts that ruminant artiodactyls can meet the criteria for measuring BMR (McNab, 1997; White & Seymour, 2003). All but one of the artiodactyl samples were ruminants. For these reasons the BMR and mass data for ruminant Artiodactyla and Carnivora were analyzed separately. Ruminant inability to achieve a post absorptive state should not affect the relationship between body mass and shoulder height.

There is a major composition difference between the mass and shoulder height data and the BMR and mass data for Carnivora. Mustelidae compose only about 1% of the mass and shoulder height data whereas they compose over 20% of the BMR and mass data. For this reason the mustelid data was separated from the rest of the Carnivora data as shown in Table 1.

Since the coefficient of determination, *R*^2^, for the AVG Artiodactyla + Carnivora mass on shoulder height regression in Table 1 is very nearly unity, Eq. (6) can be used. With the *G_m_*/*k* and *G_o_* values just determined, *G_r_* = 95.6 W^−0.0079^ watts/m^2^. The mass residual is not significant as the log likelihood ratio for the exponent obtained with Eq. (6) and the exponent in Table 1 is essentially 0.

Using the Carnivora less Mustelidae PI mass regressed on length and the PI BMR regressed on mass relationships in Table 1 results with *G_r_* = 94.5 watts/m^2^.

**Figure 3:**
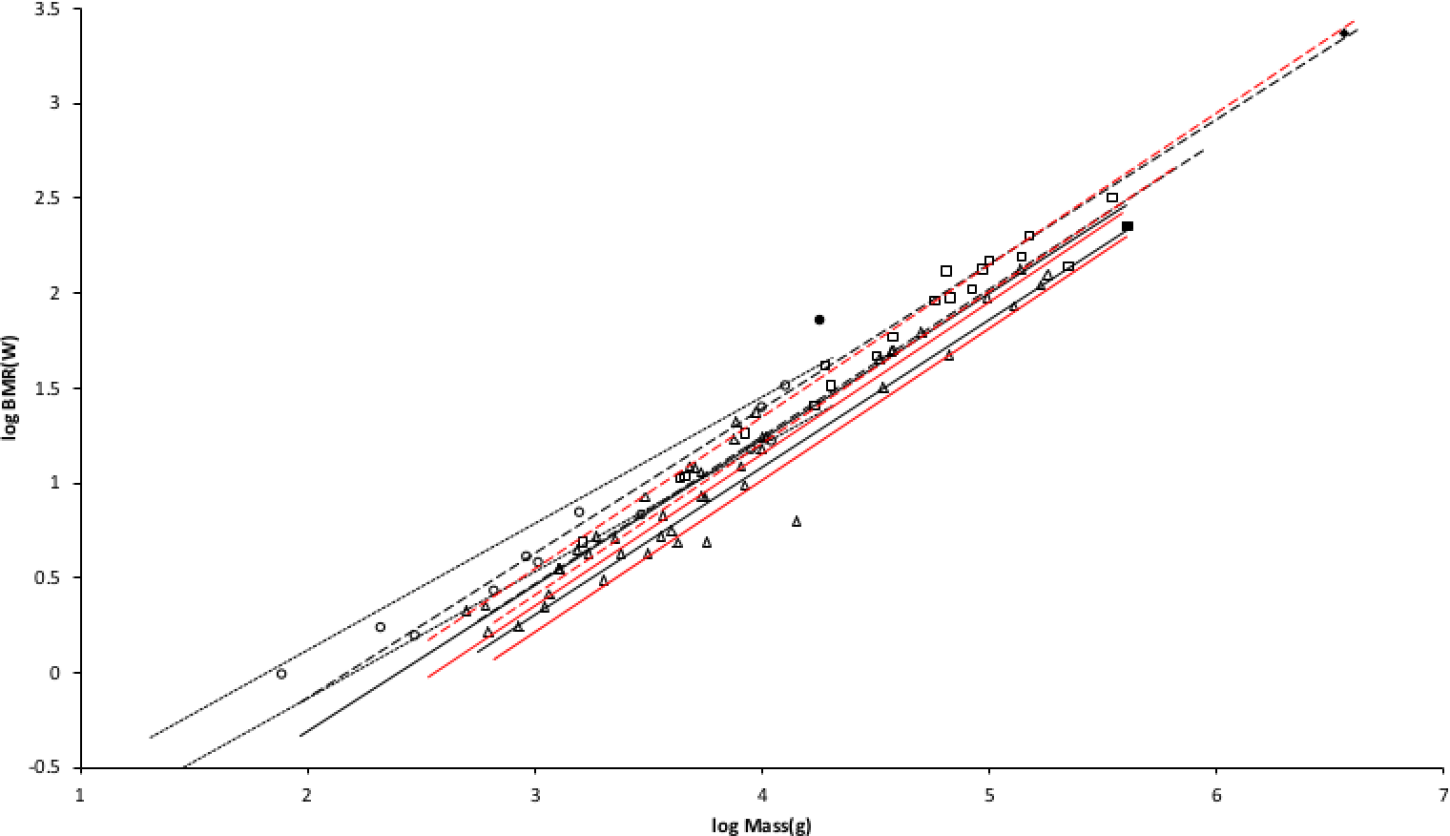
Log BMR as a function of log body mass for running/walking placental mammals. The *Elephas maximus* datum marked by a solid diamond is from Savage et al. (2004). All other data are species-averages from Kolokotrones et al. (2010). The solid, dashed, and dotted lines are MMLE sturdiness factor boundaries. The upper boundaries were generated with a sturdiness factor *s* = (3)^0.5^. The lower boundaries were generated with *s* = (3)^−0.5^. The black lines are for *y* = 2/3. The colored lines are for *y* = 0.8. The steeper sloping boundary lines are for Froude–Strouhal dynamic similarity. The shallower sloping boundary lines are for geometric similarity. Ruminant artiodactyl data are marked by open squares and ${R}_{M}^{2}$ is 0.9921 with respect to the dashed Froude–Strouhal black boundaries and ${R}_{M}^{2}$ is 0.9919 with respect to the dashed colored boundaries. *Camelius dromedarius* is a non-ruminant artiodactyl marked by a solid square. Carnivora less Mustelidae are marked with open triangles and ${R}_{M}^{2}$ is 0.9752 with respect to the solid Froude–Strouhal black boundaries and ${R}_{M}^{2}$ is 0.9655 with respect to the solid colored boundaries. Mustelids except Enhydra are marked with open circles and ${R}_{M}^{2}$ is 0.9999 with respect to the dotted geometric black boundaries. *Enhydra lutris* is an ocean going swimming mustelid marked by a solid circle.

A middle value of *G_r_* = 95 watts/m^2^ was used to generate the MMLE sturdiness factor boundaries in Fig. 3. As discussed in the summary of the derivation of the MMLE equations, this value of *G_r_* should be the basic value for all non-ruminant placental mammals.

Similarly, values for *G_rR_* for ruminant artiodactyls of 138 watts/m^2^ and 144 watts/m^2^ are obtained. *G_rR_* = 138 watts/m^2^ was used to generate the MMLE sturdiness factor boundaries in Fig. 3.

The difference between slopes when regressing BMR on body mass for ruminant artiodactyls and carnivorans excluding mustelids (Table 1) are not significant as the log likelihood ratio is 2.8. However the difference between the intercepts is significant as the log likelihood ratio is greater than 4.0. The significantly different intercepts support separating ruminant Artiodactyla and Carnivora less Mustelidae for BMR analyses.

An increased mitochondrion capability quotient is not the reason that *G_rR_* is greater than *G_r_*. By Eq. (3) a greater mitochondrion capability quotient would result in a less massive animal for the same characteristic length. Figure 2 shows that both Artiodactyla and Carnivora have similar masses at the same characteristic length and their PI mass regressed on characteristic length slopes are not significantly different. The difference between *G_rR_* and *G_r_* is more likely the result of sustained digestive activity by ruminant Artiodactyla (McNab, 1997; White & Seymour, 2003).

Figure 3 shows the MMLE BMR as a function of body mass sturdiness factor boundaries for Froude–Strouhal similarity evaluated with the new values for *G_r_* and *G_rR_*. The difference between the *y* = 2/3 and *y* = 0.8 slopes is significant as the log likelihood ratio is 5.9. In terms of ${R}_{M}^{2}$ the differences are barely distinguishable.

Ruminant artiodactyls do have a BMR that is elevated with respect to Carnivora of the same mass. The single non-ruminant artiodactyl datum, a dromedary camel (*Camelus dromedaries*), is embraced by the non-mustelidae carnivoran MMLE boundaries rather than the ruminant boundaries.

The mustelid data in Fig. 3 is better embraced by the ruminant MMLE sturdiness factor boundaries, but mustelids do not have the digestive features that are the probable source of the ruminants’ elevated BMR. The non-mustelid carnivoran value for *G_r_* should apply to the Mustelidae also, but their MMLE boundaries hardly embrace any of the mustelids.

Separating the ocean going swimming sea otter (*Enhydra lutris*) from the rest of the mustelids leads to a slope of 0.69 as shown in Table 1. This slope is not significantly different from the geometric similarity slope of 0.67 as the log likelihood ratio for these slopes is 0.4. Geometric rather than Froude–Strouhal similarity supports separating Mustelidae from the rest of Carnivora for BMR analyses. Equation (3) with *y* = 2/3 and a geometric similarity fundamental frequency of 1.4 (m/s)/l and Eq. (2) are the MMLE equations for relating BMR to body mass for geometric similarity. Of the constant parameters in these equations, values applicable to Mustelidae for *G_m_*, *G_o_*, and *G_r_* have been established. The MMLE parameters that could be adjusted to account for the Mustelidae BMR deviation are the fundamental propulsion frequency constant, c, the mitochondrion capability quotient, e and the dynamic similarity constant, k. Because many Mustelidae combine swimming with terrestrial locomotion it is possible that their skeletal musculature may not be dynamically similar to other Carnivora and the constant k may be different from a value of 1.0. However, by Eq. (3), k and c cannot be separated without additional information. The product kc and e were varied to obtain the mustelid’s geometric similarity MMLE sturdiness factor boundaries shown in Fig. 3. Using kc = 1.4 m/s as was done for Proboscidea and Perissodactyla required a mitochondrion capability quotient at least 170% of that applicable to other Carnivora. That a mustelid mitochondrion would be this much more powerful than other carnivoran mitochondria is difficult to believe. Using a value of kc twice as large as that used for Perissodactyla and Proboscidea reduced the required mitochondrion capability quotient to 130% of that for other carnivorans. An implication would be that more powerful mitochondria allow mustelids to move their limbs twice as fast as other placental mammals in performing routine locomotion tasks.

Coverage, *R*, is the fraction of samples that fall between the MMLE sturdiness boundaries. Separating the Mustelidae from the rest of the Carnivora increased *R* for the entire Carnivora order from about 0.44 to 0.53 for *y* = 2/3 and from about 0.39 to 0.49 for *y* = 0.8. Although coverage is somewhat sparse, many of the samples lie close to the MMLE sturdiness boundaries as the greater ${R}_{M}^{2}$ values indicate.

For the groups for which it could be determined, Table 2 shows which skeletal musculature and which non-skeletal musculature similarity models are applicable to which MMLE homogeneous group for the running/walking placental mammals and for other groups that will be addressed soon.

**Table 2:** Similarity models applicable to MMLE homogeneous groups. The similarities are indicated by the regression analysis results reported in Tables 1, 3, and 4. Total body mass is the sum of the masses of the skeletal and non-skeletal musculatures.

MMLE homogeneous group	Skeletal musculature similarity model	Non-skeletal musculature similarity model
Artiodactyla + Carnivora less Mustelidae	Froude–Strouhal	Geometric^a^
Artiodactyla + Carnivora less Mustelidae (alternate)	Froude–Strouhal	Non-Geometric^b^
Mustelidae less Enhydra	Geometric^c^	Geometric
Non-Cricetidae Rodentia	Mixture of Geometric^c^ and Froude–Strouhal	Geometric
Cricetidae	Geometric^c^	Geometric
Light bats	Geometric^d^	Geometric
Heavy bats	Strouhal	Geometric
Intermediate bats	Mixture of Geometric^d^ and Strouhal	Geometric

**Notes.**

a Similarity indicated by the results of the cohort averaging regression method (AVG) results.

b Similarity indicated by the results of the phylogenetic informed regression method (PI) results.

c Compatible with Froude dynamic similarity.

d Compatible with Strouhal dynamic similarity.

## Rodentia Results

Rodentia comprise about 20% of families, 39% of genera, and 43% of species of recent mammals. Their masses range over four and a half orders of magnitude and their head and body lengths range over one and a half orders of magnitude (*Mus minutoides* with a mass of 2.5 g and head and body length of 45 mm to *Hydrochaeris hydrochaeris* with a mass of 79,000 g and head and body length of 1,300 mm). Various species employ scurrying, climbing, gliding, hopping, burrowing, swimming, running/walking, and combinations of these as their primary means of locomotion (Nowak, 1999).

Froude similarity should apply to skeletal muscle dynamics in most Rodentia as gravity is the main force affecting their locomotion. This should be true even for swimming as aquatic Rodentia are mainly surface swimmers that experience significant drag through the generation of surface waves in the wake. Drag through the generation of surface waves in the wake is the classic situation to which Froude similarity applies (Newman, 1977). What should govern the dynamics of burrowing is not clear, but as will be seen Froude similarity seems to work. The mass regressed on head and body length slope for both PI and AVG regressions for all families of Rodentia trends toward the geometric slope of 3.0. The combination of all families except Cricetidae trend toward an intermediate slope between the Froude–Strouhal slope of around 2.55 for mammals the size of Rodentia and the geometric slope. Cricetidae have an AVG mass regressed on length slope nearer the geometric similarity slope and the PI slope exceeds geometric similarity. Cricetidae also have a PI BMR regressed on mass slope that is not significantly different than the slope for geometric similarity as the log likelihood ratio for the slopes is 4.0.

Besides appearing to be more geometrically similar, cricetids tend to have a higher BMR when compared to non-cricetids of the same mass. For these reasons Cricetidae were analyzed separate from all the other families of Rodentia.

The slope values for mass regressed on head and body mass in Table 3 for both PI and AVG regressions are very different from the value of 2.5 obtained for *y* = 0.8 for Artiodactyla + Carnivora. They indicate either geometric similarity with *y* = 2/3 or a mixture of geometric similarity and Froude–Strouhal similarity with *y* = 2/3. For non-Cricetidae, the PI regression slope is not significantly different from the AVG slope as the log likelihood ratio for the two slopes is less than 4.0. For these reasons the geometric similarity value for the non-skeletal muscle exponent of *y* = 2/3 is used in Eq. (3) for Rodentia.

**Table 3:** Results of regression analyses for rodentia. The regression expressions are: Log(dependent variable) = slope X log(independent variable) + intercept.

Family	Regression type	Independent variable	Dependent variable	Slope	Intercept	*R* ^2^	Number samples
All Rodentia	PI(0.62)	Mass (g)	BMR (watts)	0.7231	−1.7198	0.8968	267
	PI(0.0)	Length (mm)	Mass (g)	2.9482	−4.3637	0.9571	105
	AVG	Length (mm)	Mass (g)	2.8692	−4.1497	0.9956	NA
Non-	PI(0.44)	Mass (g)	BMR (watts)	0.7399	−1.7685	0.9192	176
Cricetidae	PI(0.0)	Length (mm)	Mass (g)	2.9079	−4.2548	0.9571	78
	AVG	Length (mm)	Mass (g)	2.8564	−4.1124	0.9939	NA
Cricetidae	PI(0.55)	Mass (g)	BMR (watts)	0.6597	−1.5408	0.8497	91
	PI(0.0)	Length (mm)	Mass (g)	3.4061	−5.3367	0.9395	27
	AVG	Length (mm)	Mass (g)	2.9531	−4.3561	0.9908	NA

**Notes.**

PI(*n*) means the phylogenetic informed regression method using BayesTraits and the number in parentheses is the estimated value of lambda. AVG means the cohort averaging regression method. Length (mm) is head and body length in millimeters. Mass (g) is body mass in grams. NA means Not Applicable.

The skeletal musculature and non-skeletal musculature similarity models that are applicable to the rodent MMLE homogeneous groups are shown in Table 2.

Given the available data, linearly relating head and body length to characteristic length was tried. The characteristic length for Rodentia was assumed to be a constant fraction of head and body length. The fraction’s value was estimated by equating the combined Artiodactyla and Carnivora mass regressed on length expression to the all families of Rodentia mass regressed on head and body length for the range of lengths for Rodentia. This results in a fraction that is within the range 0.4 to 0.62 using the PI regression relationship and 0.43 to 0.61 using the AVG relationship. A value of 0.5 was considered to be a reasonable working estimate for this characteristic length scaling fraction.

The characteristic length scaling fraction adds an additional parameter to the number that MMLE uses to predict the absolute values of body mass and BMR.

The fundamental locomotion frequency for geometric similarity is a constant divided by the characteristic length. The value of 1.4 m/s for the constant that worked well for Perissodactyla and Proboscidea was also used as the constant for Rodentia.

**Figure 4:**
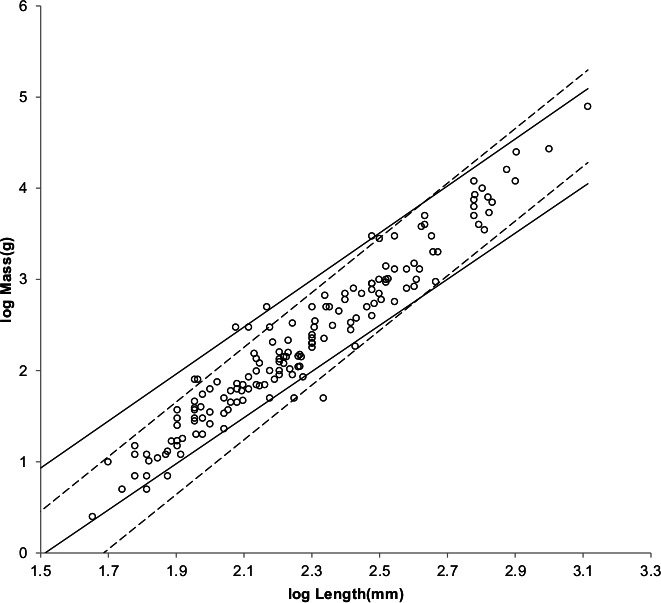
Log body mass as a function of log head and body length for non-cricetid rodents. Data are from Nowak (1999). The solid and dashed lines are MMLE sturdiness factor boundaries. The upper boundaries were generated with a sturdiness factor *s* = (3)^0.5^. The lower boundaries were generated with *s* = (3)^−0.5^. The shallower sloping solid boundary lines are for Froude–Strouhal similarity. The steeper sloping dashed boundary lines are for geometric similarity. Non-cricetid rodents are marked by open circles. ${R}_{M}^{2}=0.9995$ with respect to both sets of boundaries.

Figure 4 shows the MMLE mass as a function of head and body length sturdiness factor boundaries for Froude–Strouhal dynamic similarity and geometric similarity evaluated with the same constants that were used for running/walking mammals and a characteristic length scaling factor of 0.5.

**Figure 5:**
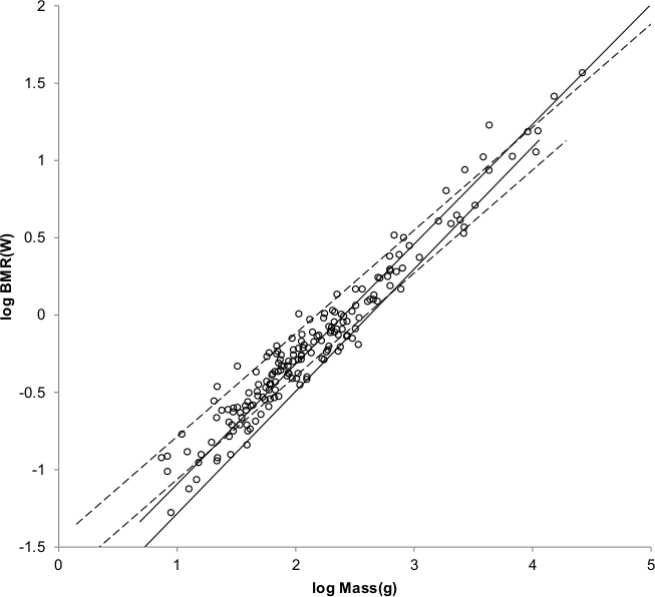
Log BMR as a function of log body mass for non-cricetid rodents. Data are from Kolokotrones et al. (2010). The solid and dashed lines are MMLE sturdiness factor boundaries. The upper boundaries were generated with a sturdiness factor *s* = (3)^0.5^. The lower boundaries were generated with *s* = (3)^−0.5^. The steeper sloping solid boundary lines are for Froude–Strouhal similarity. The shallower sloping dashed boundary lines are for geometric similarity. The species-averaged non-Cricetidae Rodentia are marked by open circles. ${R}_{M}^{2}=0.9966$ with respect to both sets of boundaries.

Figure 5 shows the MMLE BMR as a function of body mass sturdiness factor boundaries for the two similarity regimes evaluated with the same constants that were used for non-Mustelidae Carnivora and a characteristic length scaling factor of 0.5.

**Figure 6:**
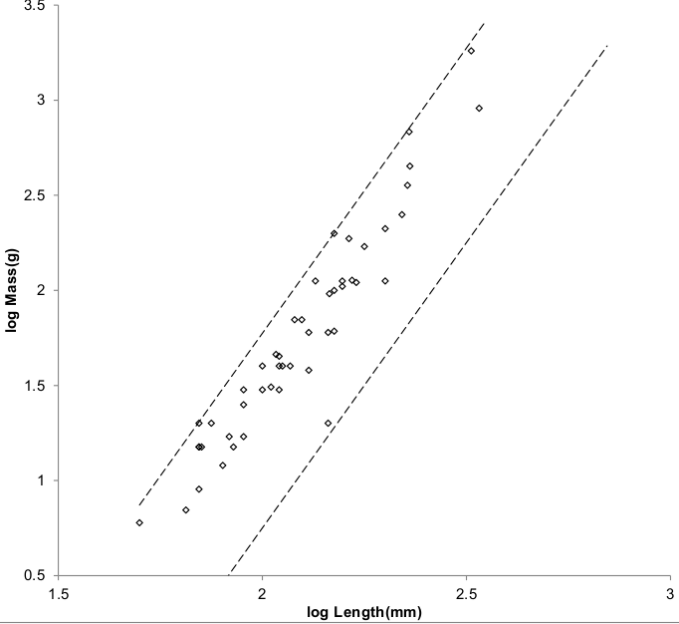
Log body mass as a function of log head and body length for Cricetidae. The data are from Nowak (1999). The dashed lines are MMLE sturdiness factor boundaries. The upper boundary was generated with a sturdiness factor *s* = (3)^0.5^. The lower boundary was generated with *s* = (3)^−0.5^. The boundary lines are for geometric similarity. Cricetids are marked by open diamonds. ${R}_{M}^{2}=1.0$.

**Figure 7:**
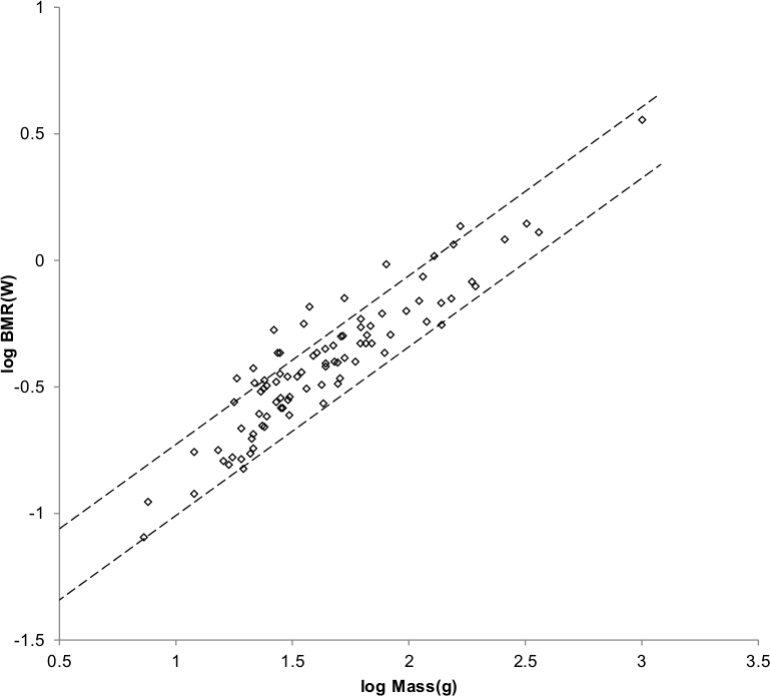
Log BMR as a function of log body mass for Cricetidae. The data are from Kolokotrones et al. (2010). The dashed lines are MMLE sturdiness factor boundaries. The upper boundary was generated with a sturdiness factor *s* = (3)^0.5^. The lower boundary was generated with *s* = (3)^−0.5^. The boundary lines are for geometric similarity. Cricetid species-averaged BMR, mass data are marked by open diamonds. ${R}_{M}^{2}=0.9913$.

As with Mustelidae that have a greater BMR at the same body mass than do other Carnivora, a greater mitochondrion capability quotient would most straight forwardly result in a greater BMR for Cricetidae with the same masses as other Rodentia. Varying it until a maximum value of ${R}_{M}^{2}$ was achieved for both mass as a function of length and BMR as a function of mass resulted in a mitochondrion capability quotient of 1.2. Figure 6 shows the MMLE mass as a function of head and body length sturdiness factor boundaries for geometric similarity evaluated with this mitochondrion capability quotient value. Figure 7 shows the MMLE BMR as a function of body mass sturdiness factor boundaries.

A mitochondrion capability quotient of 1.2 as an explanation of why Cricetidae have an elevated BMR with respect to other Rodentia is considerably more palatable than the value of this parameter needed to explain the elevated BMR of Mustelidae with respect to other Carnivora.

## Bats (the Order Chiroptera) Results

Bats are second only to rodents in the number of species among mammals. Bats comprise about 12% of families, 16% of genera, and 20% of species of recent Mammals. Their masses range over three orders of magnitude from *Craseonycteris thonglongyai* and *Tylonycteris pachypus* with masses as small as 2 g to *Pteropus giganteus* with a mass of as much as 1,600 g (Nowak, 1999).

Bats primary means of locomotion is flying by flapping very flexible membranous wings controlled by multi-jointed fingers (Muijres et al., 2011). Unlike birds, bats use their hind limbs as well as their fore limbs to flap their wings (Norberg, 1981). Bats experience daily and seasonal fluctuations in body mass which they accommodate by changes in wing kinematics that vary among individuals (Iriarte-Diaz et al., 2012). To analyze the applicability of MMLE theory to bats, a characteristic length and a fundamental propulsion frequency related to very complicated flapping wing flight needed to be identified. The characteristic length should be related to wing dimensions. Norberg (1981) found that forearm length scaled with body mass with about the same exponent as wing span. Given the options available with the Nowak (1999) data, it was assumed that forearm length is linearly related to characteristic length. A possible complication that is avoided by this assumption is that full wing dimensions, such as wing span, in a flying bat may vary with flight mode and speed and may be different than those measured from specimens stretched out flat on a horizontal surface (Riskin et al., 2010).

Norberg & Rayner (1987) found that geometric similarity applied for most bat wing dimensions with some exceptions. More recent work suggests that wing bone lengths are also geometrically similar with respect to body mass in different sized bats (Norberg & Norberg, 2012).

The order Chiroptera is divided into two suborders: the Megachiroptera consisting of the single family Peteropodidae and the Microchiroptera consisting of all other bats (Nowak, 1999). Table 4 shows the regression analysis results obtained with forearm length data and BMR data for all bats and for the two suborders considered separately. The geometric similarity non-skeletal muscle mass exponent value of *y* = 2/3 is consistent with both the PI and AVG all bats results and the Megachiroptera results. The non-geometric value of *y* = 0.8 is consistent with both PI and AVG results for Microchiroptera.

**Table 4:** Results of bat regression analyses. The regression expressions are: log(dependent variable) = slope × log(independent variable) + intercept.

Order or family	Regression type	Independent variable	Dependent variable	Slope	Intercept	*R* ^2^	Number samples
All bats	PI(0.93)	Length (mm)	Mass (g)	2.6668	−3.3211	0.8507	176
	AVG	Length (mm)	Mass (g)	2.7718	−3.3628	0.9924	NA
Megachiroptera	PI(0.33)	Length (mm)	Mass (g)	2.8628	−3.4933	0.9749	40
	AVG	Length (mm)	Mass (g)	2.7335	−3.2632	0.9978	NA
Microchiroptera	PI(0.93)	Length (mm)	Mass (g)	2.515	−3.1025	0.7522	136
	AVG	Length (mm)	Mass (g)	2.5292	−2.9844	0.9928	NA
Heavy bats	PI(0.79)	Length (mm)	Mass (g)	2.7595	−3.3252	0.922	86
	AVG	Length (mm)	Mass (g)	2.7233	−3.2329	0.9937	NA
Light bats	PI(0.0)	Length (mm)	Mass (g)	3.28	−4.4926	0.8781	29
	AVG	Length (mm)	Mass (g)	2.9878	−3.9552	0.9743	NA
All bats	PI(0.83)	Mass (g)	BMR (watts)	0.8111	−1.9063	0.8784	84
Megachiroptera	PI(0.56)	Mass (g)	BMR (watts)	0.8581	−2.0161	0.9279	21
Microchiroptera	PI(1.07)	Mass (g)	BMR (watts)	0.7459	−1.8247	0.991	63
Heavy bats	PI(0.89)	Mass (g)	BMR (watts)	0.8225	−1.9166	0.8887	51
Light Bats^a^	PI(0.0)	Mass (g)	BMR (watts)	0.7015	−1.8048	0.8154	17

**Notes.**

a Data available for only 4 of the 8 families comprising light bats.

PI(*n*) means the phylogenetic informed regression method using BayesTraits and the number in parentheses is the estimated value of lambda. AVG means the cohort averaging regression method. Length (mm) is forearm length in millimeters. Mass (g) is body mass in grams. NA means Not Applicable.

**Figure 8:**
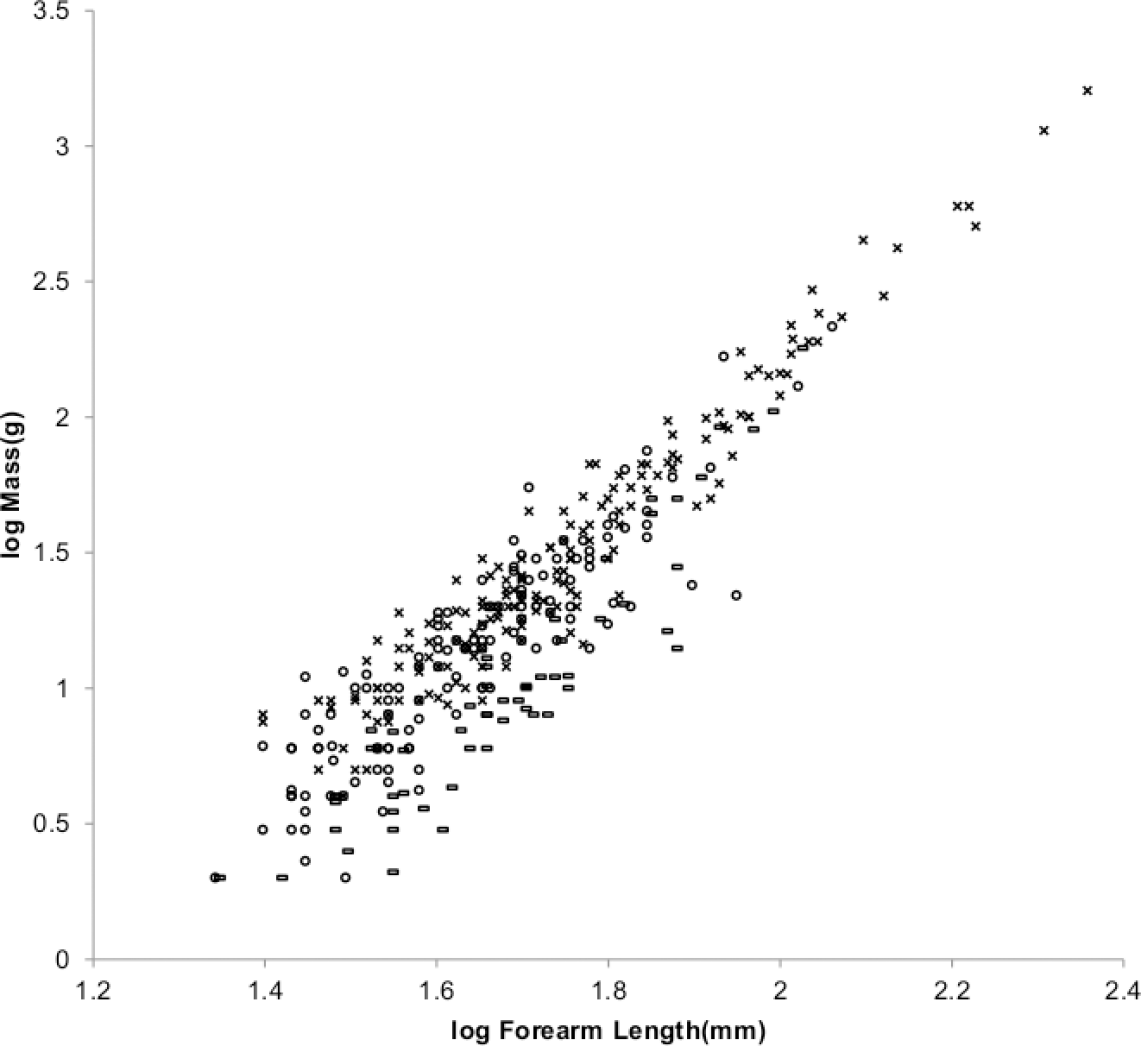
Log body mass as a function of log forearm length for bats. At the same forearm length individuals from families composing the ‘heavy’ bats marked with Xes are mostly more massive than those from the families composing the ‘light’ bats marked with open rectangles. ‘Intermediate’ bats marked with open circles span both the heavy and light mass regimes. The families Pteropodidae and Phyllostomidae comprise the heavy bats. The families Emballonuridae, Craseonycteridae, Rhinopomatidae, Rhinolophoidea, Mormoopidae, Noctilionidae, Furipteridae and Hipposideridae comprise the light bats. The families Nycteridae, Megadermatidae, Vespertilionoidae, Thyropteridae, Myzopodidae, Natalidae, Mystacinidae, and Molossidae comprise the intermediate bats.

Figure 8 offers an alternative partitioning for bats in which the bat families have been divided into three groups: ‘heavy’ bats, ‘light’ bats and ‘intermediate’ bats. At the same forearm length members of families composing the heavy bats are mostly more massive than those of the families composing the light bats. Intermediate bats span both the heavy and light mass regimes. The families composing the three groups are given in the caption of Fig. 8.

For the AVG regression of log mass on log forearm length the slope for light bats is very nearly the 3.0 expected for geometric similarity and the slope of the PI regression relationship is not significantly different from 3.0 as the log likelihood ratio is only 2.7. Light bats appear to be geometrically similar.

Pteropodidae and Phyllostomidae comprise the heavy bats. The Pteropodidae contain the Old World frugivores and the Phyllostomidae contain the New World frugivores. While both families have species with other diets, the frugivores have wings adapted to commuting long distances from roost to feeding areas (Norberg & Rayner, 1987). The relationships between wing dimensions and body mass should be similar for the frugivore members of the two families.

The fundamental propulsion frequency for bats should be related to the wing flapping frequency. A bat’s wing flapping frequency increases slightly with air speed at lower speeds. It becomes almost independent of speed at higher speeds. The speed at which the transition occurs is the preferred speed (Bullen & McKenzie, 2002). The wing flapping frequency at this preferred speed should be proportional to the fundamental propulsion frequency.

Animals that fly by flapping wings operate in a narrow range of Strouhal numbers in cruising flight (Taylor, Nudds & Thomas, 2003). Strouhal number does not change significantly for Pteropodidae (Riskin et al., 2010). It appears to be an accurate predictor of wingbeat frequency for birds (Nudds, Taylor & Thomas, 2004). Strouhal dynamic similarity should apply to bats.

For bats Strouhal number = (wingbeat frequency) × (wingbeat amplitude)/(air speed). Wingbeat amplitude is the vertical distance the wing tip travels during a stroke. Under Strouhal dynamic similarity the wingbeat amplitude should be proportional to the MMLE characteristic length. It was assumed that wingbeat amplitude characteristic length is a fraction of forearm length for bats. Further analysis involving maximization of ${R}_{M}^{2}$ for light bat BMR resulted with characteristic length = 0.61 of forearm length, kc = 0.625 m/s and ${R}_{M}^{2}=0.9984$ (see Article S1).

Strouhal dynamic similarity is consistent with geometric similarity. Since the fundamental propulsion frequency under geometric similarity is proportional to the inverse of the characteristic length, simultaneous geometric and Strouhal similarity imply that the preferred flight speed is approximately constant.

Since *R*^2^ is nearly unity for the AVG regression relationship of Table 4, Eq. (6) should apply. The log likelihood for the resulting exponent, 2/*x* = 0.6694 could not be calculated using BayesTraits. Thus the significance of its difference from the BMR regressed on mass exponent in Table 4 could not be determined. It is noted that length and mass data was available to estimate *x* for all families composing the light bats group but BMR and mass data to estimate the BMR regressed on mass exponent was available for only four of the eight families.

**Figure 9:**
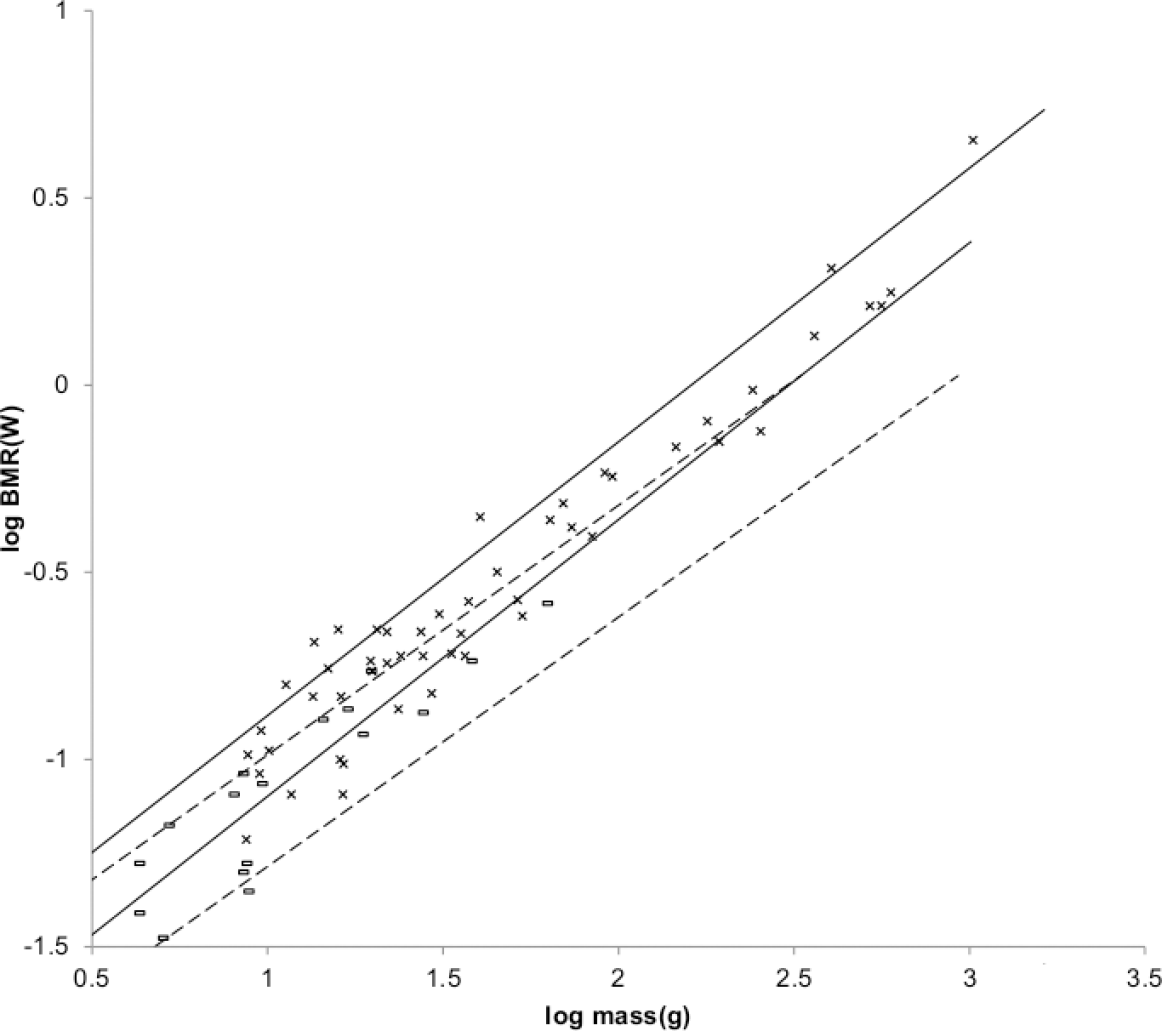
Log BMR as a function of log body mass for heavy and light bats. The solid and dashed lines are the MMLE sturdiness factor boundaries. The upper boundaries were generated with a sturdiness factor *s* = (3)^0.5^. The lower boundaries were generated with *s* = (3)^−0.5^. The steeper sloping solid boundary lines are for heavy bat Strouhal dynamic similarity. The shallower sloping dashed boundary lines are for light bat geometric similarity. ${R}_{M}^{2}=0.9828$ for heavy bats with respect to the Strouhal boundaries. ${R}_{M}^{2}=0.9984$ for light bats with respect to the geometric boundaries. Heavy bat species-averaged data are marked with Xes. Light bats species-averaged data are marked with open rectangles. Data was available for only four of the eight families comprising the light bats.

Figure 9 shows the MMLE BMR as a function of body mass boundaries for geometric similarity for light bats evaluated with these estimates.

A methodology similar to that used for light bats was used to establish the characteristic length scaling fraction and fundamental propulsion frequency constant for heavy bats. By the heavy bat regression relationships of Table 4, heavy bats are not geometrically similar. Since heavy bats are not geometrically similar the exponent, *r*, in the relationship between fundamental propulsion frequency and characteristic length in Eqs. (3) and (5) also had to be established. The results using the Table 4 AVG relationships were characteristic length = 0.92 forearm length, *r* = 0.68, kc = 3.22 m^0.68^/s and ${R}_{M}^{2}$ for BMR = 0.9828. The results using the PI relationships were characteristic length = 0.86 forearm length, *r* = 0.73, kc = 2.59 m^0.73^/s, and ${R}_{M}^{2}$ for BMR = 0.9918. In terms of ${R}_{M}^{2}$ the PI and AVG results are nearly indistinguishable (see Article S1).

Since *R*^2^ is nearly unity for the heavy bat AVG mass regressed on length relationship in Table 4, Eq. (6) should apply. The difference between the resulting exponent, 2/*x* = 0.7344 and the PI BMR regressed on mass exponent in Table 4 is borderline significant as the log likelihood ratio is 4.1.

Figure 9 also shows the MMLE log BMR as a function of log body mass MMLE sturdiness factor boundaries for heavy bats evaluated with these estimates.

**Figure 10:**
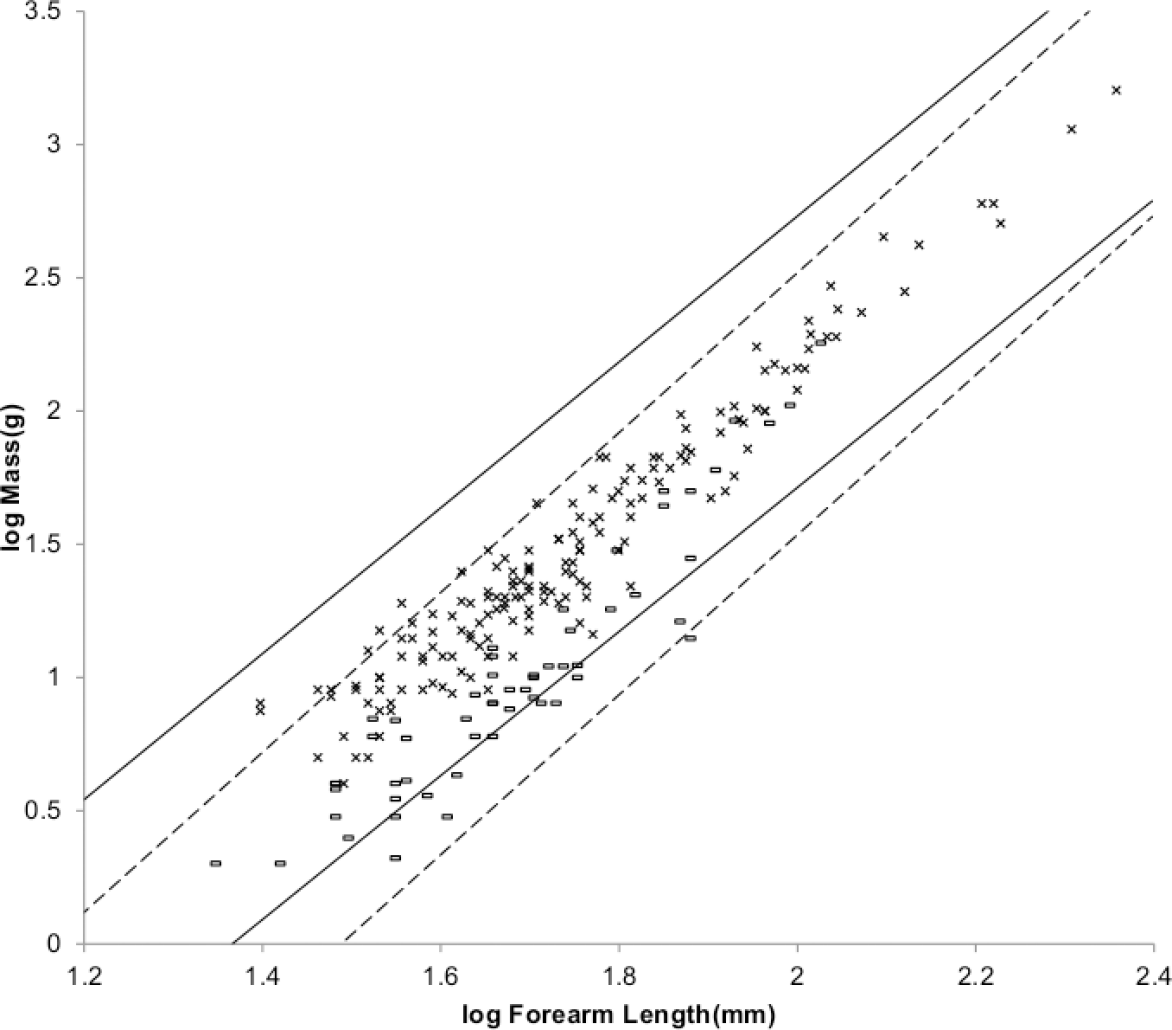
Log body mass as a function of log forearm length for heavy and light bats. The solid and dashed lines are the MMLE sturdiness factor boundaries. The upper boundaries were generated with a sturdiness factor *s* = (3)^0.5^. The lower boundaries were generated with *s* = (3)^−0.5^. The shallower sloping solid boundary lines are for heavy bat Strouhal dynamic similarity. The steeper sloping dashed boundary lines are for light bat geometric similarity. ${R}_{M}^{2}=1.0$ for heavy bats with respect to the Strouhal boundaries. ${R}_{M}^{2}=1.0$ for light bats with respect to the geometric boundaries. Individual heavy bats are marked with Xes. Individual light bats are marked with open rectangles.

Figure 10 shows the MMLE log body mass as a function of log forearm length MMLE sturdiness factor boundaries for both heavy bats and geometrically similar light bats.

**Figure 11:**
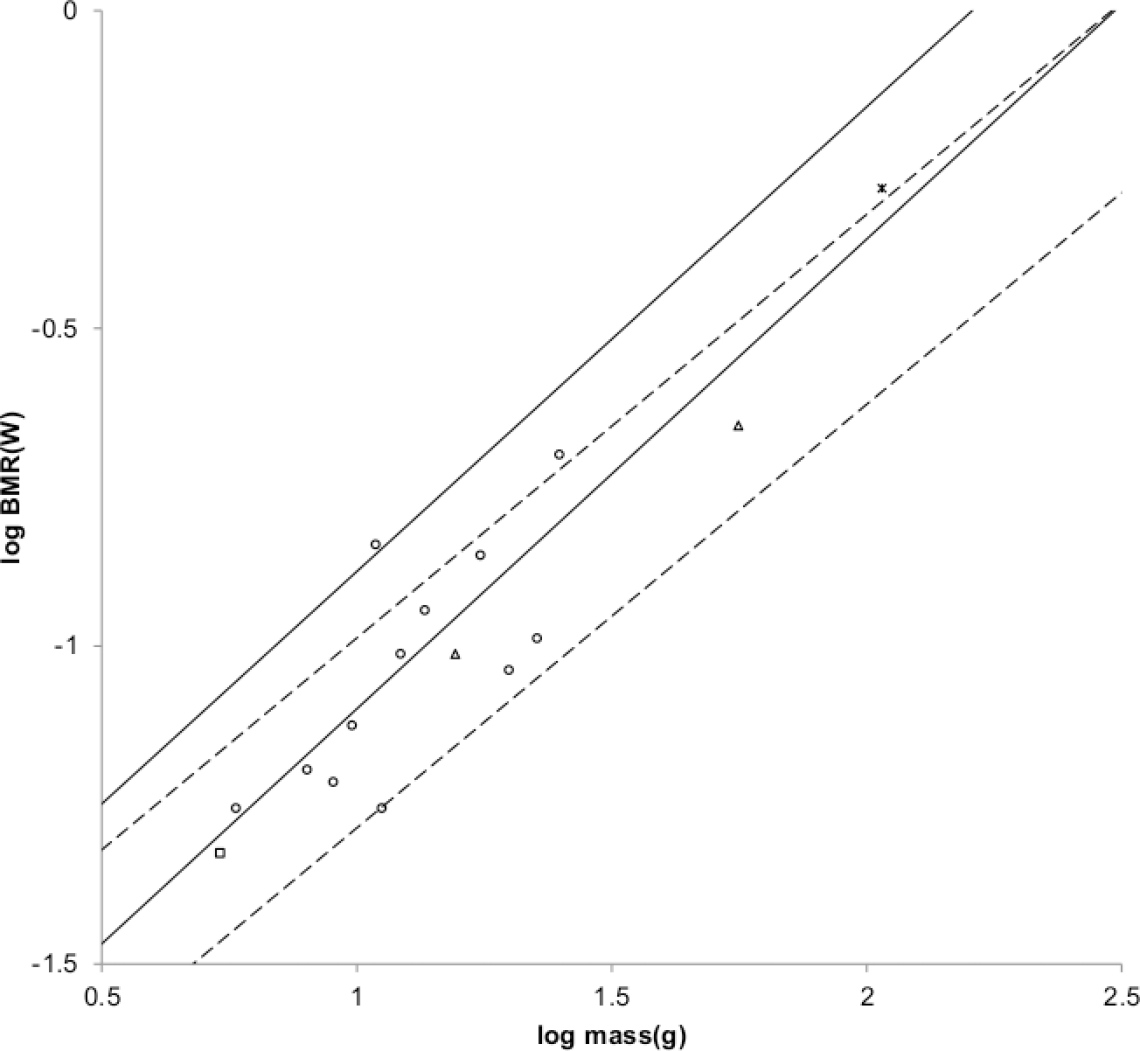
Log BMR as a function of log body mass for intermediate bats. The solid and dashed lines are the MMLE sturdiness factor boundaries. The upper boundaries were generated with a sturdiness factor *s* = (3)^0.5^. The lower boundaries were generated with *s* = (3)^−0.5^. The steeper sloping solid boundary lines are for heavy bat Strouhal dynamic similarity. The shallower sloping dashed boundary lines are for light bat geometric similarity. Data was available for only four of the eight families comprising the intermediate bats. ${R}_{M}^{2}=0.9678$ for intermediate bats with respect to the Strouhal boundaries. ${R}_{M}^{2}=0.9893$ for intermediate bats with respect to the geometric boundaries. ${R}_{M}^{2}=0.9998$ for intermediate bats with respect to both sets of boundaries. The data are species-averaged. Megadermatidae are marked with crossed Xes. Molossidae are marked with open triangles. Vesperstilionidae are marked with open circles. Natalidae are marked with open squares.

Figure 11 shows the MMLE log BMR as a function of log body mass MMLE sturdiness factor boundaries for both heavy bats and light bats. Species averaged BMR and body mass samples for intermediate bats are also shown. The data spreads over both MMLE bands as if within the same family there are species that conform to geometric similarity and other species that conform to heavy bat Strouhal similarity.

**Figure 12:**
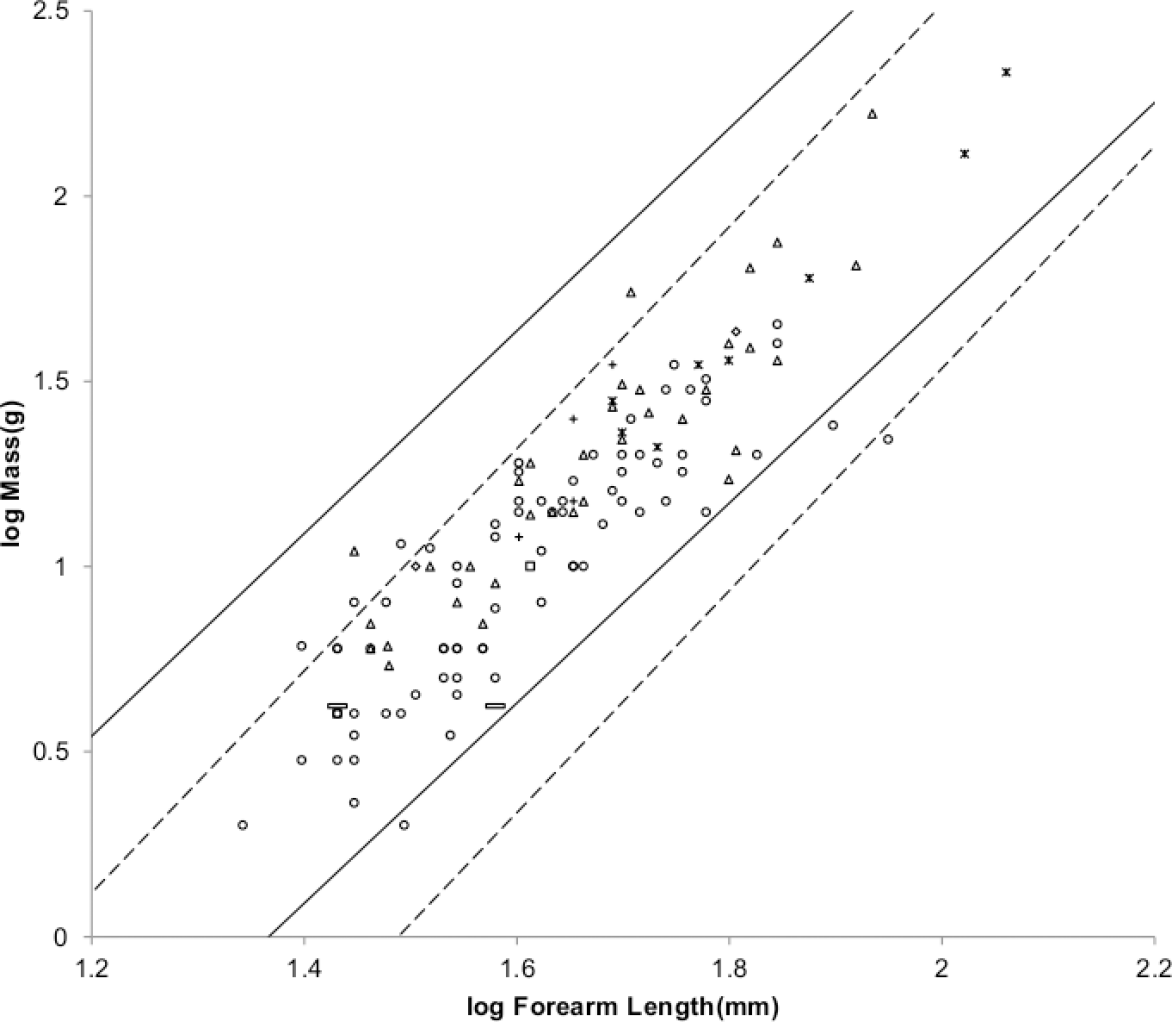
Log body mass as a function of log forearm length for intermediate bats. The solid and dashed lines are the MMLE sturdiness factor boundaries. The upper boundaries were generated with a sturdiness factor *s* = (3)^0.5^. The lower boundaries were generated with *s* = (3)^−0.5^. The shallower sloping solid boundary lines are for heavy bat Strouhal dynamic similarity. The steeper sloping dashed boundary lines are for geometric similarity. ${R}_{M}^{2}=0.998$ for intermediate bats with respect to the Strouhal boundaries. ${R}_{M}^{2}=0.9981$ for intermediate bats with respect to the geometric boundaries. ${R}_{M}^{2}=0.9999$ for intermediate bats with respect to both sets of boundaries.

Figure 12 shows the MMLE log body mass as a function of log forearm length MMLE sturdiness factor boundaries for both heavy and light bats. Body mass and forearm length samples for intermediate bats are also shown. Intermediate bat body mass is explained almost equally well by either the geometrically similar light bat MMLE band or the heavy bat MMLE band as the ${R}_{M}^{2}$ values indicate.

The light and heavy bat data in Fig. 10 does not fully occupy their respective MMLE sturdiness factor bands. This raises the possibility that a sturdiness factor range narrower than (3)^−0.5^ to (3)^0.5^ may apply to heavy or light bats. The intermediate bat data in Fig. 12 more completely occupies both bands indicating that the full sturdiness factor range is applicable. The situation may be more complicated. Each of the heavy, light and intermediate bat groups contains species with different food habits. Bat wing morphology is associated with flight behavior related to food habits. Even among the insectivores that dominate the light and intermediate groups there are different styles of catching insects that are associated with differing wing morphologies (Norberg & Rayner, 1987; Norberg & Norberg, 2012). Dynamic similarity may apply at a phylogenetic level below the family.

The light-intermediate-heavy representation of bats is better supported by the data than either the all bats representation or the Megachiroptera-Microchiroptera representation in terms of ${R}_{M}^{2}$ and coverage, *R* (see Article S1). Table 2 shows which skeletal musculature and which non-skeletal musculature similarity models are applicable to the light, heavy and intermediate bat MMLE homogeneous groups.

## Computation Error

With a body mass and characteristic length data set such as Nowak (1999), Eq. (3) is exact for a MMLE homogeneous group because for every datum a value for the sturdiness factor can be found such that the body mass is exactly computed by the equation using this found sturdiness factor and the characteristic length. With a BMR and body mass data set such as Kolokotrones et al. (2010), Eqs. (2) and (3) are exact because for every datum a value for the sturdiness factor and a value for the characteristic length can be found such that the BMR is exactly computed by Eq. (2) and the body mass is exactly computed by Eq. (3) using the found sturdiness factor value and the found characteristic length value. For the same reasons, Eqs. (2) and (3) are exact with other MMLE homogeneous groups once the non-characteristic length and non-sturdiness factor parameters that make the groups different are determined.

The ability of MMLE to exactly compute every datum obtained from the appropriate data set does not necessarily mean that there is no unexplained variance with MMLE theory. Equations 2 and (3) apply simultaneously to a particular individual animal which has particular values for body mass, BMR, and characteristic length. The author of the present paper is unaware of a data set that reports all three of these values for individual animals. Instead there is the data set from Nowak (1999) reporting individual body mass and characteristic length and another data set from Kolokotrones et al. (2010) that reports species-averaged BMR and body mass. It is unlikely that the same particular animal is included in both data sets. There is likely to be unexplained variance with a data set that simultaneously reports the three values of mass, BMR and length for individual animals. The unexplained variance would be detected by the need to use one value of the sturdiness factor in Eq. (3) to accurately compute mass and a different value in Eq. (2) to accurately compute BMR for the same animal.

That there could be unexplained variance with a data set that reports the three values of mass, BMR and length for individual animals is suspected because of measurement errors and because MMLE theory as currently expressed does not account for body temperature effects on the mitochondrion capability quotient. Body temperature has a significant effect on BMR (Clarke, Rothery & Isaac, 2010; Kolokotrones et al., 2010).

However, as shown in Table 5, MMLE is able to predict the BMR regressed on mass exponent obtained by PI regression analysis of BMR and mass data from the mass regressed on length exponent obtained by AVG regression analysis of length and mass data for many of the MMLE homogeneous groups addressed in this paper. This is a result of being able to use Eq. (6) because *R*^2^ is nearly unity for the AVG regression analyses. This might not be significant for the multiplicative constants in Eqs. (2) and (6) as the regression analyses for both data sets were used to estimate the value of *G_r_* that occurs in both equations. But the exponents are independent of the multiplicative constants. These considerations argue that MMLE may be able to simultaneously compute an individual animal’s mass and BMR given its characteristic length and sturdiness factor; or looked at another way, it may be possible to find sturdiness factors so that MMLE can compute every datum from a data set that simultaneously reports individual animals’ mass, characteristic length and BMR.

**Table 5:** Comparison of the theoretical value of the exponent, *b*, with the value obtained by PI regression analysis for the equation BMR = *aW^b^* for MMLE homogeneous groups. The exponents from PI regression analyses, *b*, are the slope values reported in Tables 1, 3 and 4 for the PI regression type with independent variable Mass (g) and dependent variable BMR (watts). The theoretical exponent is either (1) the value assuming geometric similarity or (2) the Eq. (6) value, 2/*x*, where *x* is the slope value reported in Tables 1, 3 or 4 for the AVG regression type with independent variable Height (m) or Length (mm) and dependent variable Mass (g). The values for *x* were obtained by analyses of data from Nowak (1999) by the cohort averaging regression method (AVG). The PI regression analysis exponents were obtained by Phylogenetically Informed (PI) regression analyses of Kolokotrones et al. (2010) data using BayesTraits. A log likelihood ratio less than 4.0 means that an exponent from the PI regression analysis column is not significantly different from the corresponding exponent from the theoretical exponent column (Pagel, 1999).

MMLE homogeneous group	Exponent from PI regression analysis, *b*	Theoretical exponent	Log likelihood ratio	Significant difference?
Carnivora less Mustelidae	0.758	0.7651^(2)^	0.1	N
Ruminant artiodactyls	0.7805	0.7651^(2)^	2.8	N
Mustelidae less Enhydra	0.6852	0.6667^(1)^	0.4	N
Cricetidae Rodentia	0.6597	0.6773^(2)^	17.8	Y
Cricetidae Rodentia	0.6597	0.6667^(1)^	4.0	N
Heavy bats	0.8225	0.7344^(2)^	4.1	Y/N
Light bats	0.7015	0.6694^(2)^	NC	NC

**Notes.**

N means “no”, Y means “yes” and Y/N means borderline. NC means that a log likelihood ratio could not be calculated for the Light bats group.

## Discussion

10 parameters were theoretically derived to compute BMR and body mass with MMLE (11 parameters if a characteristic length scaling factor is needed). The parameters emerged from the theoretical derivation of MMLE from the principles of physics and physiology that were being considered. 10 parameters are only one more than the number of factors that McNab (2008) found influenced mammal BMR; and it is less than the over 22 parameters needed to best describe the overall mass and temperature dependence of BMR by the White, Frappell & Chown (2012) information-theoretic analysis. While that analysis did include insects, spiders, protists and prokaryotes as well as vertebrates, it and McNab’s findings do indicate that needing a large number of parameters to predict the absolute value of a vertebrate’s body mass and BMR should not be surprising. Even describing the relationships between BMR and body mass, W, and a skeletal dimension, *l*, for a collection of animals with the simple relationships BMR = *aW^b^* and *W* = *dl^x^* requires at least five parameters and as many as six if BMR and length data are not for the same individual animals. Simplicity is not necessarily better than complexity (White, Frappell & Chown, 2012).

Finding new values for the fundamental propulsion frequency constant, c, the mitochondrion capability quotient, e and the dynamic similarity constant, k to reconcile Cricetidae with the rest of Rodentia and to reconcile Mustelidae with the rest of Carnivora or the division of bats into three new groups could appear to be forcing MMLE to fit the data. However these instances are legitimate uses of the degrees of freedom available with MMLE to explain differences that were observed in the data.

The results of regression analyses were used to estimate numerical values for the parameters. Phylogenetic informed (PI) regression analysis was performed on taxon-averaged data using BayesTraits. AVG regression analysis was performed on individual body mass, characteristic length data. AVG regression is unique to MMLE theory. Parameter numerical values estimated from results obtained with the two regression techniques did differ. In terms of the two MMLE measures of effectiveness, ${R}_{M}^{2}$ and R, body masses computed by Eq. (3) and BMR computed by Eq. (2) are nearly indistinguishable when using parameters estimated from the results of either technique.

The values for the constants *G_m_*, *G_o_* and *G_r_* were derived from running/walking placental mammal data. Since the values obtained in the present paper differ from the values obtained with the data that was available over a quarter of a century ago, it would not be surprising if the values should be refined again in the future.

Using Eq. (3) the ratio of volume active tissue mass to total body mass for geometrically similar running/walking placental mammals is calculated to vary from 81% for sturdy animals to 93% for gracile animals. For Strouhal–Froude dynamically similar animals the ratio varies from 87% for small sturdy animals to 91% for small gracile animals; and for large animals it varies from 61% for sturdy animals to 74% for gracile animals. The ratio of muscle to total body mass appears to vary between 21% and 61% for mammals (Muchlinski, Snodgrass & Terranova, 2012). The MMLE volume active tissues appear to be about twice as massive as the skeletal musculature. This leads to the possibility that inclusion of a third type of tissue in the MMLE representation of the vertebrate body may be warranted. The third tissue type would include everything that is not skeletal muscle, liver, heart, kidneys, and brain. It would have few mitochondria so that it would contribute little to BMR.

Three types of dynamic similarity were encountered for the skeletal musculature of the placental mammals examined in the present paper: Froude similarity, Strouhal similarity and simultaneous Froude and Strouhal similarity. Geometric similarity is compatible with either Froude or Strouhal similarity. It is not compatible with simultaneous Froude and Strouhal similarity. The type of dynamic similarity that governs the morphology of an animal’s skeletal musculature seems to correlate with a phylogenetic level that may be as low as the species.

Compared with the original paper (Frasier, 1984), the present paper makes important modifications to MMLE theory. The fundamental propulsion frequency is generalized to *f* = *c*/*l^r^* where c is the fundamental propulsion frequency constant, *l* is the characteristic length and *r* is the fundamental propulsion frequency exponent. This generalization allows the different types of dynamic similarity to be explicitly addressed by Eq. (3). The mass of the non-skeletal musculature is also generalized to allow non-geometrically similar topologies to be explicitly addressed by Eq. (3). Ordinary Least Squares regression analyses are replaced by PI analyses in the present paper. The present paper estimates new numerical values for the parameters occurring in Eqs. (2) and (3) by analysis of data that was unavailable when the original paper was written. The present paper furthermore extends the demonstration of MMLE theory’s ability to exactly compute body mass and BMR for individual animals from about 9% of placental mammals to about 72% by adding Rodentia and bats to running/walking Artiodactyla, Carnivora, Perissodactyla and Proboscidea.

Carnivora with body masses less than about 2,000 g were not included in the body mass as a function of characteristic length analysis of running/walking placental mammals. They were not included because their shoulder heights are not given in Nowak (1999). However head and body lengths are given. The methodology of using a scaling fraction to translate heady and body length to characteristic length as was done for Rodentia may also work for small Carnivora.

MMLE’s ability to compute BMR and body mass for the mammal orders that were not included in the present paper should be examined. A complication is that from an examination of Kolokotrones et al. (2010) it appears that BMR data are scarce except for the orders Primates, Soricomorpha, Dasyuromorpha and Diprotodontia.

MMLE should apply to all vertebrates. The original paper tried to address birds but that analysis now appears to be simplistic. The analysis should be performed again with recent data and the expanded version of MMLE presented in the present paper.

The sturdiness factor adds additional information about the composition of an animal’s body that is missing from relationships of the form *aW^b^*. Consideration of body composition can reduce unexplained variance even for relationships that express BMR as a function of body mass (Raichlen et al., 2010; Müller et al., 2011).

The Raichlen, Pontzer & Shapiro (2013) finding that hip joint to limb center of mass length is a better length for establishing the pendulum frequency for Froude–Strouhal similar animals may affect the concept of the sturdiness factor. Sturdier animals have stouter limbs which should cause the limb’s center of mass to be further from the hip joint and thereby increase the characteristic length. For this reason it is possible that the sturdiness factor may be, in part, a scaling factor for converting characteristic length surrogates such as shoulder height into hip joint to limb center of mass lengths.

MMLE theory as currently expressed does not account for body temperature effects on the mitochondrion capability quotient. Body temperature has a significant effect on BMR (Clarke, Rothery & Isaac, 2010; Kolokotrones et al., 2010). The extent to which the inclusion of temperature effects on the mitochondrion capability quotient would improve MMLE’s accuracy awaits a complete data set reporting body temperature as well as mass, BMR and length for individual animals. In the meantime, the body temperature data in Kolokotrones et al. (2010) could be used to examine the extent to which inclusion of temperature in Eq. (3) would narrow the sturdiness factor range needed to bound the Kolokotrones et al. (2010) BMR and mass data and the Nowak (1999) mass and length data.

While the mitochondrion capability quotient used in Eq. (3) should have approximately the same value for all animals with the same body temperature in the same phylogenetic group, genetic variation could cause it to vary among individuals or populations in the same species. Variation of the mitochondrion capability coefficient together with variation of the sturdiness factor would cause mass-independent variation in the BMR of animals like that recently addressed by White & Kearney (2013).

MMLE was derived for fit animals surviving in the wild. Captivity, domestication, inactivity and sickness could cause an animal’s body mass or BMR to be different from that predicted by MMLE. In particular, muscle or mitochondrion atrophy due to decreased activity with respect to that experienced in the wild would likely cause a difference.

## Conclusion

The purpose of this paper was to use modern resources to revisit MMLE theory and test its ability to compute the absolute values of BMR and body mass for individual animals. With some modifications to the theory as originally formulated (Frasier, 1984) MMLE can exactly compute every individual BMR given body mass datum and every individual body mass given characteristic length datum for the recent data sets that were examined (Nowak, 1999; Kolokotrones et al., 2010) and for the mammal orders examined. MMLE deterministically computes the absolute value of BMR and body mass for individual animals rather than a statistical best fit average value for a collection of individuals from a single species which is the usual methodology used by other examinations of these topics (White & Kearney, 2014). The examined mammal orders include over two thirds of recent mammal species.

Determining if MMLE can simultaneously exactly compute an individual animal’s BMR and body mass given its characteristic length awaits a data set that simultaneously reports all three values for individual animals. However MMLE’s ability to predict the BMR regressed on mass exponent obtained by PI regression analysis of BMR and mass data from the mass regressed on length exponent obtained by AVG regression analysis of length and mass data for many of the MMLE homogeneous groups addressed in this paper argues that accurate simultaneous prediction of mass and BMR for the same animal may be possible.

## Supplemental Information

10.7717/peerj.1228/supp-1 Data S1 Running/walking placental mammals masses and shoulder heights

10.7717/peerj.1228/supp-2 Data S2 Rodentia masses and lengths

10.7717/peerj.1228/supp-3 Data S3 Chiroptera (Bats) masses and lengths

10.7717/peerj.1228/supp-4 Article S1 Methods details

10.7717/peerj.1228/supp-5 Article S2 Summary of MMLE theory
