# Supplementary material for: An explanation of the relationship between mass, metabolic rate and characteristic length for placental mammals
Source: PeerJ. 2015 Sep 3;3:e1228. doi: 10.7717/peerj.1228 (PMC4562252; doi:10.7717/peerj.1228)
Supplement: Article S1 [file peerj-03-1228-s004.docx]

**Article S1. Methods Details.**

**Methods.** Determining numerical values for the universal constants G_m_, G_o_ and G_r_ requires three equations. Since equation (3) is just a version of equation (1) another equation is required. Numerical values for the constants G_m_ and G_o_ could be obtained by solving equation (3) for two different samples of body mass and characteristic length, l. MMLE uses a more general approach.

An approximation to equations (1) and (3) is:

W = dl^x^ (4)

This expression is useful for establishing the approximate relationship between body mass, W, and characteristic length, l, by regression analysis of W, l data for a group of animals. For least squares regression a figure of merit for how well the expression represents the data is the coefficient of determination, R^2^. The coefficient of determination measures the fraction of the dependent variable’s variance that is explained by the expression.

Least squares regression is performed on the logarithmic version of equation (4), log(W) = log(d) + xlog(l), to obtain best estimate values for log(d) and x.

Another useful expression is obtained by equating the derivatives with respect to log(l) of the logarithms of equations (3) and (4) to obtain, for s = 1.0 and geometrically similar non-skeletal musculature where y = 2/3, an equation for the exponent x in equation (4):

x = 2.0 +r + (1-r)(G_o_/e)^3/2^/(l^(r-1)^G_m_/kec + (G_o_/e)^3/2^) (5)

Equation (5) is the third equation needed to establish numerical values for the universal constants G_m_, G_o_ and G_r_.

The usual way to get the best estimate value for the parameter x in equations like equation (4) has been by regression analysis of the logarithmic version of the equation. The analyses have used species-averages in which a datum is the average value for a collection of individuals from a single species. In ordinary least squares (OLS) and reduced major axis (RMA) regression analyses it has been assumed that the species-averaged data are statistically independent. Phylogenetically informed (PI) analyses assume that the data are not independent but covary with the degree of phylogenetic relatedness between species. These regression methods are reviewed in detail by (White & Kearney, 2014).

By equations (2) and (3) MMLE deterministically predicts the absolute value of BMR and body mass for individual samples rather than a statistical best fit average value for a collection of individuals from a single species. MMLE is thus compatible with data sets that contain individual animal data. It is also compatible with species-averaged data sets by considering each species-averaged datum to correspond with at least one individual member of the species

**______________________________________________________________________________**

**
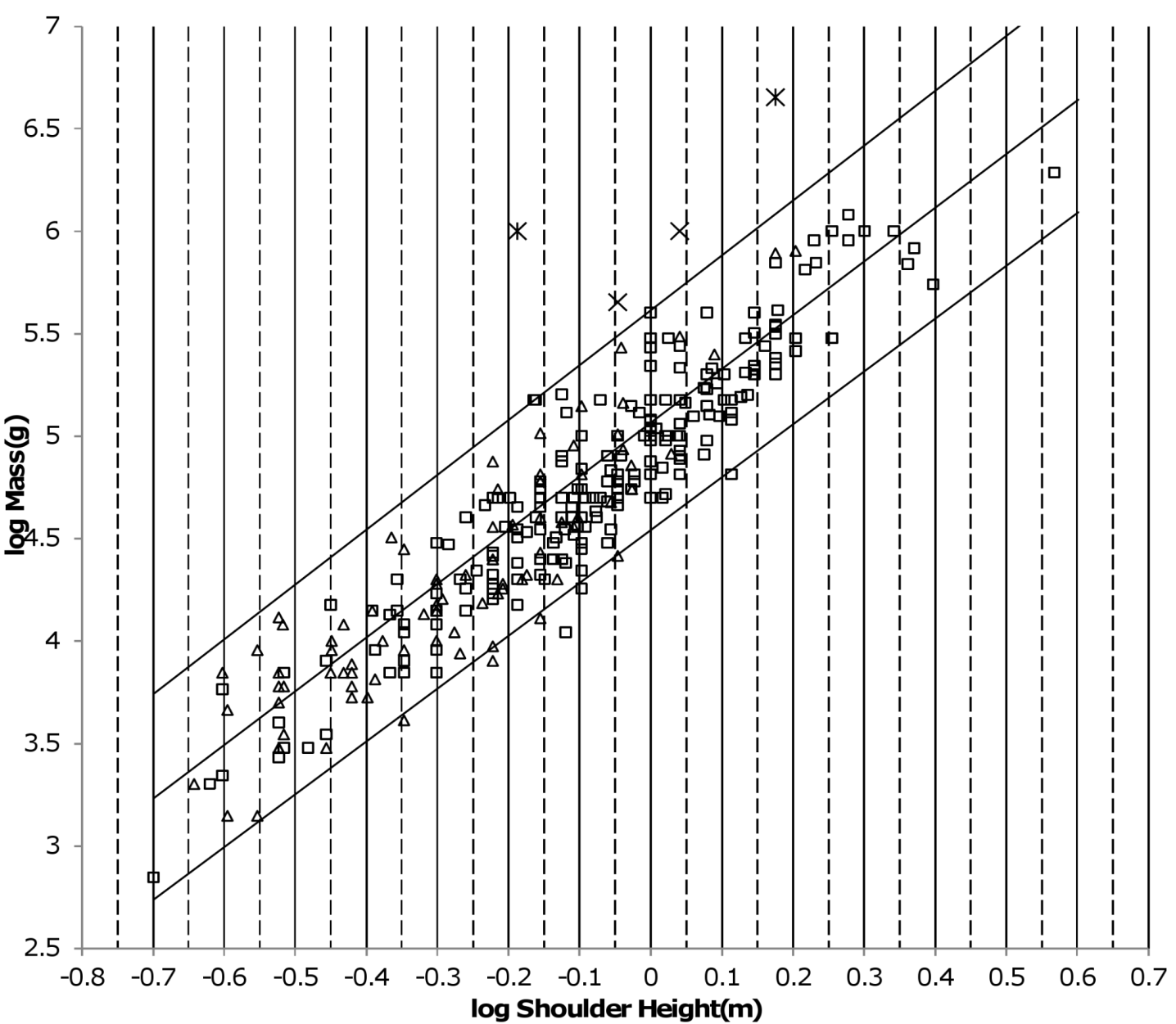
**

**Figure 1. Log body mass as a function of log shoulder height for running/walking Artiodactyla and Carnivora.** Data are from (Nowak, 1999). The upper and lower slanted solid lines are MMLE sturdiness factor boundaries for y = 2/3. The upper boundary was generated with a sturdiness factor, s, of the square root of 3, (3)^0.5^. The lower boundary was generated with s = (3)^-0.5^. The middle slanted line was generated with s = 1.0. The slanted lines are for Froude-Strouhal dynamic similarity. The Artiodactyla mass and shoulder height data are marked by open squares. The Carnivora mass and shoulder height data are marked by open triangles. Excluding *Hippopatamus amphibus* marked by crossed Xes and domestic cattle marked by Xes, R_M_^2^ = 0.9997. The solid vertical lines demark the AVG method first set of cohorts. The dashed vertical lines demark the second set of cohorts.

If a group of animals have nearly identical values for all the parameters occurring in equation (2) and (3) except for characteristic length, l, and sturdiness factor, s, then BMR and body mass can be regressed on characteristic length with sturdiness factor boundaries as illustrated in Fig. 1. As long as the only parameters in equations (2) and (3) that distinguish one individual animal from another are characteristic length and sturdiness factor the individuals included in the analysis may be from different species, genera, families, and even orders. Such a group of animals are called a MMLE phylogenetic homogeneous group herein.

The AVG regression method exploits this feature of MMLE to use individual animal data such as the unmodified data from (Nowak, 1999).

AVG regression is best explained with reference to Fig. 1. The appendix to the original paper shows that the OLS regression relationship between body mass and characteristic length is given by the equivalent of equation (3) evaluated with a sturdiness factor value of s = 1.0 for a MMLE phylogenetic homogeneous group. The s = 1.0 regression relationship is plotted by the middle slanting line in Fig. 1. For any particular characteristic length, the mean of the logs of all body masses with that characteristic length that are uniformly distributed between the upper and lower sturdiness factor boundaries is the log of the body mass predicted by equation (3) evaluated with a sturdiness factor of s = 1.0. MMLE takes advantage of this property to get linear regression relationships between log body mass regressed on log characteristic length with coefficients of determination, R^2^, that are very nearly 1.0.

The method is to group the body mass and characteristic length data into adjacent but non-overlapping cohorts. Each cohort is bounded by a lower and upper value of characteristic length. For the running/walking Artiodactyl and Carnivore data of Fig. 1, a set of 13 cohorts was selected so that they would be evenly spaced on the log characteristic length axis. The solid vertical lines in Fig. 1 show the boundaries of the cohorts. Within each cohort the characteristic lengths for the data contained in the cohort was linearly averaged to get a single value for the cohort. The body masses contained in the cohort were also linearly averaged to get a single value for the cohort. As a check for reasonableness a second set of cohorts was used. Each of these second cohorts overlapped the lower and upper length halves of adjacent cohorts from the first set of cohorts. The dashed vertical lines in Fig. 1 show the boundaries of the second cohorts. Because of the overlapping there are 14 second cohorts in Fig. 1. The mass and length data in the second cohorts was treated in the same way as it was in the first cohorts. An OLS linear regression was then performed using the logarithms of the cohort average mass and average length as data for both the first and second cohort sets. The regression relationship that was obtained for the Fig. 1 first cohort set was log(W) = 2.4861log(l) + 5.03 with R^2^ = 0.9911. The relationship obtained for the second set was log(W) = 2.6076log(l) + 5.01 with R^2^ = 0.9784.

The slopes of the two relationships differed significantly. This indicated that the slopes of the regression relationships were not very reasonable estimates of the exponent x in equations (4) and (5). A look at Fig. 1 indicates why the slopes could be so different. The average number of samples per first cohort is 23.7. The lowest characteristic length first cohort only contains six samples. The two upper most characteristic length first cohorts only contain one sample. Combining these sparsely populated first cohorts with their next adjacent cohort results in a first cohort regression relationship of log(W) = 2.6207log(l) + 5.0557 with R^2^ = 0.9932. For the second set of cohorts, the lowest characteristic length cohort only contains one sample. The three upper most characteristic length cohorts only contain four samples. Combining these sparsely populated second cohorts with their next adjacent cohort results in a second cohort regression relationship of log(W) = 2.604log(l) + 5.0611 with R^2^ = 0.9933. The slopes of these relationships are much closer than are the slopes of the previous relationships and a greater fraction of the variances are explained. Performing a OLS linear regression using both the first and second cohort data as a way of averaging the two cohort sets yields log(W) = 2.6112log(l) + 5.0584 with R^2^ = 0.9932. This regression of the combined cohort data is arbitrarily designated an “AVG” regression in the present paper. The slope of this AVG relationship was taken to be a good estimate of the exponent x in equations (4) and (5).

Combining sparsely populated cohorts into a cohort with a population that more closely approaches the average cohort population density finds some justification in the MMLE proposition that sturdiness factor is uniformly distributed between upper and lower sturdiness factor boundaries. A cohort needs a sufficient number of samples so that its mean is most likely close to the theoretical mean that would be obtained with a very large number of samples.

The next section describes using the AVG regression method on characteristic length, body mass data for running/walking placental mammals in the families Artiodactyla and Carnivora from (Nowak, 1999) to estimate numerical values for the parameter x in equations (4) and (5) and then the parameters G_m_/k and G_o_ in equations (1) and (3). The numerical values for G_m_/k and G_o_ apply to all placental mammals.

BMR and characteristic length data to estimate a numerical value for the parameter G_r_ in equation (2) was unavailable. Species-averaged BMR and body mass data was available from (Kolokotrones et al, 2010).

By equations (2) and (4),

BMR = G_r_(W/d)^2/x^ (6)

Since d and x are estimated by regression analysis this expression is reliable only if the coefficient of determination, R^2^, is nearly unity for the analysis that estimated d and x. From table 1 in the next section R^2^ = 0.9932 for the AVG regression analysis that estimated d and x for running/walking placental mammals in the families Artiodactyla and Carnivora. Although there are complications that will be discussed in the next section a reasonable estimate for the numerical value of G_r_ is obtained.

To solve for a numerical value of G_r_ an allometric expression of the form BMR = aW^b^ is obtained by regression analysis. If equation (6) is reliable then b = 2/x and a = G_r_/d^2/x^.

Phylogenetically Informed (PI) Generalized Least Squares regression was used to obtain the needed BMR = aW^b^ expression. The PI Generalized Least Squares regression analyses were conducted using the BayesTraits computer program (Pagel, Meade & Barker, 2004). PI methods are used to control for an assumed lack of statistical independence among species (Freckleton, Harvey & Pagel, 2002; White & Kearney, 2014).

The Microsoft Windows version of BayesTraits together with the companion programs BayesTrees and BayesTreesConverter available at [www.evolution.rdg.ac.uk](http://www.evolution.rdg.ac.uk) were used.

BayesTraits requires phylogenetic trees. For all mammals except Carnivora the tree for all mammals was used that is available as supplemental information from the online version of (Fritz, Bininda-Emonds & Purvis, 2009) at <http://onlinelibrary.wiley.com/doi/10.1111/j.1461-0248.2009.01307.x/suppinfo> .This tree contains 5020 species of mammals. For Carnivora an updated tree was used that is available from the online version of (Nyakatura & Bininda-Emonds, 2013) at [www.ncbi.nlm.nih.gov/pmc/articles/PMC3307490/](http://www.ncbi.nlm.nih.gov/pmc/articles/PMC3307490/) . This tree contains 286 Carnivore species. The trees were converted to nexus format input for BayesTraits using BayesTrees Converter. They were then pruned to the species that were contained in the data sets that were being analyzed using BayesTrees and manual editing of the nexus files. For running/walking mammals the updated pruned Carnivora trees were grafted to the older trees that had been pruned to the Artiodactyla, Proboscidea and Perissodactyla species that were contained in the data sets. Polytomies were automatically resolved using the BayesTrees resolve trees command with a branch length of 0.01 million years.

The PI regression analyses were performed with the BayesTraits continuous regression model and the maximum likelihood analysis type. The inputs were set to estimate the parameter lambda.

Lambda is found by maximum likelihood. It usually varies between 0.0 and 1.0. It is a multiplier of the off-diagonal elements of the Generalized Least Squares variance-covariance matrix. Lambda = 0.0 indicates evolution of traits that is independent of phylogeny, while lambda = 1.0 indicates that traits are evolving according to Brownian motion. Intermediate values indicate that traits have evolved according to a process in which the effect of phylogeny is weaker than in the Brownian model (Pagel, 1999; Freckleton, Harvey & Pagel, 2002; Pagel, Meade & Barker, 2004; Capellini, Venditti & Barton, 2010). BayesTraits results generated with lambda = 0.0 are the same as those obtained with Ordinary Least Squares (OLS) linear regression. Results generated with lambda = 1.0 are the same as those generated by phylogenetically independent contrasts (Capellini, Venditti & Barton, 2010). Occasionally lambda was estimated to be greater than 1.0. This can be interpreted as traits that are more similar than what is predicted by Brownian motion (Freckleton, Harvey & Pagel, 2002).

Since BayesTraits estimates maximum likelihood for a hypothesis (such as the applicable value of lambda), the log likelihood ratio for two hypotheses can be computed. By convention a value of 4.0 or greater for the ratio is taken as evidence that one of the hypotheses explains the data significantly better than the other (Pagel, 1999).

PI regression analyses were performed for both BMR regressed on body mass and body mass regressed on characteristic length. The species-averaged BMR, body mass data from (Kolokotrones et al, 2010) was inputted directly to BayesTraits. The body mass, characteristic length data from (Nowak, 1999) are mostly maximum and minimum measurements of individuals from the same species or genera in which a datum is the measurements for the largest or smallest individual measured in the taxon. Body mass, characteristic length data were taxon-averaged before being inputted to BayesTraits.

Together with the dynamic similarity implied by the mode of locomotion for a group of animals, PI regression relationships are helpful for partitioning populations into MMLE phylogenetic homogeneous groups to which the AVG regression technique can be applied as when a geometrically similar partition can be identified by a log(mass) regressed on log(length) slope of 3.0 or a log(BMR) regressed on log(mass) slope of 2/3. Since the only parameters in equation (3) that differ between individual members of a MMLE phylogenetic homogeneous group are the characteristic length, l, and the sturdiness factor, s, further consideration of the relatedness of members should not be necessary.

Regression analysis measures the error for a datum as the distance between the datum and a line established by the regression analysis. The line minimizes the sum of the squares of the errors. Using Fig. 1 as an example, the usual way of measuring error for a datum is the vertical distance from the datum to the middle slanting line given by equation (3) evaluated with a sturdiness factor s = 1.0. The coefficient of determination, R^2^, equals 1.0 – (sum of the squares of the errors)/(variance of the dependent variable). R^2^ is interpreted as the fraction of the dependent variable variance that is explained by the regression line (Edwards, 1984). The dependent variable in Fig. 1 is body mass.

In MMLE theory the OLS regression line can be replaced by a ‘band’. The band is the area enclosed by the sturdiness factor boundaries. Recognizing this fact, R^2^ can be replaced by an MMLE version that is denoted “R_M_^2^”. R_M_^2^ is computed in the same ways as R^2^ except that the ‘error’ for a datum that falls between the sturdiness factor boundaries is zero and the ‘error’ for a datum that that falls outside the sturdiness factor boundaries is the distance between the datum and the nearest sturdiness factor boundary. R_M_^2^ = 0.9997 for the data in Fig. 1.

R_M_^2^ is very nearly unity because the data considered in the present paper mostly lie between the sturdiness factor boundaries or very near to a boundary. Coverage, R, which is the fraction of the data that lies between the boundaries, is another figure of merit that is usually smaller than R_M_^2^.

Equation (3) evaluated with correct sturdiness factors exactly predicts every datum obtained from (Nowak, 1999) that is shown in Fig. 1. There is no error and thus no unexplained variance. R_M_^2^ is interpreted as a measure of how well data clusters within the sturdiness factor boundaries. R_M_^2^ is useful for estimating values for the parameters that appear in equation (3) when they may differ from the values established for running/walking placental mammals.

By examining individual animal metabolic rates and masses, (Hudson, Isaac &, Reuman, 2013) recently reported substantial metabolic rate heterogeneity at the species level and commented that this is a fact that cannot be revealed by species-averaged data sets. It was further commented that individual data might be more important than species-averaged data in determining the outcome of ecological interactions and hence selection. Heterogeneity is predicted by equations (2) and (3) due to variation of characteristic length and sturdiness factor among the individuals composing a species. The AVG regression method is compatible with species level heterogeneity.

Sturdiness factor boundaries and R_M_^2^ can also apply to the BMR, body mass data from (Kolokotrones et al, 2010). The boundaries are calculated by using equation (3) with the bounding sturdiness factors to calculate the body mass and using equation (2) with the bounding sturdiness factors to calculate the BMR for characteristic lengths that span the range of interest. The associated value of R_M_^2^ can then computed using the BMR as a function of body mass boundaries.

Equations (2) and (3) evaluated with a correct sturdiness factor and characteristic length exactly predicts every BMR and body mass datum used in the present paper that was obtained from (Kolokotrones et al, 2010). There is no error and thus no unexplained variance.

As will be discussed later the ability of equation (3) to exactly predict every datum extracted from one data set and its ability, when combined with equation (2), to exactly predict every datum extracted from another data set does not necessarily mean that there is no unexplained variance with MMLE theory.

The calculated numerical values reported in the present paper are given with four significant digits to the right of the decimal point. It is suspected that the data used is not accurate enough to support this precision.

**Running/Walking Placental Mammals.** The original paper established the numerical values for the constants in the MMLE equations by analysis of the running/walking members of the orders Artiodactyla, Carnivora, Perrisodactyla and Proboscidea. The four orders were analyzed together because their mode of locomotion was similar and it was expected that they would be dynamically similar so that the fundamental frequency of propulsion in equation (3) would scale similarly. Dynamic similarity rather than genetic similarity was considered more controlling of the relationship between mass and characteristic length in equation (3) because these orders are terrestrial runners/walkers that have evolved together in a predator/prey arms race. The genomes among these orders that have survived are the ones that have produced dynamically similar phenotypes suited to survival in terrestrial environments. The Artiodactyla are genetically more similar to the aquatic swimming Cetacea than they are to the other terrestrial running/walking orders (Bininda-Emonds, 2007) but their manner of locomotion is dynamically more similar to the other runners/walkers than it is to the swimming Cetacea. The Carnivora are more genetically similar to the aquatic swimming Pinnepedia than they are to the other terrestrial running/walking orders (Nayakatira, 2012) but their manner of locomotion is also more dynamically similar to the other runners/walkers than it is to the swimming Pinnepedia.

The fundamental frequency of propulsion for these terrestrial runners/walkers was established as the pendulum frequency obtained when both Froude and Strouhal dynamic similarity apply simultaneously. Limb length was considered to be the characteristic length that establishes the pendulum frequency. ( Raichlen, Pontzer & Shapiro, 2013) find that hip joint to limb center of mass is a better length for establishing the pendulum frequency. However little limb length or hip joint to limb center of mass data was available. Shoulder height data was more available, so shoulder height was adopted as an approximation of the characteristic length, l. 163 individual shoulder height, body mass samples were obtained from (Walker, 1968). The data was analyzed using the AVG regression method. The result was the relationship log mass = 2.66 log(shoulder height) + 2.17 with a correlation coefficient = 0.993. Thus the slope, x, in equation (5) was 2.66. Body mass was about 75 kg at the logarithmic mean shoulder height of the data of 0.782m. These values were used in the equivalent of equations (3) and (5) with unity sturdiness factor and unity mitochondrion capability quotient to simultaneously solve for the constants G_m_/k and G_o_ that occur in the equations. The results were G_m_/k = 295000 g/m^2^ sec and G_o_ = 1353 g^0.667^/m^2^ in the units used in the present paper. By examining the graph of body mass as a function shoulder height for the 163 samples, it was estimated that mass as a function of shoulder height lines computed from equation (3) with sturdiness factor values of the square root of 3, (3)^0.5^, and its inverse, (3)^-0.5^, approximately bounded the data.

BMR was predicted to scale as body mass raised to the 2/x power. Since x = 2.66 this meant that BMR scaled as body mass raised to the 0.75 power in agreement with Kleiber’s law. Kleiber’s law with additional data from (Economos, 1982) was used to estimate the constant G_r_ in equation (2) to be 142 watts/m^2^.

An expanded version of the methodology in the original paper was used to determine the constants in equations (1), (2) and (3) in the present paper. AVG and PI regressions of the new body mass on shoulder height data were used to determine the exponent in equation (4). This regression relationship was then to be used to determined body mass at a ’middle’ shoulder height. Then equations (3) and (5) were solved simultaneously for the constants G_m_/k and G_o_ occurring in the equations using unity sturdiness factor and unity mitochondrion capability quotient. The method for determining the constant G_r_ in equation (2) was more complicated.

226 individual and 129 species-averaged mass and shoulder height samples were obtained from (Nowak, 1999) for Artiodactyla (Data S1). *Hippopotamus amphibius* and *Bos Taurus* (aurochs and domestic cattle) were excluded from the analysis. *H. amphibious* was excluded because it is amphibious with significant adaptations for an aquatic existence. Aurochs were excluded because they are extinct. Domestic cattle were excluded because they have been bred for human utility rather than survival in nature. Figure 1 shows that both species are outliers to the main sequence of mass and shoulder height data for Artiodactlya. With these exceptions, the data spans the Artiodactyla range of mass and shoulder height from a small Asiatic mouse deer (*Tragulus javincus*) with a mass of 700g and shoulder height of 0.2m to a large giraffe (*Giraffa camelopardalis*)with a mass of 1930000g and a shoulder height of 3.7m.

84 individual and 43 species-averaged mass and shoulder height samples were obtained from (Nowak, 1999) for Carnivora (Data S1). The Mustelidae were mostly excluded because shoulder height was not given except for wolverines (*Gulo gulo*) and honey badgers (*Mellivoracapensis*). Shoulder height was not given for most of the smaller Carnivora so that the smallest sample was a raccoon (*Procyon*) with a mass of 2000g and a shoulder height of 0.228m. Thus while nearly three orders of magnitude of mass from the small raccoon to a large polar bear (*Urus martimus*) at 800000g are included in the data, almost two orders of magnitude of mass from a small least weasel (*Mustela nivalis*) at 30 g to the small raccoon were not available.

26 individual and 14 species-averaged mass and shoulder height samples were obtained from (Nowak, 1999) for Perissodactyla. Eight individual and three species-averaged samples were obtained for Proboscidea (Data S1).

Table 1 shows the AVG and PI regression analysis results obtained with this data. For mass regressed on shoulder height, there were too few samples of Proboscidea to perform a meaningful PI regression analysis and the AVG first and second cohort sets did not converge. The PI slope and intercept for Perissodactyla differed significantly from those for Carnivora and Artiodactyla and the AVG first and second cohort sets did not converge. However the slope and intercept for Carnivora and Artiodactyla were not significantly different. They were also not significantly different from the slope and intercept obtained from PI regression analysis of the combination of Carnivora and Artiodactyla and the AVG first and second cohort sets did converge. This strengthened the conjecture that Carnivora and Artiodactyla were dynamically similar with a fundamental propulsion frequency in equation (3) that scales similarly with characteristic length. For these reasons Carnivora and Artiodactyla mass and shoulder height were analyzed together and Proboscidea and Prissodactyla were considered separately.

From the Artiodactyla + Carnivora AVG mass on shoulder height regression slope the exponent, x, for equation (4) is 2.61.Since total body mass scales with an exponent, 2.61, that is greater than the exponent with which the skeletal muscle mass scales, 2.5, the non-skeletal muscle mass must scale with an exponent greater than 2.61. The simplest assumption is geometric similarity so that the non-skeletal muscle mass scales with an exponent of 3.0. The corresponding value for y is 2/3. The shoulder height is 0.86m for the mid point of the Artiodactyla + Carnivora log(shoulder height) data. By the AVG regression relationship the associated body mass is 77155g. Simultaneously solving equations (4) and (5) with these values results in G_m_/k = 274000 g/m^2^ sec and G_o_ = 900 g^0.667^/m^2^.

The PI mass on shoulder height regression slope for Artiodactyla + Carnivora is not significantly different from 2.5 as the log likelihood ratio for the table 1 slope and 2.5 is less than 4.0 (Pagel, 1999). Since for Froude-Strouhal similarity the skeletal musculature scales with an exponent of 2.5, this implies that the non-skeletal musculature also scales with an exponent of 2.5 which means that y = 0.8 in equation (3). The equivalent of equation (5) with y = 0.8 and r = 0.5 only states that x = 2.5 and provides no information for estimating G_m_/k and G_o._ Additionally equation (3) must be used to estimate the dimensionality factor, m.

G_m_/k should not change if y changes. The shoulder height is 0.88m for the mid point of the Artiodactyla + Carnivora log( shoulder height) data. By the PI regression relationship the associated body mass is 86538g. Using the previously established values of G_m_/k and G_o_ with y = 0.8 in equation (3) evaluated at these shoulder height and body mass values results with a values for the dimensionality factor of m = 4.425 g^0.133^.

The new values for G_m_/k and G_o_ are less than the values computed in the original paper. The 163 samples in the original paper included two Probicidae and 11 Perissodactyla whereas the 310 samples in the present paper were entirely Artiodactyla or Carnivora.

Figure 2 shows the MMLE mass as a function of shoulder height sturdiness factor boundaries for simultaneous Froude-Strouhal dynamic similarity as predicted by equation (3) evaluated with the new values for G_m_/k and G_o_ for both y = 2/3 and for y = 0.8 with the corresponding values of the dimensionality factor, m. The upper boundary uses a sturdiness factor of (3)^0.5^. The lower boundary uses a sturdiness factor of (3)^-0.5^. The reference (Nowak, 1999) mass and shoulder height samples for Artiodactyla and Carnivora are also shown. The data spans this full range of sturdiness factor for both values of y. The boundaries are hardly distinguishable for the two y values.

**___________________________________________________________**

**Table 1.** Results of regression analyses for running/walking placental mammals.

|  | **Regression** | **Independent** | **Dependent** |  | | | **Number** |
| --- | --- | --- | --- | --- | --- | --- | --- |
| **Order or Family** | **Type** | **Variable** | **Variable** | **Slope** | **Intercept** | **R^2^** | **Samples** |
| Artiodactyla + Carnivora | AVG  PI(0.89) | Height(m)  Height(m) | Mass(g)  Mass(g) | 2.6112  2.4893 | 5.0584  5.076 | 0.9932  0.8435 | NA  172 |
| All | AVG | Height(m) | Mass(g) | 2.8711 | 5.1677 | 0.9886 | NA |
|  | PI(0.92) | Height(m) | Mass(g) | 2.4875 | 5.2517 | 0.8424 | 189 |
| Ruminant Artiodactyla | PI(0.0) | Mass(g) | BMR(watts) | 0.7805 | -1.8058 | 0.9742 | 19 |
| Carnivora | PI((0.87) | Mass(g) | BMR(watts) | 0.7579 | -1.8826 | 0.909 | 59 |
| Carnivora less Mustelidae | PI(0.61) | Mass(g) | BMR(watts) | 0.758 | -1.8937 | 0.9053 | 46 |
| Mustelidae less Enhydra | PI(1.0) | Mass(g) | BMR(watts) | 0.6852 | -1.4688 | 0.9653 | 12 |
| Perrisodactyla | PI(1.0) | Height(m) | Mass(g) | 1.784 | 5.4961 | 0.8046 | 14 |
|  |  |  |  |  |  |  |  |
|  |  |  |  |  |  |  |  |
|  |  |  |  |  |  |  |  |

The regression expressions are: Log(dependent variable) = slope X log(independent variable) + intercept. AVG means the cohort averaging regression method. PI(n) means the phylogenetic informed regression method using BayesTraits and the number in parentheses is the estimated value of lambda. “All” in the Order or Family column means the combination of Artiodactyla, Carnivora, Perissodactyla and Proboscidea. Height(m) is shoulder height in meters. Mass(g) is body mass in grams. BMR(watts) is basal metabolic rate in watts. NA means Not Applicable. __________________________________________________________

______________________________________________________________________________


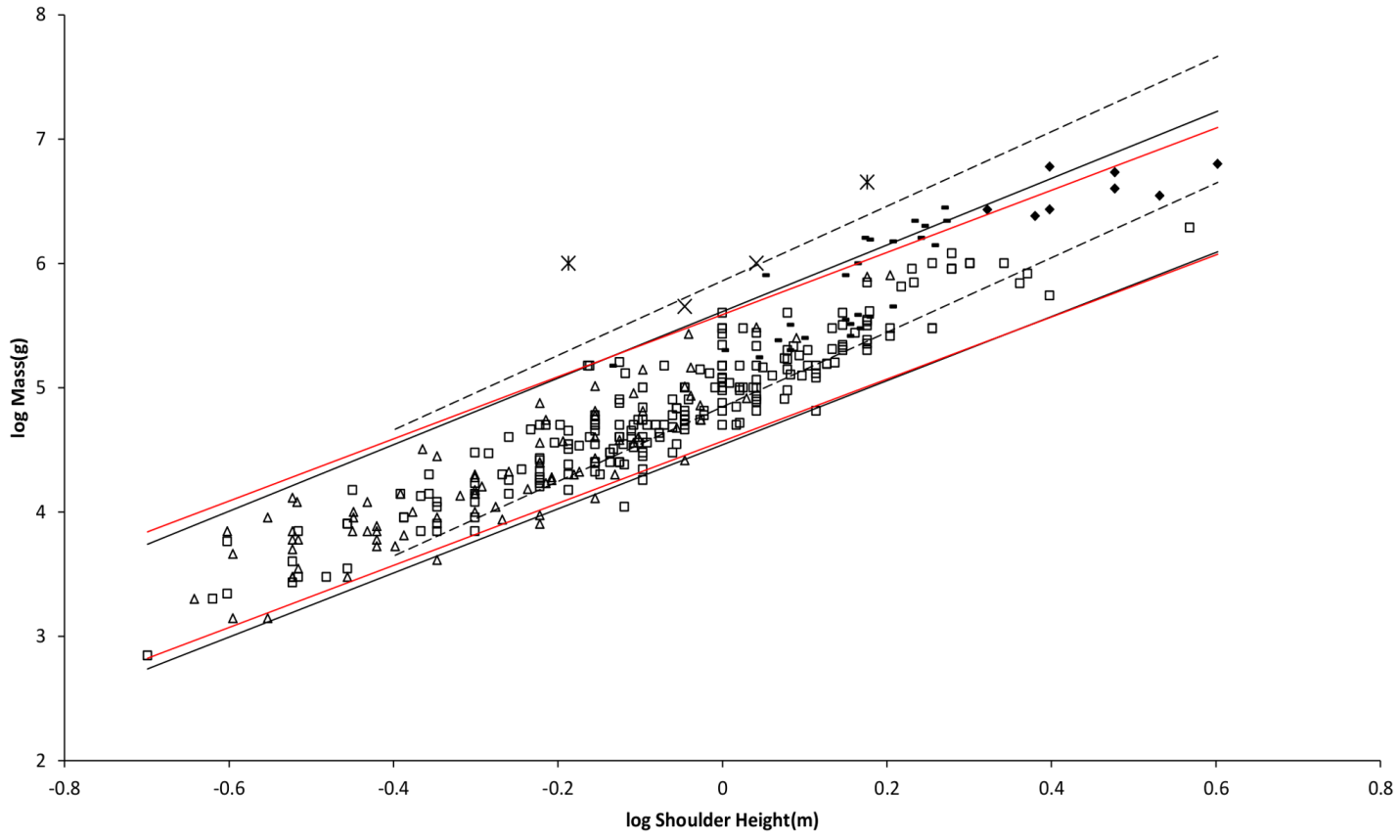


**Figure 2. Log body mass as a function of log shoulder height for running/walking placental mammals**. Data are from (Nowak, 1999). The solid and dashed lines are MMLE sturdiness factor boundaries. The upper boundaries were generated with a sturdiness factor s = (3)^0.5^. The lower boundaries were generated with s = (3)^-0.5^. The solid boundary lines are for Froude-Strouhal dynamic similarity. The black solid lines are for y = 2/3. The colored solid lines are for y = 0.8. The dashed boundary lines are for geometric similarity. The colored boundary lines, the geometric similarity boundary lines and the Perissodactyla and Proboscidea mass, shoulder height data have been added to the Artiodactyla and Carnivora data displayed in figure 1. For Artiodactyla and Carnivora R_M_^2^ = 0.9997 with respect to the solid black boundaries and R_M_^2^ = 0.9992 with respect to the colored boundaries. For Perissodactyla and Proboscidea R_M_^2^ = 1.0 with respect to the geometric similarity boundaries. Perissodactyla data are marked with solid rectangles. Proboscidea data are marked with solid diamonds. Aritiodactyla data are marked with open squares. Carnivora data are marked with open triangles. Crossed Xes mark *Hippopotamus amphibious*. Xes mark domestic cattle.

Both the y = 2/3 and the y = 0.8 sturdiness factor boundaries nearly embrace the combination of Artiodactyla, Carnivora, Perissodactyla and Proboscidea, or ‘all’ runners/walkers, in Fig. 2. A few Proboscidea and Perissiodactyla are outliers. From table 1 the PI regression of body mass on shoulder height indicates that all runners/walkers scale with the same slope as Artiodactyla + Carnivora, albeit with a greater intercept value that would require revision of the constants in equation (3). With a slope of 2.87 the AVG regressions of mass on shoulder height for all runners/walkers indicates a combination of Froude-Strouhal and geometric similarity. A geometrically similar fundamental frequency of 1.4 m/sec divided by shoulder height was heuristically found to embrace the Perissiodactyla and Proboscidea data as shown in Fig. 2. Other than the change to the fundamental frequency formulation, the MMLE geometric similarity sturdiness factor boundaries in Fig. 2 use the same constants that were used to generate the Froude-Strouhal boundaries for y = 2/3. The data very nearly span the full range of sturdiness factor between the boundaries. The relationship between mass and shoulder height for Perissodactyla and Proboscidea may be better explained by geometric similarity.

For running/walking placental mammals the reference (Kolokotrones et al, 2010) BMR and body mass species-averaged data set contained 20 samples for Artiodactyla and 58 samples for Carnivora.

There are doubts that ruminant Artiodactyla can meet the criteria for measuring BMR (McNab, 1997; White & Seymour, 2003). All but one of the Artiodactyla samples were ruminants. For these reasons the BMR and mass data for ruminant Artiodactyla and Carnivora were analyzed separately. Ruminants inability to achieve a post absorptive state should not affect the relationship between body mass and shoulder height so revisiting that analysis because of this issue is not needed.

There is a major composition difference between the mass and shoulder height data and the BMR and mass data for Carnivora. Mustelidae compose only about 1% of the mass and shoulder height data whereas they compose over 20% of the BMR and mass data. For this reason the Mustelidae data was separated from the rest of the Carnivora data as shown in table 1.

Since the coefficient of determination, R^2^, for the AVG Artiodactyla + Carnivora mass on shoulder height regression in table 1 is very nearly unity, equation (6) can be used. With the G_m_/k and G_o_ values just determined the result is log(BMR) = 0.7659log(W) – 3.8744 + log(G_r_). Equating this last expression to the Carnivora less Mustelidae BMR regressed on mass expression in table 1 results with Gr = 95.6W^-0.0079^ watts/m^2^. The mass residual is not significant as the log likelihood ratio for the exponent obtained with equation (6) and the exponent in Table 1 is 0.1 which is significantly less than 4.0 (Pagel, 1999).

The log(shoulder height) mid point of the Carnivora data is l = 0.65m. A carnivore with this shoulder height would have a body mass of 40800g by the PI Artiodactyla + Carnivora mass on shoulder height regression relationship from table 1. Its non-Mustelidae BMR would be 39.9 watts by the PI BMR on mass regression relationship from table1. From equation (2) G_r_ = 94.5 watts/m^2^.

A middle value of G_r_ = 95 watts/m^2^ was used to generate the MMLE sturdiness factor boundaries in Fig. 3. As discussed in the summary of the derivation of the MMLE equations, this value of G_r_ should be the basic value for all non-ruminant placental mammals.

Similarly, values for G_rR_ for ruminant Artiodactyla of 138 watts/m^2^ and 144 watts/m^2^ are obtained for the Artiodactyla log(shoulder height) mid point of 0.86m. These values for G_rR_ for ruminant Artiodactyla are within three percent of the value for G_r_ computed in the original paper for all running/walking placental mammals. G_rR_ = 138 watts/m^2^ was used to generated the MMLE sturdiness factor boundaries in Fig. 3.

The difference between the ruminant Artiodactyla and Carnivora less Mustelidae BMR regressed on mass slopes in Table 1 are not significant as the log likelihood ratio is 2.8 which is less than 4.0 (Pagel, 1999). However the difference between the intercepts is significant as the log likelihood ratio is greater than 4.0. The significantly different intercepts support separating ruminant Artiodactyla and Carnivora less Mustelidae for BMR analyses.

An increased mitochondrion capability quotient is not the reason that G_rR_ is greater than G_r_. By equation (3) a greater mitochondrion capability quotient would result in a less massive animal for the same characteristic length. Figure 2 shows that both Artiodactyla and Carnivora have similar masses at the same characteristic length; and their PI mass regressed on characteristic length slopes are not significantly different. The difference between G_rR_ and G_r_ is more likely the result of sustained digestive activity by ruminant Artiodactyla (McNab, 1997; White & Seymour, 2003).

Figure 3 shows the MMLE BMR as a function of body mass sturdiness factor boundaries for Froude-Strouhal scaling evaluated with the new values for G_r_ and G_rR_. The boundaries were generated by simultaneously using equations (2) and (3). The differences between the y = 2/3 and y = 0.8 curves are due to the different masses predicted by equation (3) for the same characteristic length with the different values of y. In terms of R_M_^2^ the differences are barely distinguishable. The upper boundaries use a sturdiness factor of (3)^0.5^. The lower boundaries use a sturdiness factor of (3)^-0.5^. The reference (Kolokotrones et al, 2010) BMR and body mass samples for Artiodactyla and Carnivora are also shown. The single Proboscid datum (Savage et al, 2004) is included for reference. Ruminant Artiodactyla do have a BMR that is elevated with respect to Carnivora of the same mass. The single non-ruminant Artiodactyla datum, a dromedary camel (*Camelus dromedaries*), is embraced by the non-Mustelidae Carnivora MMLE boundaries rather than the ruminant boundaries. The data more than spans the full range of sturdiness factor.

Also shown in Fig. 3 is the Mustelidae BMR and mass data. It is better embraced by the ruminant MMLE sturdiness factor boundaries, but Mustidae do not have the digestive features that are the probable source of the ruminants’ elevated BMR. The non-Mustelidae Carnivora value for G_r_ should apply to the Mustelidae also, but their MMLE boundaries hardly embrace any of the Mustelidae data. Sturdiness factor and characteristic length values could be found so that equations (2) and (3) would exactly predict each Mustelidae BMR and mass datum shown in Fig. 3, but the sturdiness factors would mostly be greater than the maximum needed to predict the BMR and mass for other Carnivore families. Other MMLE parameters can be adjusted so that the Mustelidae data could be bounded by the same sturdiness factor values that bound all the other running/walking placental mammal data.

The Mustelidae data contained one sample for a sea otter (*Enhydra lutris*) which is an ocean going swimmer rather than a runner/walker. Separating this sample from the rest of the Mustelidae data leads to a slope of 0.69 as shown in table 1. This slope is not significantly different from the geometric similarity slope of 0.67 as the log likelihood ratio for these slopes is 0.4 which is less than 4.0 (Pagel, 1999). Geometric rather than Froude-Strouhal similarity supports separating Mustelidae from the rest of Carnivora for BMR analyses. Equations (3) with y = 2/3 and a geometric similarity fundamental frequency of 1.4 (m/sec)/l and equation (2) are the MMLE equations for relating BMR to body mass for geometric similarity. Of the constant parameters in these equations, values applicable to Mustelidae for G_m_, G_o_, and G_r_ have been established. The MMLE parameters that could be adjusted to account for the Mustelidae BMR deviation are the fundamental propulsion frequency constant, c, the mitochondrion capability quotient, e and the dynamic similarity constant, k. Because many Mustelidae combine swimming with terrestrial locomotion it is possible that their skeletal musculature may not be dynamically similar to other Carnivora and the constant k may be different from a value of 1.0. However, by equation (3), k and c cannot be separated without additional information. The product kc and e were varied to obtain the Mustelidae geometric similarity MMLE sturdiness factor boundaries shown in Fig. 3. Holding constant the combined fundamental propulsion frequency and dynamic similarity constant at a value of kc = 1.4 m/sec as was used for the geometric similarity MMLE mass and shoulder height boundaries for Perissodactyla and Proboscidea required a mitochondrion capability quotient at least 170% of that applicable to other Carnivora. That a Mustelidae mitochondrion would be this much more powerful than other carnivora mitochondria is difficult to believe. Using a combined fundamental propulsion frequency and dynamic similarity constant twice as large as that used for Perissodactyla and Proboscidea reduced the required mitochondrion capability quotient to 130% of that for other Carnivora. An implication would be that more powerful mitochondria allow Mustelidae to move their limbs twice as fast as other placental mammals in performing routine locomotion tasks.

______________________________________________________________________________


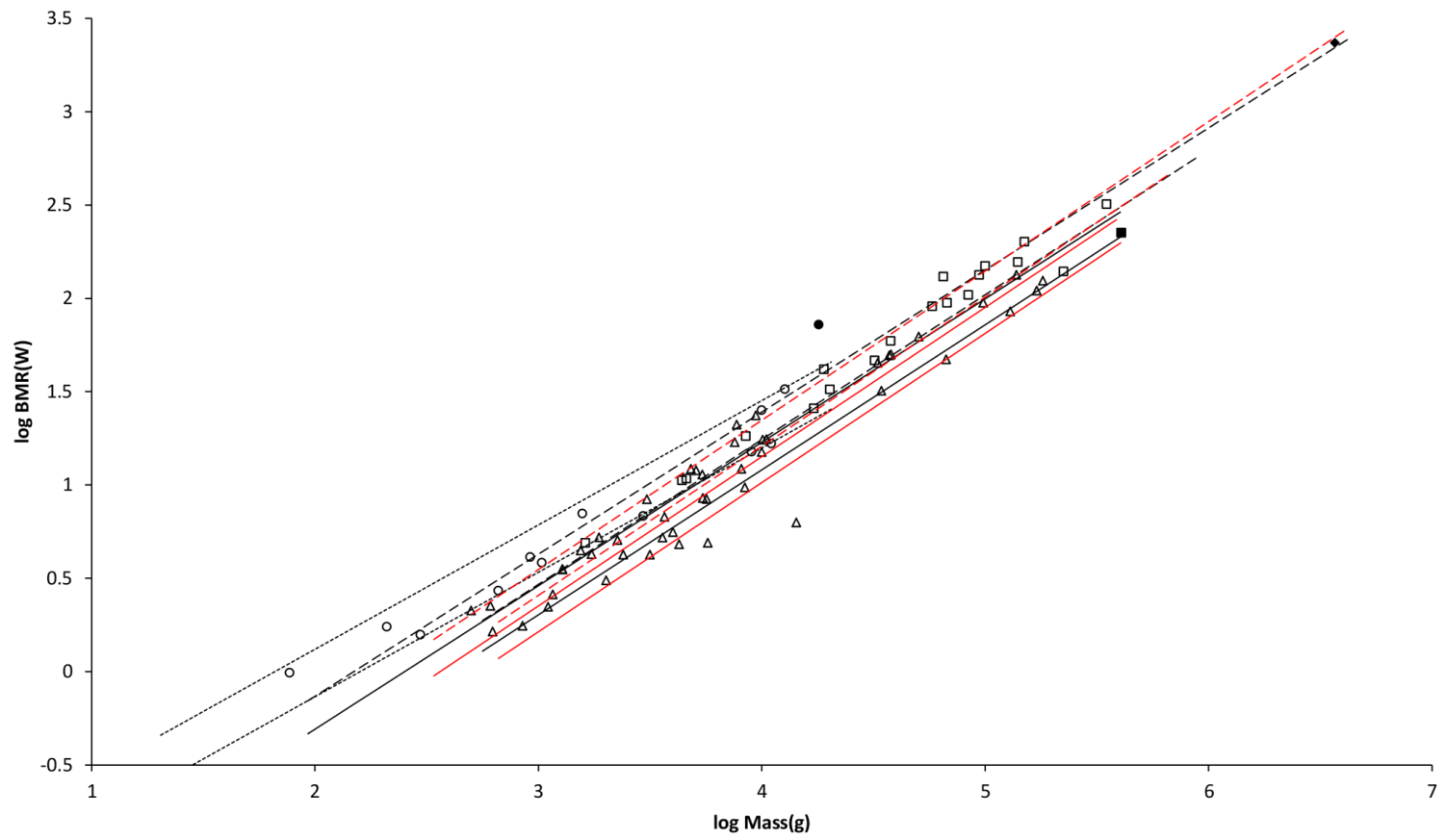


**Figure 3. Log BMR as a function of log body mass for running/walking placental mammals.** The *Elephas maximus* datum marked by a solid diamond is from (Savage et al, 2004). All other data are species-averages from (Kolokotrones et al, 2010).The solid, dashed and dotted lines are MMLE sturdiness factor boundaries. The upper boundaries were generated with a sturdiness factor s = (3)^0.5^. The lower boundaries were generated with s = (3)^-0.5^. The black lines are for y = 2/3. The colored lines are for y = 0.8. The steeper sloping boundary lines are for Froude-Strouhal dynamic similarity. The shallower sloping boundary lines are for geometric similarity. Ruminant Artiodactyla data are marked by open squares and R_M_^2^ is 0.9921 with respect to the dashed Froude-Strouhal black boundaries and R_M_^2^ is 0.9919 with respect to the dashed colored boundaries. *Camelius dromedarius* is a non-ruminant Artiodactyl marked by a solid square. Carnivora less Mustelidae data are marked with open triangles and R_M_^2^ is 0.9752 with respect to the solid Froude-Strouhal black boundaries and R_M_^2^ is 0.9655 with respect to the solid colored boundaries. Mustelidae except Enhydra data are marked with open circles and R_M_^2^ is 0.9999 with respect to the dotted geometric black boundaries. *Enhydra lutris* is an ocean going swimming Mustelid marked by a solid circle.

______________________________________________________________________________

Coverage, R, is the fraction of samples that fall between the MMLE sturdiness boundaries. Separating the Mustelidae from the rest of the Carnivora increased R for the entire Carnivora order from about 0.44 to 0.53 for y = 2/3 and from about 0.39 to 0.49 for y = 0.8. Although coverage is somewhat sparse, many of the samples lie close to the MMLE sturdiness boundaries as the greater R_M_^2^ values indicate.

MMLE can exactly predict every individual body mass and shoulder height datum from (Nowak, 1999) for running/walking placental mammals that is shown in Fig. 1 and 2. There is no error and thus no unexplained variance. Once the applicable skeletal muscle similarity is specified and values for the constants G_m_ and G_o_ are established, computing an individual animal’s mass from its shoulder height only requires specifying its sturdiness factor. Mustelidae are an exception. They require the further specification of a larger mitochondrion capability quotient, e, and an appropriate combined fundamental propulsion frequency and dynamic similarity constants, kc. As shown in Fig. 2 the differences are negligible between y = 2/3 which is indicated by the AVG mass on characteristic length regression relationship and y = 0.8 which is indicated by the PI mass on characteristic length regression relationship. The relationship between mass and characteristic length for Proboscidea and Perissiodactyla may be better explained by geometric similarity.

MMLE also can exactly predict every species-averaged BMR and mass datum from (Kolokotrones et al, 2010) for running/walking placental mammals that are shown in Fig. 3 by using equations (2) and (3) simultaneously. There is no error and thus no unexplained variance. Body mass is computed with equation (3) as just described. Whether an animal is a ruminant Artiodactyl or not needs to be specified to use the correct value of the constant G_r_ in equation (2) to compute the animal’s BMR from its shoulder height and sturdiness factor. Some iteration of this process may be required if the characteristic length and/or sturdiness factor is not included with the BMR and body mass data for the animal. As shown in Fig. 3 the differences in terms of R_M_^2^ are negligible between y = 2/3 and y = 0.8.

**Rodentia.** Rodentia comprise about 20% of families, 39% of genera, and 43% of species of recent mammals. Their masses range over four and a half orders of magnitude and their head and body lengths range over one and a half orders of magnitude (*Mus minutoides* with a mass of 2.5g and head and body length of 45mm to *Hydrochaeris hydrochaeris* with a mass of 79,000g and head and body length of 1,300mm). Various species employ scurrying, climbing, gliding, hopping, burrowing, swimming, running/walking , and combinations of these as their primary means of locomotion (Nowak, 1999).

(Nowak, 1999) provides individual data on approximate minimum and maximum masses in grams (g) and head and body lengths in millimeters (mm). The length data are mostly for genera with very little data for species. There is also very little data on shoulder height. Given the available data, linearly relating head and body length to characteristic length was tried. From the reference (Nowak, 1999), 203 individual and 105 taxon-averaged mass, head and body length samples were obtained (Data S2). (Kolokotrones et al, 2010) provides 267 species-averaged BMR in watts (W) and mass in grams (g) samples. Table 2 shows the PI and AVG regression analysis results obtained with this data. The length data was converted to meters (m) for calculations involving equations (1) through (5).

____________________________________________________

**Table 2.** Results of Regression Analyses for Rodentia.

|  | **Regression** | **Independent** | **Dependent** |  | | | **Number** |
| --- | --- | --- | --- | --- | --- | --- | --- |
| **Family** | **Type** | **Variable** | **Variable** | **Slope** | **Intercept** | **R^2^** | **Samples** |
| All Rodentia | PI(0.62) | Mass(g) | BMR(watts) | 0.7231 | -1.7198 | 0.8968 | 267 |
|  | PI(0.0) | Length(mm) | Mass(g) | 2.9482 | -4.3637 | 0.9571 | 105 |
|  | AVG | Length(mm) | Mass(g) | 2.8692 | -4.1497 | 0.9956 | NA |
| Non- | PI(0.44) | Mass(g) | BMR(watts) | 0.7399 | -1.7685 | 0.9192 | 176 |
| Cricetidae | PI(0.0) | Length(mm) | Mass(g) | 2.9079 | -4.2548 | 0.9571 | 78 |
|  | AVG | Length(mm) | Mass(g) | 2.8564 | -4.1124 | 0.9939 | NA |
| Cricetidae | PI(0.55) | Mass(g) | BMR(watts) | 0.6597 | -1.5408 | 0.8497 | 91 |
|  | PI(0.0) | Length(mm) | Mass(g) | 3.4061 | -5.3367 | 0.9395 | 27 |
|  | AVG | Length(mm) | Mass(g) | 2.9531 | -4.3561 | 0.9908 | NA |

The regression expressions are: Log(dependent variable) = slope X log(independent variable) + intercept. PI(n) means the phylogenetic informed regression method using BayesTraits and the number in parentheses is the estimated value of lambda. AVG means the cohort averaging regression method. Length(mm) is head and body length in millimeters. Mass(g) is body mass in grams. NA means Not Applicable

_______________________________________________________

Froude similarity should apply to skeletal muscle dynamics in most Rodentia as gravity is the main force affecting their locomotion. This should be true even for swimming as aquatic Rodentia are mainly surface swimmers that experience significant drag through the generation of surface waves in the wake. Drag through the generation of surface waves in the wake is the classic situation to which Froude similarity applies. What should govern the dynamics of burrowing is not clear, but as will be seen Froude similarity seems to work. The mass regressed on head and body length slope for both PI and AVG regressions for all families of Rodentia trends toward the geometric slope of 3.0. The combination of all families except Cricetidae trend toward an intermediate slope between the Froude-Strouhal slope of around 2.55 for mammals the size of Rodentia and the geometric slope. Cricetidae have an AVG mass regressed on length slope nearer the geometric similarity slope and the PI slope exceeds geometric similarity. Cricetidae also have a PI BMR regressed on mass slope that is not significantly different than the slope for geometric similarity as the log likelihood ratio for the slopes is 4.0 (Pagel, 1999).

Besides appearing to be more geometrically similar, Cricetidae tend to have a higher BMR when compared to non-Cricetidae of the same mass. For these reasons Cricetidae were analyzed separate from all the other families of Rodentia.

The slope values for mass regressed on head and body mass in table 2 for both PI and AVG regressions are very different from the value of 2.5 obtained for y =0.8 for Artiodactyla + Carnivora. They indicate either geometric similarity with y = 2/3 or a mixture of geometric similarity and Froude-Strouhal similarity with y = 2/3. For non-Cricetidae, the PI regression slope is not significantly different from the AVG slope as the log likelihood ratio for the two slopes is less than 4.0 (Pagel, 1999). For these reasons y = 2/3 is used in equation (3) for Rodentia.

The characteristic length for Rodentia was assumed to be a constant fraction of head and body length. The fraction’s value was estimated by equating the combined Artiodactyla and Carnivora mass regressed on length expression to the all families of Rodentia mass regressed on head and body length for the range of lengths for Rodentia. This results with a fraction that is within the range 0.4 to 0.62 using the PI regression relationship and 0.43 to 0.61 using the AVG relationship. A value of 0.5 was considered to be a reasonable working estimate for this characteristic length scaling fraction.

The characteristic length scaling fraction adds an additional parameter to the number that MMLE uses to predict the absolute values of body mass and BMR. Its addition increases the total number of parameters to 11.

The fundamental locomotion frequency for geometric similarity is a constant divided by the characteristic length. The value of 1.4 m/sec for the constant that worked well for Perissodactyla and Proboscidea was also used as the constant for Rodentia.

Figure 4 shows the MMLE mass as a function of head and body length sturdiness factor boundaries for Froude-Strouhal dynamic similarity and geometric similarity evaluated with the same constants that were used for running/walking mammals and a characteristic length scaling factor of 0.5. The reference (Nowak, 1999) mass, head and body length samples for non-Cricetidae Rodentia are also shown.

Figure 5 shows the MMLE BMR as a function of body mass sturdiness factor boundaries for the two similarity regimes evaluated with the same constants that were used for non-Mustelidae Carnivora and a characteristic length scaling factor of 0.5. The (Kolokotrones et al, 2010) BMR and mass samples for non-Cricetidae Rodentia are also shown.

Cricetidae were analyzed separate from all the other families of Rodentia because they appear to be more geometrically similar, and they tend to have a higher BMR when compared to non-Cricetidae of the same mass.

______________________________________________________________________________


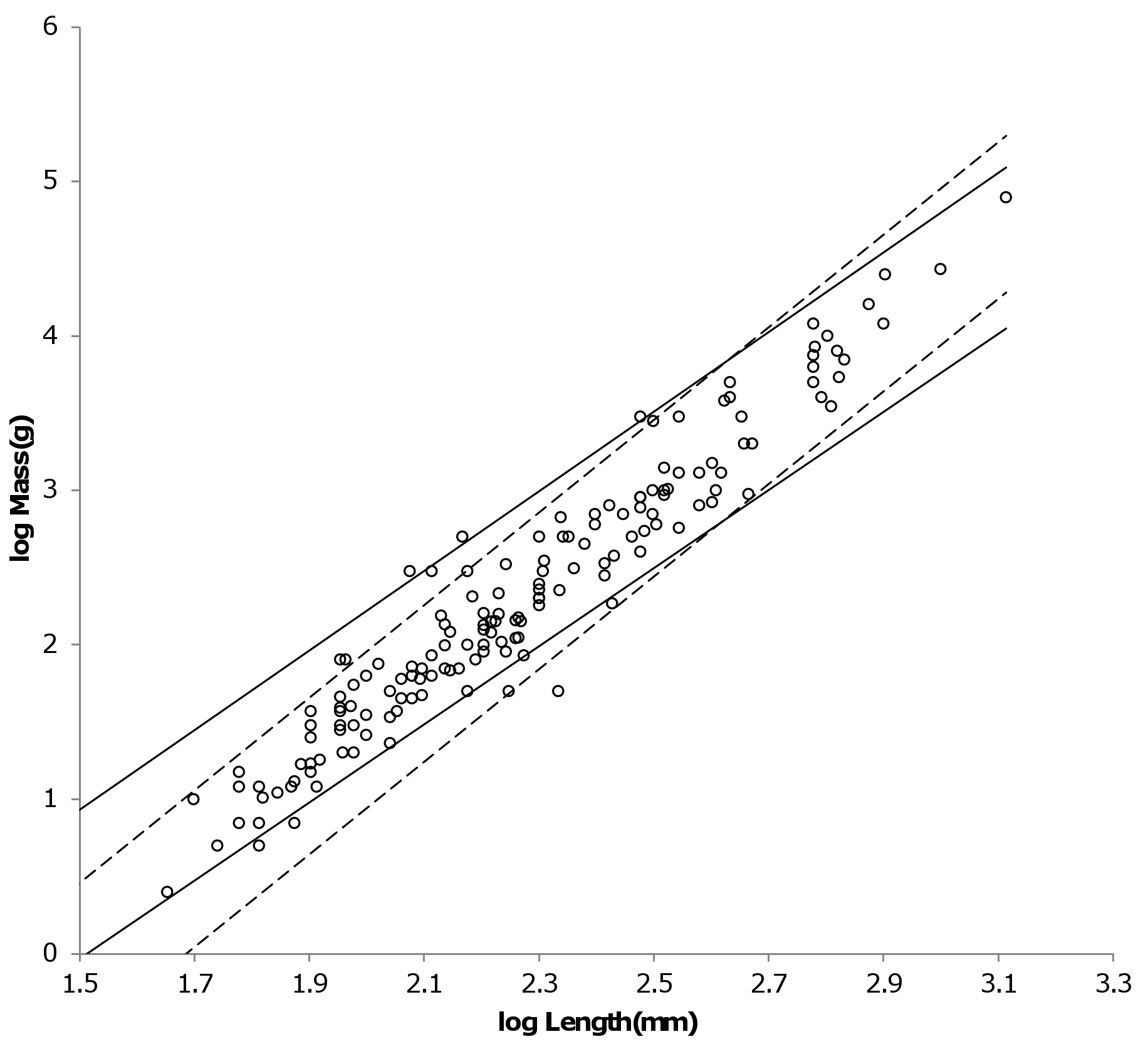


**Figure 4. Log body mass as a function of log head and body length for non-Cricetidae Rodentia.** Data are from (Nowak, 1999). The solid and dashed lines are MMLE sturdiness factor boundaries. The upper boundaries were generated with a sturdiness factor s = (3)^0.5^. The lower boundaries were generated with s = (3)^-0.5^. The shallower sloping solid boundary lines are for Froude-Strouhal similarity. The steeper sloping dashed boundary lines are for geometric similarity. The non-Cricetidae Rodentia individual mass, head and body length data are marked by open circles. R_M_^2^ = 0.9995 with respect to both sets of boundaries.

______________________________________________________________________________

______________________________________________________________________________


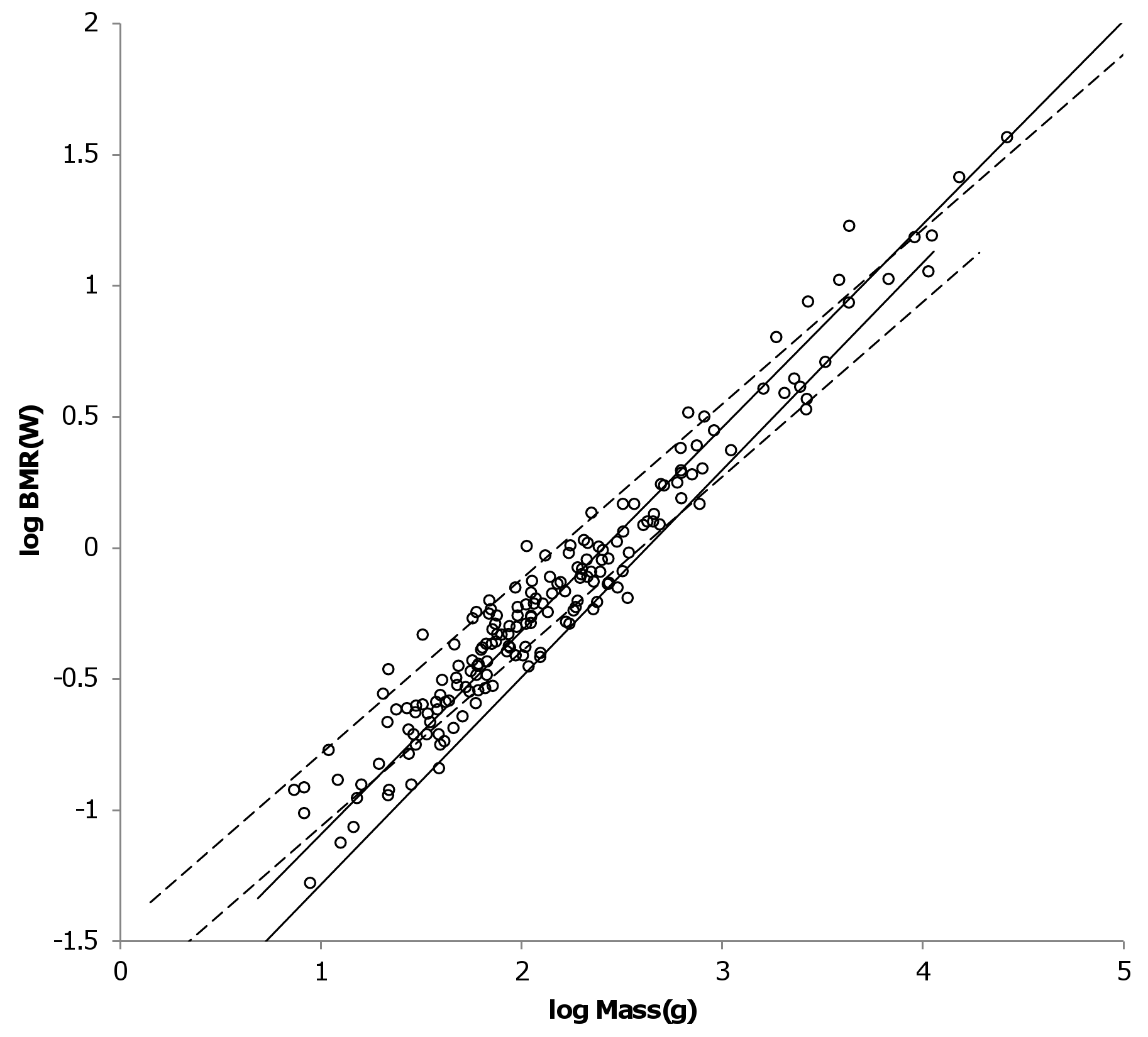


**Figure 5. Log BMR as a function of log body mass for non-Cricetidae Rodentia.** Data are from (Kolokotrones et al, 2010). The solid and dashed lines are MMLE sturdiness factor boundaries. The upper boundaries were generated with a sturdiness factor s = (3)^0.5^. The lower boundaries were generated with s = (3)^-0.5^. The steeper sloping solid boundary lines are for Froude-Strouhal similarity. The shallower sloping dashed boundary lines are for geometric similarity. The species-averaged non-Cricetidae Rodentia mass, head and body length data are marked by open circles. R_M_^2^ = 0.9966 with respect to both sets of boundaries.

As with Mustelidae that have a greater BMR at the same body mass than do other Carnivora, a greater mitochondrion capability quotient would most straight forwardly result in a greater BMR for Cricetidae with the same masses as other Rodentia. This possibility was evaluated for Cricetidae Rodentia. With all other parameters maintained identical to those used for geometrically similar non-Cricetidae Rodentia, the mitochondrion capability quotient was varied until a maximum value of R_M_^2^ was achieved for both mass as a function of length and BMR as a function of mass. A mitochondrion capability quotient of 1.2 maximized the R_M_^2^ values. Figure 6 shows the MMLE mass as a function of head and body length sturdiness factor boundaries for geometric similarity evaluated with this mitochondrion capability quotient value. The reference (Nowak, 1999) mass, head and body length samples for Cricetidae Rodentia are also shown. Figure 7 shows the MMLE BMR as a function of body mass sturdiness factor boundaries. The reference (Kolokotrones et al, 2010) BMR and mass samples for Cricetidae Rodentia are also shown.

Figure 6 shows that with the exception of a single outlier a tighter range of sturdiness factor for the MMLE boundaries could embrace the Cricetidae mass, head and body length data. The outlier is the larger sample of American harvest mice *(Reithrodontomys)*. However Fig. 7 shows that the full sturdiness factor range is needed to embrace the Cricetidae BMR and mass data.

A mitochondrion capability quotient of 1.2 as an explanation of why Cricetidae have an elevated BMR with respect to other Rodentia is considerably more palatable than the value of this parameter needed to explain the elevated BMR of Mustelidae with respect to other Carnivora.

MMLE can exactly predict every individual body mass, head and body length datum from (Nowak, 1999) for Rodentia that is shown in Fig. 4 and 6. There is no error and thus no unexplained variance. A characteristic length scaling fraction to translate the available head and body length data to characteristic length gives reasonable results. Then using the parameter values that were established for running/walking placental mammals, computing an individual non-Cricetid Rodent’s mass from its characteristic length with equation (3) only requires specifying its sturdiness factor and its skeletal muscle similarity. Calculating a Cricetid Rodent’s body mass from its characteristic length with equation (3) requires specifying its sturdiness factor, geometric similarity and a larger mitochondrion capability quotient than that used for other Rodentia and non-Musteletid running/walking placental mammals.

MMLE can exactly predict every BMR and body mass datum from (Kolokotrones et al, 2010) for Rodentia that is shown in Fig. 5 and 7 by using equations (2) and (3) simultaneously. There is no error and thus no unexplained variance. Body mass is computed with equation (3) as just described. Then the non- ruminant Artiodactyl value of the constant G_r_ established for running/walking placental mammals is used in equation (2) to compute the Rodent’s BMR from its shoulder height and sturdiness factor. Some iteration of this process may be required if the head and body length and/or sturdiness factor is not included with the BMR and body mass data for the animal.

______________________________________________________________________________


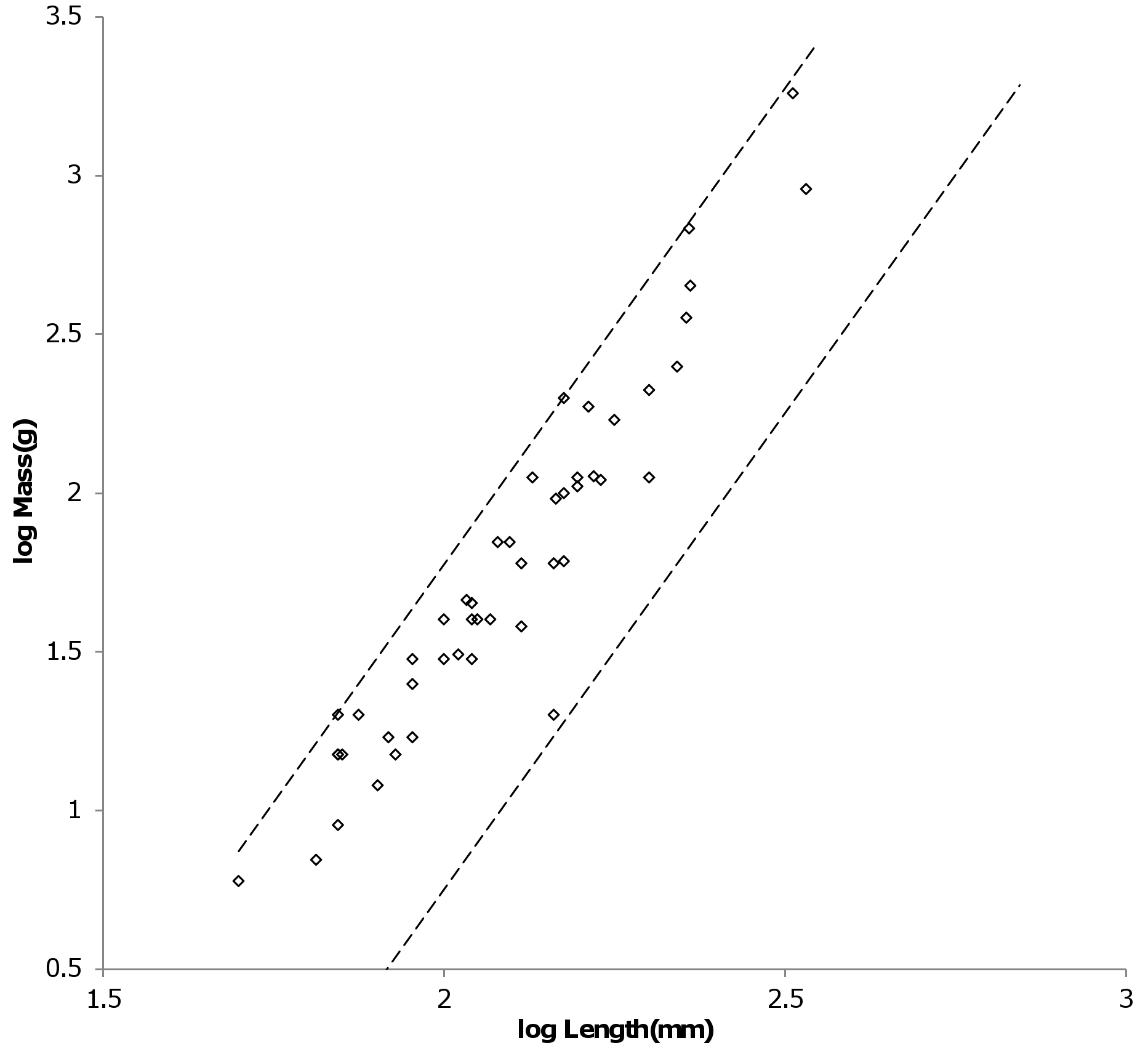


**Figure 6. Log body mass as a function of log head and body length for Cricetidae Rodentia.** The data are from (Nowak, 1999). The dashed lines are MMLE sturdiness factor boundaries. The upper boundary was generated with a sturdiness factor s = (3)^0.5^. The lower boundary was generated with s = (3)^-0.5^. The boundary lines are for geometric similarity. Cricetidae Rodentia individual mass, head and body length data are marked by open diamonds. R_M_^2^ = 1.0.

______________________________________________________________________________

______________________________________________________________________________


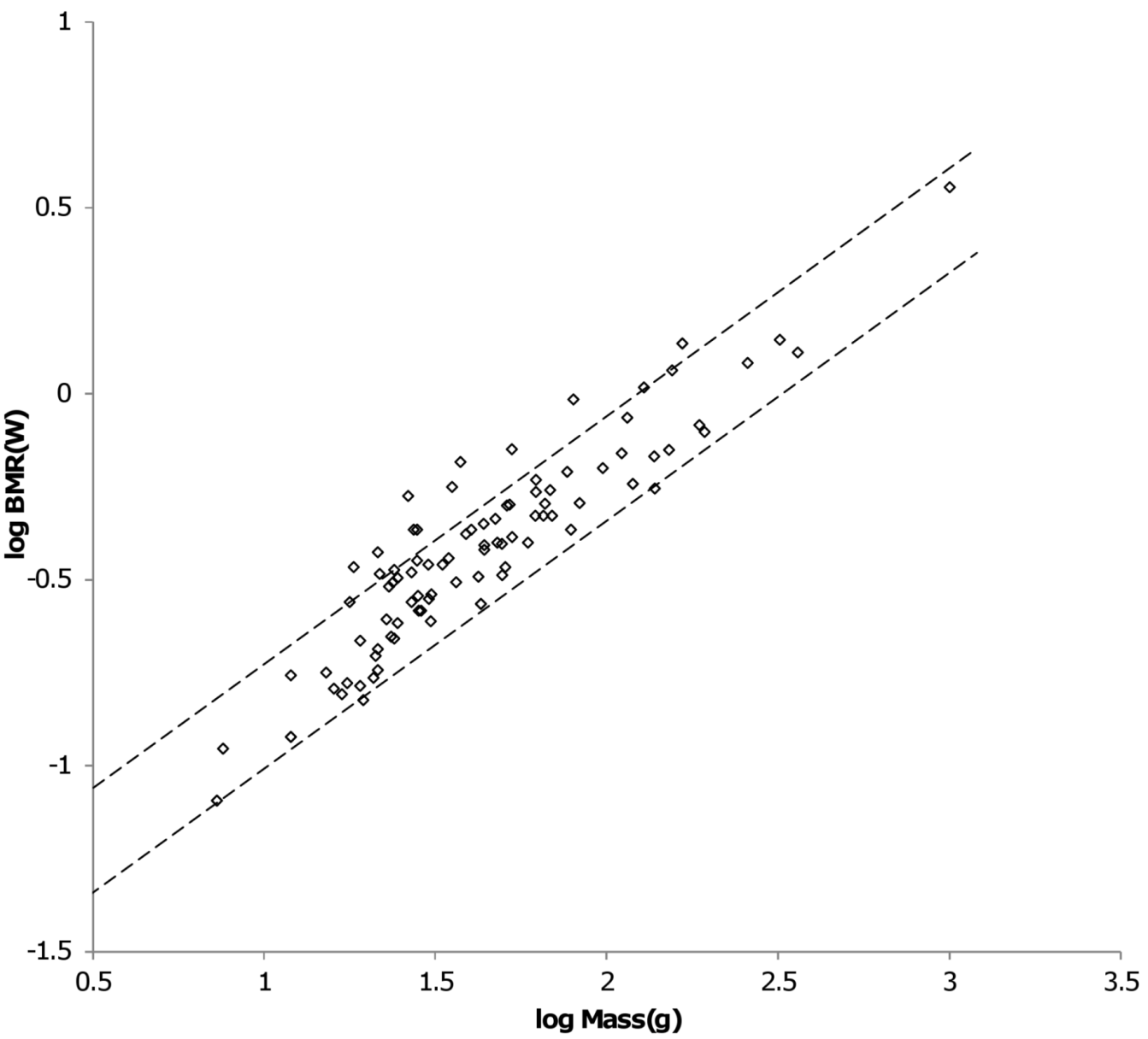


**Figure 7. Log BMR as a function of log body mass for Cricetidae Rodentia.** The Data are from (Kolokotrones et al, 2010). The dashed lines are MMLE sturdiness factor boundaries. The upper boundary was generated with a sturdiness factor s = (3)^0.5^. The lower boundary was generated with s = (3)^-0.5^. The boundary lines are for geometric similarity. The Cricetidae Rodentia species-averaged BMR, mass data are marked by open diamonds. R_M_^2^ = 0.9913.

______________________________________________________________________________

**Bats (the order Chiroptera).** Bats are second only to rodents in the number of species among mammals. Bats comprise about 12% of families, 16% of genera, and 20% of species of recent Mammals. Their masses range over three orders of magnitude from *Craseonycteris thonglongyai* and *Tylonycteris pachypus* with masses as small as 2g to *Pteropus giganteus* with a mass of as much as 1600g. (Nowak, 1999).

(Nowak, 1999) provides data in grams (g) for approximate minimum and maximum body masses. It also provides in millimeters (mm) approximate head and body lengths, forearm lengths and tail lengths when a tail is present. The data are mostly for genera with very little data for species. There is little data on wing span. From the reference (Nowak, 1999) data 350 individual and 176 taxon-averaged body mass, head and body length and forearm length samples were obtained (Data S3). (Kolokotrones et al, 2010) provides 85 species-averaged BMR and body mass samples. BMR is given in watts and mass is given in grams (g). Lengths were converted to meters (m) for calculations involving equations (1) through (5).

Bats primary means of locomotion is flying by flapping very flexible membranous wings controlled by multi-jointed fingers (Muijres et al, 2011). Unlike birds, bats use their hind limbs as well as their fore limbs to flap their wings (Norberg, 1981). Bats experience daily and seasonal fluctuations in body mass which they accommodate by changes in wing kinematics that vary among individuals (Iriarte-Diaz et al, 2012). To analyze the applicability of MMLE theory to bats, a characteristic length and a fundamental propulsion frequency related to very complicated flapping wing flight needed to be identified. The characteristic length should be related to wing dimensions. (Norberg, 1981) found that forearm length scaled with body mass with about the same exponent as wing span. Given the options available with the (Nowak, 1999) data, it was assumed that forearm length is linearly related to characteristic length. A possible complication that is avoided by this assumption is that full wing dimensions, such as wing span, in a flying bat may vary with flight mode and speed and may be different than those measured from specimens stretched out flat on a horizontal surface (Riskin et al, 2010).

(Norberg & Rayner, 1987) found that geometric similarity applied for most bat wing dimensions with some exceptions. Considering wing span, geometric similarity did not apply for all bats considered together at the 5% level of significance. It did apply to all bat families except the Vesperilionidae. It did apply to primary insectivores, carnivores and nectarivores but not to primary frugivores. More recent work suggests that wing bone lengths are also geometrically similar with respect to body mass in different sized bats (Norberg & Norberg, 2012).Thus geometric similarity, with some exceptions, seems to be the most promising path to identifying a characteristic length and fundamental propulsion frequency related to wing dimensions for bats. For MMLE theory, geometric similarity implies that the slope for the log of body mass regressed on the log of characteristic length is 3.0 and the slope of the log of BMR regressed on the log of body mass is 0.6667.

Table 3 shows the PI and AVG regression analysis results obtained with the (Nowak, 1999) forearm length data and the (Kolokotrones et al, 2010) BMR data. Geometric similarity does not apply to all bats considered together.

The order Chiroptera is divided into two suborders: the Megachiroptera consisting of the single family Peteropodidae and the Microchiroptera consisting of all other bats (Nowak, 1999). From table 3, geometric similarity applies to neither suborder. The mass scaling exponent x for equation (4) is approximately 2.5 for both AVG and PI regressions for Microchiroptera which would indicate y = 0.8. As will be discussed shortly, for bats y = 2/3 is better supported by the data.

__________________________________________________________

**Table 3.** Results of Bat regression Analyses

| **Order or** | **Regression** | **Independent** | **Dependent** |  |  |  | **Number** |
| --- | --- | --- | --- | --- | --- | --- | --- |
| **Family** | **Type** | **Variable** | **Variable** | **Slope** | **Intercept** | **R^2^** | **Samples** |
| All Bats | PI(0.93) | Length(mm) | Mass(g) | 2.6668 | -3.3211 | 0.8507 | 176 |
|  | AVG | Length(mm) | Mass(g) | 2.7718 | -3.3628 | 0.9924 | NA |
| Megachiroptera | PI(0.33) | Length(mm) | Mass(g) | 2.8628 | -3.4933 | 0.9749 | 40 |
|  | AVG | Length(mm) | Mass(g) | 2.7335 | -3.2632 | 0.9978 | NA |
| Microchiroptera | PI(0.93) | Length(mm) | Mass(g) | 2.515 | -3.1025 | 0.7522 | 136 |
|  | AVG | Length(mm) | Mass(g) | 2.5292 | -2.9844 | 0.9928 | NA |
| Heavy Bats | PI(0.79) | Length(mm) | Mass(g) | 2.7595 | -3.3252 | 0.922 | 86 |
|  | AVG | Length(mm) | Mass(g) | 2.7233 | -3.2329 | 0.9937 | NA |
| Light Bats | PI(0.0) | Length(mm) | Mass(g) | 3.28 | -4.4926 | 0.8781 | 29 |
|  | AVG | Length(mm) | Mass(g) | 2.9878 | -3.9552 | 0.9743 | NA |
| All Bats | PI(0.83) | Mass(g) | BMR(watts) | 0.8111 | -1.9063 | 0.8784 | 84 |
| Megachiroptera | PI(0.56) | Mass(g) | BMR(watts) | 0.8581 | -2.0161 | 0.9279 | 21 |
| Microchiroptera | PI(1.07) | Mass(g) | BMR(watts) | 0.7459 | -1.8247 | 0.991 | 63 |
| Heavy Bats | PI(0.89) | Mass(g) | BMR(watts) | 0.8225 | -1.9166 | 0.8887 | 51 |
| Light Bats* | PI(0.0) | Mass(g) | BMR(watts) | 0.7015 | -1.8048 | 0.8154 | 17 |

The regression expressions are: Log(dependent variable) = slope X log(independent variable) + intercept. PI(n) means the phylogenetic informed regression method using BayesTraits and the number in parentheses is the estimated value of lambda. AVG means the cohort averaging regression method. Length(mm) is forearm length in millimeters. Mass(g) is body mass in grams. NA means Not Applicable. *Data available for only 4 of the 8 families comprising light bats.

_________________________________________________________

The situation in which log body mass for all members of an order scaled with a slope that is less than that expected for geometric similarity was encountered with rodents. In that case it indicated that the order was composed of two subpopulations: one in which geometric similarity applied and another in which a different type of similarity applied. Figure 8 offers such an explanation for bats. In Fig. 8 the bat families have been divided into three groups: ‘heavy’ bats, ‘light’ bats and ‘intermediate’ bats. At the same forearm length members of families composing the heavy bats are mostly more massive than those of the families composing the light bats. Intermediate bats span both the heavy and light mass regimes. The families composing the three groups are given in the caption of Fig. 8.

Table 3 shows that the log body mass slope for light bats is greater than the log body mass slope for heavy bats. For the AVG regression of log mass on log forearm length the slope for light bats is very nearly the 3.0 expected for geometric similarity. The PI regression slope is even greater.

Pteropodidae and Phyllostomidae comprise the heavy bats. The Pteropodidae contain the Old World frugivores and the Phyllostomidae contain the New World frugivores. While both families have species with other diets, the frugivores have wings adapted to commuting long distances from roost to feeding areas (Norberg & Rayner, 1987).The relationships between wing dimensions and body mass should be similar for the frugivore members of the two families.

The fundamental propulsion frequency for bats should be related to the wing flapping frequency. A bat’s wing flapping frequency increases slightly with air speed at lower speeds. It becomes almost independent of speed at higher speeds. The speed at which the transition occurs is the preferred speed (Bullen & McKenzie, 2002). The wing flapping frequency at this preferred speed should be proportional to the fundamental propulsion frequency.

Animals that fly by flapping wings operate in a narrow range of Strouhal numbers in cruising flight (Taylor, Nudds & Thomas, 2003). Strouhal number does not change significantly for Pteropodidae (Riskin et al, 2010). It appears to be an accurate predictor of wingbeat frequency for birds (Nudds, Taylor & Thomas, 2004). Thus Strouhal dynamic similarity is a prime candidate for the type of dynamic similarity that determines the fundamental frequency of propulsion for bats.

As noted in the summary of MMLE theory the fundamental frequency of propulsion would be proportional to the inverse square of the characteristic length if both Strouhal and Reynolds dynamic similarity applied simultaneously. By equation (3) this would result in body mass being proportional to the characteristic length raised to a power greater than 3. This situation is not observed in the Table 3 regression results except for PI mass regressed on forearm length for light bats. However since the AVG result is so close to geometric similarity this was interpreted as support for geometric similarity with y = 2/3 as was done with Cricetidae Rodentia. It is noted that the characteristic length for viscous drag and that for vortex growth and shedding could be different body dimensions.

For bats Strouhal number = ( wingbeat frequency)X(wingbeat amplitude)/(air speed). Wingbeat amplitude is the vertical distance the wing tip travels during a stroke. Under Strouhal dynamic similarity the wingbeat amplitude should be proportional to the MMLE characteristic length. It

______________________________________________________________________________


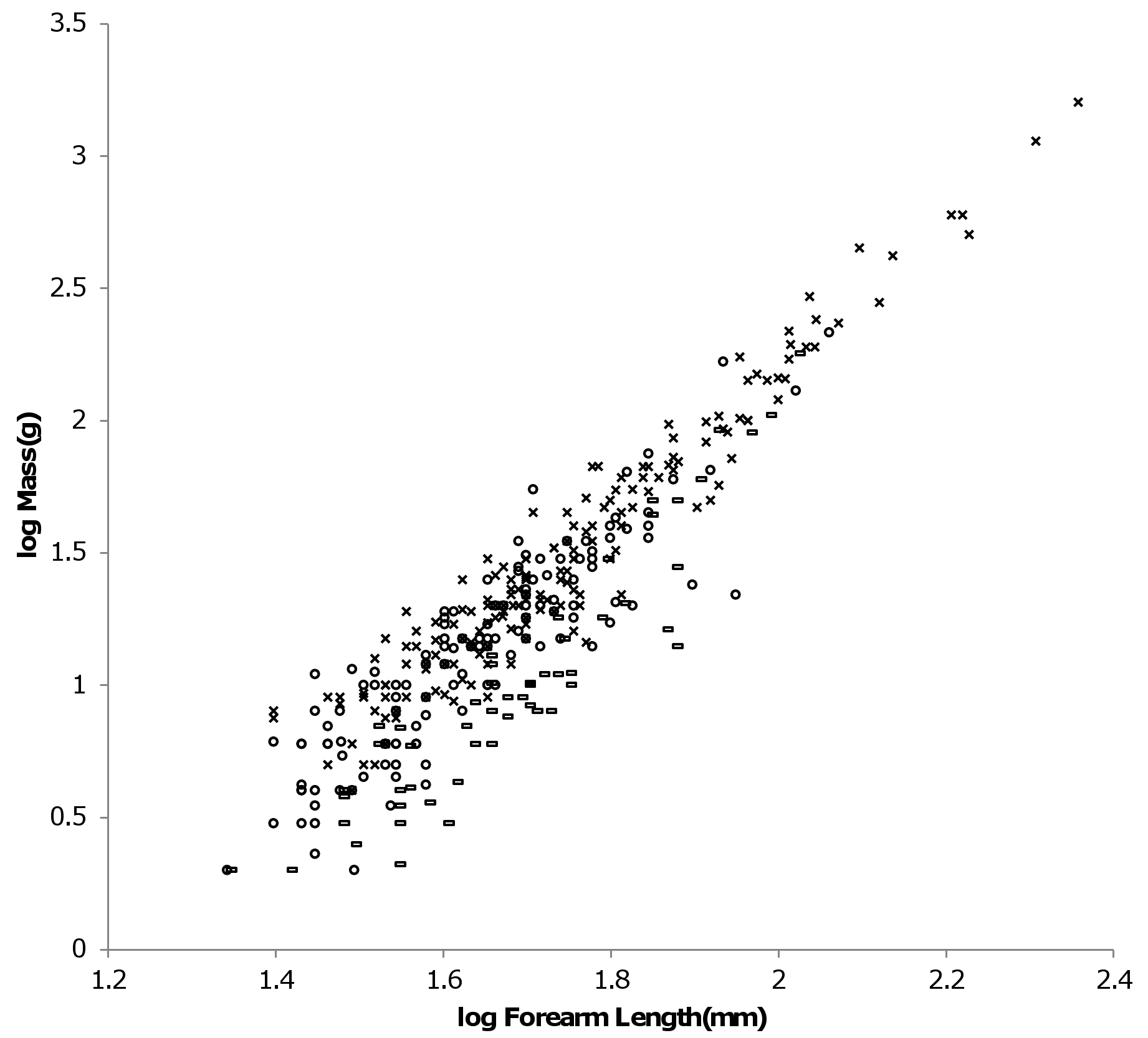


**Figure 8. Log body mass as a function of log forearm length for bats.** Data are from (Nowak, 1999). At the same forearm length individuals from families composing the ‘heavy’ bats marked with Xes are mostly more massive than those from the families composing the ‘light’ bats marked with open rectangles. ‘Intermediate’ bats marked with open circles span both the heavy and light mass regimes. The families Pteropodidae and Phyllostomidae comprise the heavy bats. The families Emballonuridae, Craseonycteridae, Rhinopomatidae, Rhinolophoidea, Mormoopidae, Noctilionidae, Furipteridae and Hipposideridae comprise the light bats. The families Nycteridae, Megadermatidae, Vespertilionoidae, Thyropteridae, Myzopodidae, Natalidae, Mystacinidae, and Molossidae comprise the intermediate bats.

______________________________________________________________________________

was assumed that wingbeat amplitude characteristic length is a fraction of forearm length for bats.

Strouhal dynamic similarity is consistent with geometric similarity. Since the fundamental propulsion frequency under geometric similarity is proportional to the inverse of the characteristic length, simultaneous geometric and Strouhal similarity imply that the preferred flight speed is approximately constant.

Assuming geometric similarity and sturdiness boundaries of (3)^0.5^ and(3)^-0.5^  in equations (2) and (3), the scaling fraction linearly relating forearm length to characteristic length for light bats was varied until R_M_^2^ for BMR was maximized. The corresponding value for the characteristic length scaling fraction is 0.61 meaning that characteristic length is approximately 0.61 of forearm length. The maximized R_M_^2^ for BMR is 0.9984. R_M_^2^ for BMR was used because R_M_^2^ for body mass was a constant 1.0 over the range of scaling fractions examined from 0.6 to 1.0.

The forearm length is 47.9mm for the mid point of the light bat log forearm lengths. The associated body mass is 11.6g by the AVG regression relationship of table 3. Using these values of forearm length and body mass for geometrically similar light bats, equations (3) was solved for the combined fundamental propulsion frequency and dynamic similarity constants, kc, for each value of the characteristic length scaling fraction. For the value of 0.61 for the scaling fraction, kc = 0.625 m/sec. The running/walking placental mammal derived values for the constants G_m_, e and G_o_ were used in equation (3) and the carnivore derived value for G_r_ was used in equation (2).

Since R^2^ is nearly unity for the AVG regression relationship of Table 3, equation (6) should apply. The log likelihood for the resulting exponent, 2/x = 0.6694, could not calculated using BayesTraits. Thus the significance of its difference from the BMR regressed on mass exponent in Table 3 could not be determined. It is noted that length and mass data was available from (Nowak, 1999) to estimate x from for all families composing the light bats group but BMR and mass data from (Kolokotrones et al, 2010) to estimate the BMR regressed on mass exponent was available for only four of the eight families.

Figure 9 shows the MMLE BMR as a function of body mass boundaries for geometric similarity for light bats evaluated with these estimates together with the (Kolokotrones et al, 2010) BMR and body mass samples for light bats.

A methodology similar to that used for light bats was used to establish the characteristic length scaling fraction and fundamental propulsion frequency constant for heavy bats. By the heavy bat regression relationships of table 3, heavy bats are not geometrically similar. Since heavy bats are not geometrically similar the exponent, r, in the relationship between fundamental propulsion frequency and characteristic length in equations (3) and (5) also had to be established.

The forearm length is 75.9mm for the mid point of the heavy bat log forearm lengths. The corresponding body mass is 77.1g by the AVG regression relationship and 73g for the PI relationship. The value of x in equation (4) is 2.72 for the AVG regression relationship and 2.76 for the PI relationship of table 3. Both values of x are consistent with y = 2/3. These values were used in equations (3) and (5) to solve for the characteristic length scaling fraction, the exponent r and the combined fundamental propulsion frequency and dynamic similarity constants, kc. This was done by varying r for each trial value of the characteristic length scaling fraction until a pair of values for the characteristic scaling fraction and r was found that maximized R_M_^2^ for BMR. For the AVG relationship the value for the characteristic scaling fraction is 0.92, the value for the exponent r is 0.68 and the value for the combined fundamental propulsion frequency and dynamic similarity constants is kc = 3.22 m^0.68^/sec. The corresponding value of R_M_^2^ for BMR is 0.9828. For the PI relationship the value for the characteristic scaling fraction is 0.86, the value for the exponent r is 0.73 and the value for the combined fundamental propulsion frequency and dynamic similarity constants is kc = 2.59 m^0.73^/sec. The corresponding value of R_M_^2^ for BMR is 0.9918. As with light bats, R_M_^2^ for BMR was used because R_M_^2^ for body mass was very nearly 1.0 over the range of parameters examined. In terms of R_M_^2^ the PI and AVG results are nearly indistinguishable.

Since R^2^ is nearly unity for the heavy bat AVG mass regressed on length relationship in Table 3, equation (6) should apply. The difference between the resulting exponent, 2/x = 0.7344 and the PI BMR regressed on mass exponent in Table 3 is borderline significant as the log likelihood ratio is 4.1 (Pagel, 1999).

Figure 9 also shows the MMLE log BMR as a function of log body mass MMLE sturdiness factor boundaries for heavy bats evaluated with these estimates together with the (Kolokotrones et al, 2010) species-averaged BMR and body mass samples for heavy bats.

Figure 10 shows the MMLE log body mass as a function of log forearm length MMLE sturdiness factor boundaries for both heavy bats and geometrically similar light bats. The (Nowak, 1999) heavy and light bat mass and forearm length data are also shown.

Because the exponent r in equation (3) is less than unity for heavy bats, the fundamental propulsion frequency should scale with bat dimensions differently than what would be expected for geometric similarity. Also the Strouhal number or preferred flight speed, or both, should scale with body dimensions. For Megachiroptera, the inverse of wingbeat frequency scales with body mass significantly more shallowly than expected with geometric similarity. Both Strouhal number and preferred flight speed scale very slightly with body mass but this is not considered to be significant (Riskin et al, 2010).

From table 3 the AVG regression relationship between body mass and forearm length are nearly identical for heavy bats and the Megachiroptera suborder of bats. Body mass approximately scales as forearm length raised to the 2.72 power. The coefficient of determination, R^2^, is almost

______________________________________________________________________________


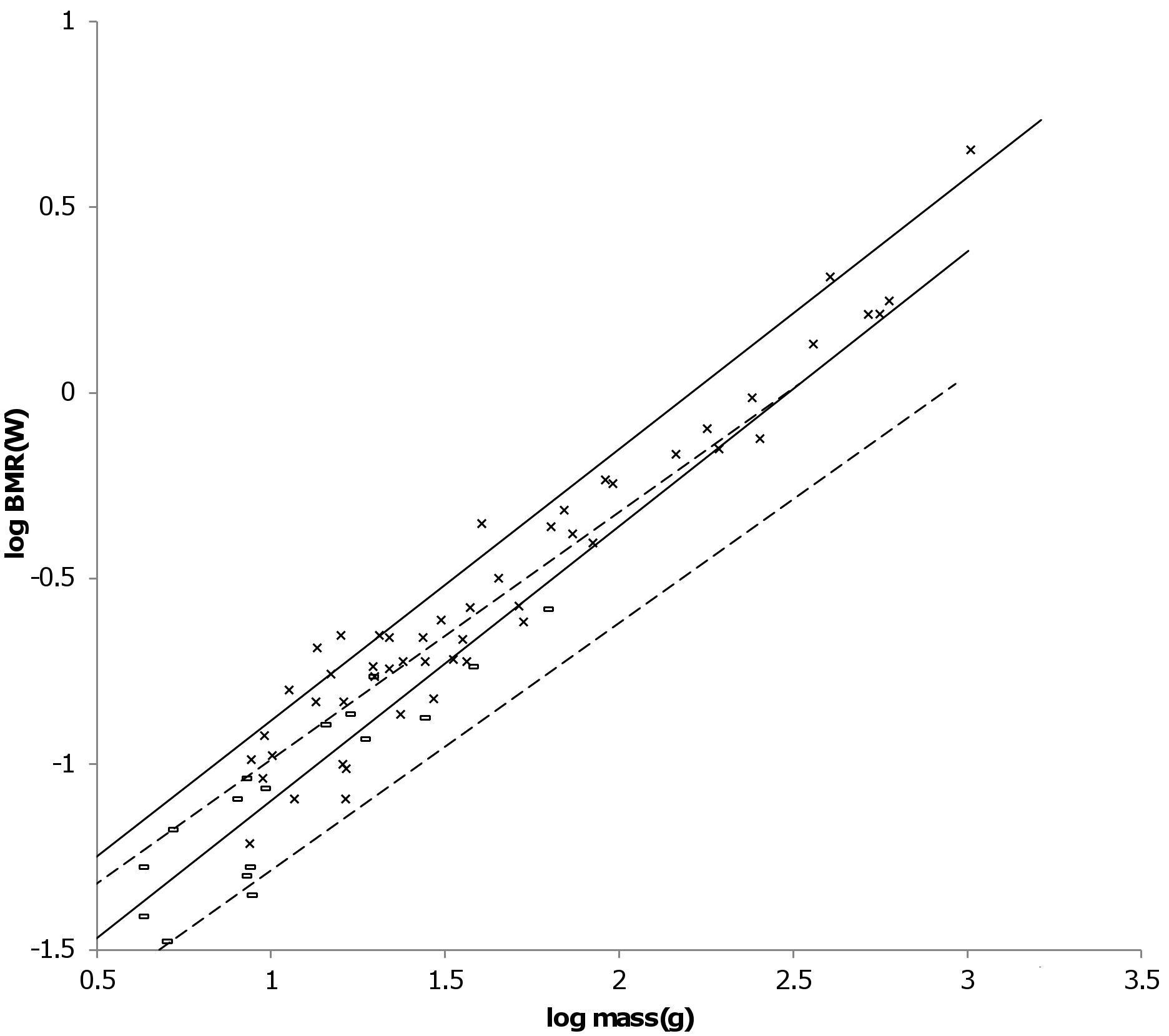


**Figure 9. Log BMR as a function of log body mass for heavy and light bats.** Data are from (Kolokotrones et al, 2010). The solid and dashed lines are the MMLE sturdiness factor boundaries. The upper boundaries were generated with a sturdiness factor s = (3)^0.5^. The lower boundaries were generated with s = (3)^-0.5^. $\sqrt{3}.$The steeper sloping solid boundary lines are for heavy bat Strouhal dynamic similarity. The shallower sloping dashed boundary lines are for light bat geometric similarity. R_M_^2^ = 0.9828 for heavy bats with respect to the Strouhal boundaries. R_M_^2^ = 0.9984 for light bats with respect to the geometric boundaries. Heavy bat species-averaged data are marked with Xes. Light bats species-averaged data are marked with open rectangles. Data was available for only four of the eight families comprising the light bats.

______________________________________________________________________________

______________________________________________________________________________


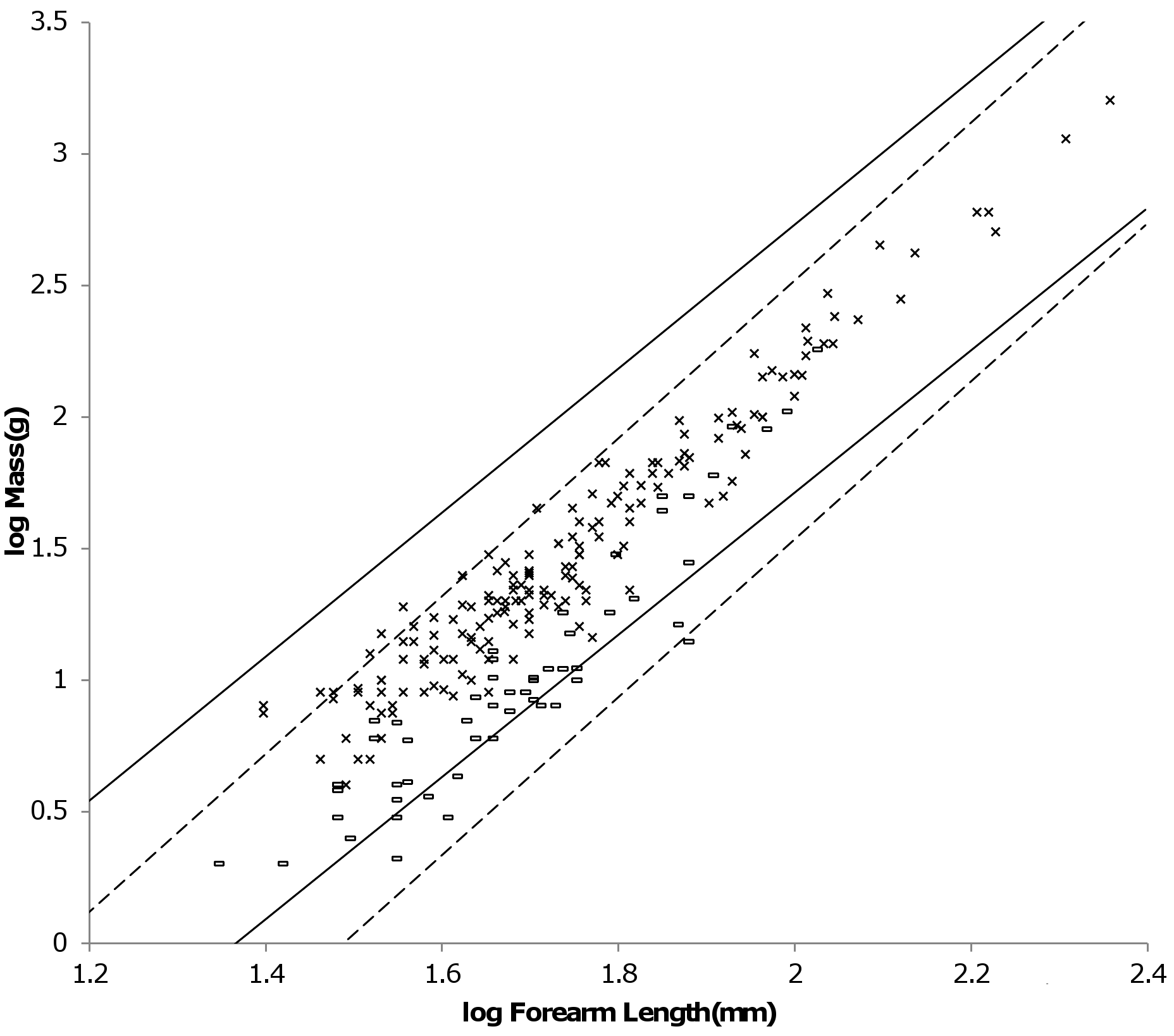


**Figure 10. Log body mass as a function of log forearm length for heavy and light bats.** Data are from (Nowak, 1999). The solid and dashed lines are the MMLE sturdiness factor boundaries. The upper boundaries were generated with a sturdiness factor s = (3)^0.5^. The lower boundaries were generated with s = (3)^-0.5^. The shallower sloping solid boundary lines are for heavy bat Strouhal dynamic similarity. The steeper sloping dashed boundary lines are for light bat geometric similarity. R_M_^2^ = 0.9982 for heavy bats with respect to the Strouhal boundaries. R_M_^2^ = 1.0 for light bats with respect to the geometric boundaries. Individual heavy bats are marked with Xes. Individual light bats are marked with open rectangles.

______________________________________________________________________________

______________________________________________________________________________


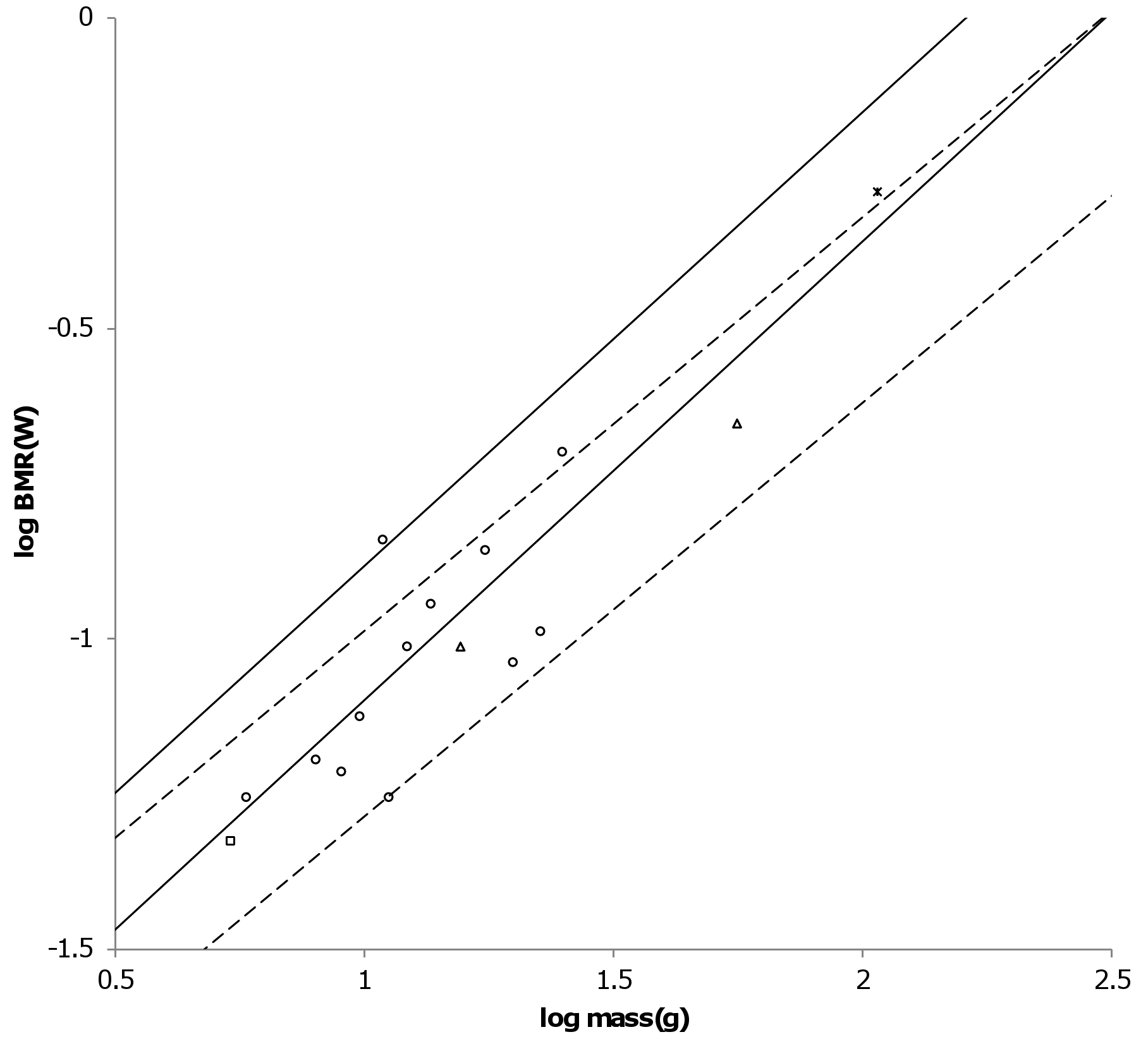


**Figure 11. Log BMR as a function of log body mass for intermediate bats.** Data are from (Kolokotrones et al, 2010). The solid and dashed lines are the MMLE sturdiness factor boundaries. The upper boundaries were generated with a sturdiness factor s = (3)^0.5^. The lower boundaries were generated with s = (3)^-0.5^. $\sqrt{3}.$The steeper sloping solid boundary lines are for heavy bat Strouhal dynamic similarity. The shallower sloping dashed boundary lines are for light bat geometric similarity. Data was available for only four of the eight families comprising the intermediate bats. R_M_^2^ = 0.9678 for intermediate bats with respect to the Strouhal boundaries. R_M_^2^ = 0.9893 for intermediate bats with respect to the geometric boundaries. R_M_^2^ = 0.9998 for intermediate bats with respect to both sets of boundaries. The data are species-averaged. Megadermatidae are marked with crossed Xes. Molossidae are marked with open triangles. Vesperstilionidae are marked with open circles. Natalidae are marked with open squares.

______________________________________________________________________________

______________________________________________________________________________


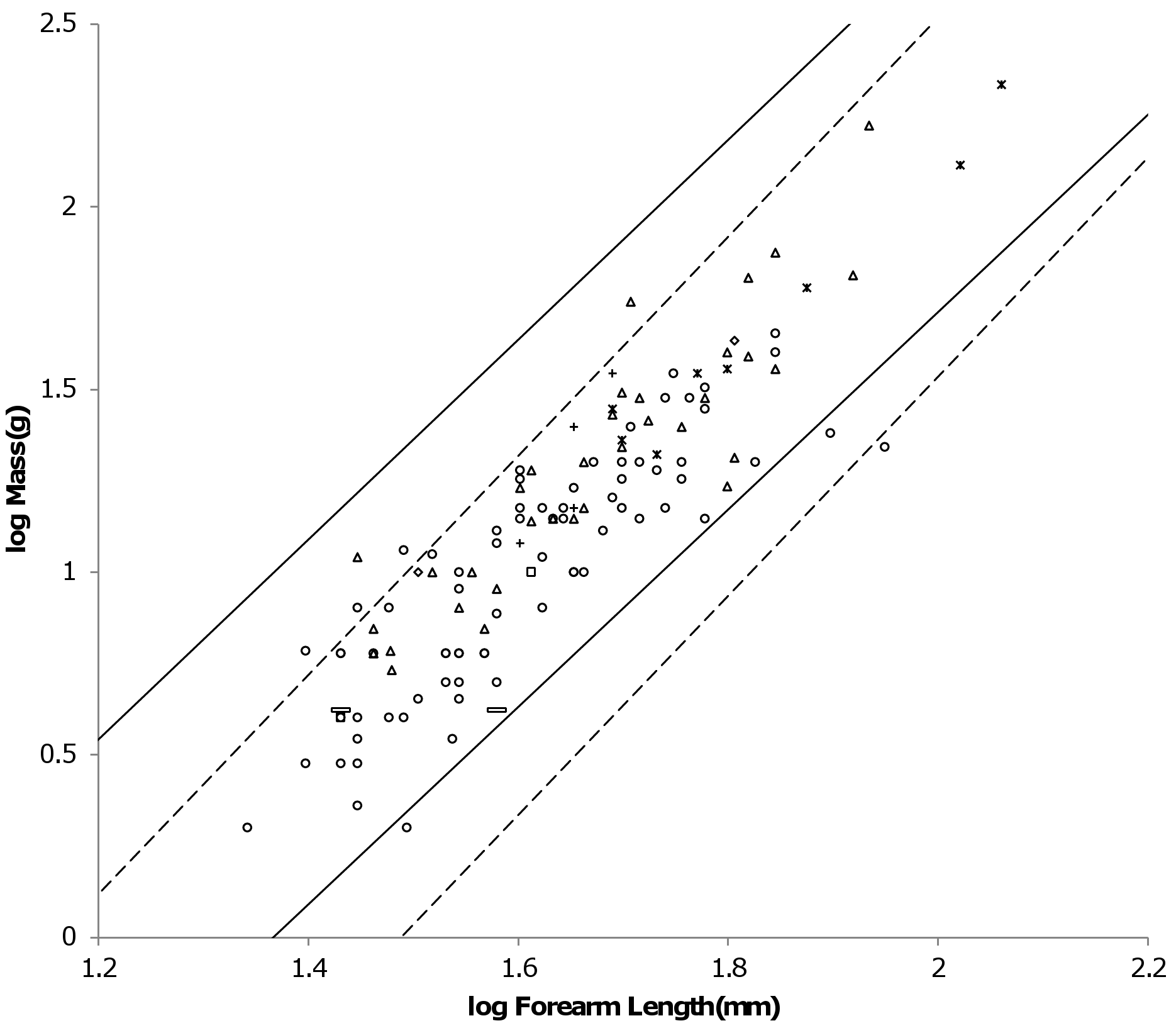


**Figure 12. Log body mass as a function of log forearm length for intermediate bats.** Data are from (Nowak, 1999). The solid and dashed lines are the MMLE sturdiness factor boundaries. The upper boundaries were generated with a sturdiness factor s = (3)^0.5^. The lower boundaries were generated with s = (3)^-0.5^. The shallower sloping solid boundary lines are for heavy bat Strouhal dynamic similarity. The steeper sloping dashed boundary lines are for geometric similarity. R_M_^2^ = 0.998 for intermediate bats with respect to the Strouhal boundaries. R_M_^2^ = 0.9981 for intermediate bats with respect to the geometric boundaries. R_M_^2^ = 0.9999 for intermediate bats with respect to both sets of boundaries.

______________________________________________________________________________

1.0 which allows the mass-length relationship to be accurately inverted so that forearm length scales as body mass raised to the 0.37 power. Since the heavy bat fundamental propulsion frequency, f, scales as forearm length raised to the -0.68 power, f scales as body mass raised to the -0.25 power. (Norberg & Norberg, 2012) found f scaled as mass raised to the -0.276 power$\propto$, R^2^ = 0.932, for Megachiroptera.

Figure 11 shows the MMLE log BMR as a function of log body mass MMLE sturdiness factor boundaries for both heavy bats and light bats. The (Kolokotrones et al, 2010) species averaged BMR and body mass samples for intermediate bats are also shown. The data spreads over both MMLE bands as if within the same family there are species that conform to geometric similarity and other species that conform to heavy bat similarity.

Figure 12 shows the MMLE log body mass as a function of log forearm length MMLE sturdiness factor boundaries for both heavy and light bats. The (Nowak, 1999) body mass and forearm length samples for intermediate bats are also shown. Intermediate bat body mass is explained almost equally well by either the geometrically similar light bat MMLE band or the heavy bat MMLE band. R_M_^2^ = 0.9981 for the light bat MMLE band. R_M_^2^ = 0.9980 for the heavy bat MMLE band. R_M_^2^ = 0.9999 for the two bands together.

The light and heavy bat data in Fig. 10 does not fully occupy their respective MMLE sturdiness factor bands. This raises the possibility that a sturdiness factor range narrower than (3)^-0.5^ to (3)^0.5^ may apply to heavy or light bats. The intermediate bat data in Fig. 12 more completely occupies both bands indicating that the full sturdiness factor range is applicable. The situation may be more complicated. Each of the heavy, light and intermediate bat groups contains species with different food habits. Bat wing morphology is associated with flight behavior related to food habits. Even among the insectivores that dominate the light and intermediate groups there are different styles of catching insects that are associated with differing wing morphologies (Norberg & Rayner, 1987; Norberg & Norberg, 2012). Dynamic similarity may apply at a phylogenetic level below the family.

The light-intermediate-heavy representation of bats is better supported by the data than either the all bats representation or the Megachiroptera-Microchiroptera representation in terms of R_M_^2^ and coverage, R. For mass and forearm length both R_M_^2^ and R are very nearly 1.0 for all three representations. For BMR and body mass R_M_^2^ = 0.9882 and R = 0.73 for the all bats representation when computed using the AVG mass and forearm length regression relationship in table 3 but they reduce to R_M_^2^ = 0.9418 and R = 0.39 when using the PI relationship. For the light-intermediate-heavy representation the combined R_M_^2^ = 0.99 and the combined R = 0.7 using both the PI and the AVG relationships. For the Megachiroptera-Microchiroptera representation the combined R_M_^2^ = 0.98 and the combined R = 0.4 using either relationship. The poorer performance of the Megachiroptera-Microchiroptera representation is due to low coverage of Microchiroptera data computed with y = 0.8 which is consistent with both the PI and AVG relationships in table 3: R is only 0.24 for the Microchiroptera data.

MMLE can exactly predict every body mass, head and body length datum from (Nowak, 1999) for bats that is shown in Fig. 8, 10 and 12. There is no error and thus no unexplained variance. A separate characteristic length scaling fraction is needed for light bats and for heavy bats to translate the available forearm length data to characteristic lengths. Then using the parameter values that were established for running/walking placental mammals, computing an individual light bat’s body mass from its forearm length with equation (3) requires specifying its sturdiness factor, geometric muscle similarity and the light bat combined fundamental propulsion frequency and dynamic similarity constants, kc. Computing a heavy bat’s body mass from its forearm length with equation (3) requires specifying its sturdiness factor, the heavy bat fundamental propulsion frequency exponent, r, and the heavy bat combined fundamental propulsion frequency and dynamic similarity constants, kc. Computing an intermediate bat’s body mass from its forearm length may require evaluating the animal as a light bat and as a heavy bat and then deciding which classification better agrees with the datum.

MMLE can also predict every BMR and body mass datum from (Kolokotrones et al, 2010) for bats that is shown in Fig. 9 and 11 by using equations (2) and (3) simultaneously. There is no error and thus no unexplained variance. Body mass is computed with equation (3) as just described. Then the Carnivore value of the constant G_r_ established for running/walking placental mammals is used in equation (2) to compute the bat’s BMR from its forearm length and sturdiness factor. Some iteration of this process may be required if the head and body length and/or sturdiness factor is not included with the BMR and body mass data for the animal.
