# Supplementary material for: An explanation of the relationship between mass, metabolic rate and characteristic length for placental mammals
Source: PeerJ. 2015 Sep 3;3:e1228. doi: 10.7717/peerj.1228 (PMC4562252; doi:10.7717/peerj.1228)
Supplement: Article S2 [file peerj-03-1228-s005.docx]

**Summary of MMLE Theory.** This section presents an abbreviated version of MMLE theory as originally formulated (Frasier, 1984) together with some necessary modifications.

Nearly all of the energy flux that is measured as basal metabolic rate (BMR) is generated by mitochondria. Although there are other ways to measure it, BMR is most commonly measured by oxygen consumption (Hulbert & Else, 2004). The oxygen is consumed by processes that pump protons across the mitochondrion inner membrane. Heat is produced when some of the protons leak back across the membrane in a controlled fashion as in brown fat or as an uncontrolled basal leak. Otherwise the protons cause the phosphorylation of adenosine diphosphate (ADP) to adenosine triphosphate (ATP) as they return across the inner membrane (Jastroch et al, 2010). ATP is the fuel that powers animal tissues.

MMLE strives to predict the absolute value of the BMR of an animal rather than the exponent b or the constant a in the relationship aW^b^. It calculates BMR by summing the energy allocation to an animal’s tissues. The energy allocated to a tissue type is proportional to the number of mitochondria in the tissue. Thus MMLE tries to count the mitochondria in the tissues that compose an animal and then sum these counts for the entire animal. This approach to calculating an animal’s BMR was proposed by (Smith, 1956).

MMLE also proposes that mitochondria have a lower limit for the rate of energy release that is experienced by an adult in the thermoneutral zone when inactive and post-absorptive. Basal metabolic rate (BMR) is experienced when an animal’s mitochondria are in this state. The lower limit for the rate of energy release by a mitochondrion can be different for different tissue types and different phylogenetic groups.

The skeletal muscles, heart, kidneys, liver and brain dominate BMR for vertebrates. These tissues account for 75% to 81% of BMR in humans (Müller et al, 2013). The heart, kidneys, liver and brain account for more than 60% of human BMR while accounting for less than 6% of body mass. The sum of the energy consumption by each of the non-skeletal muscle tissues that dominate BMR appears to scale with body mass with the same exponent b in the relationship BMR = aW^b^ as does BMR for the entire body even though the energy consumption for each separate tissue type may not (Wang et al, 2001).

Mitochondria are distributed approximately uniformly throughout a muscle. In the other tissues that dominate BMR the mitochondria are distributed in a relatively thin surface that surrounds material containing few mitochondria (Spence & Mason, 1979). The original paper examines the anatomy of the vertebrate liver in detail to show that it is composed of thin surfaces of hepatic cells richly endowed with mitochondria surrounding endothelial linings, blood, bile and other materials with relatively few mitochondria. For these reasons MMLE theory uses a simplified model of a vertebrate’s body consisting of two components: 1) volume active tissues in which mitochondria are approximately uniformly distributed throughout the tissue; and 2) surface active tissues in which mitochondria are concentrated in a surface that surrounds materials that contain few mitochondria.

Because the skeletal musculature dominates the volume active tissues from an energy perspective, these tissues were called “skeletal musculature” in the original paper. The surface active tissues that are dominated by the heart, kidneys, liver and brain were called “non-skeletal musculature”.

It was argued in the original paper that the density of mitochondria in the cells composing the surface active tissues was the maximum possible consistent with the functioning of the cells. The skeletal musculature is sized to support the mechanical loads imposed by activity. The density of mitochondria in the skeletal musculature is sufficient to support the mechanical power requirements of the activity.

In the BMR state skeletal muscle mitochondria are only supporting local maintenance functions. Mitochondria in the rest of the body may be operating at a greater output level to support their local maintenance functions, the other components of the non-skeletal muscle tissues and the skeletal muscles. Thus the minimum rate of energy release by a mitochondrion should occur with skeletal muscle mitochondria in the BMR state. MMLE uses this possibility to express the rate of energy release by mitochondria in other tissues in the BMR state and in all tissues in non-BMR activity states as amplification constants multiplying the skeletal muscle mitochondrion rate of energy release in the BMR state.

MMLE theory proposed that the minimum rate of energy release by a skeletal muscle mitochondrion in the BMR state can be different for different phylogenetic groups.

Mitochondria have different respiration rates in different phylogenetic groups (Else & Hulbert, 1981; Else & Hulbert, 1985; Hulbert & Else, 2004; Guderley et al, 2005). Assuming that the BMR state minimum rate of skeletal mitochondrion energy release is genetically determined, a difference between related phylogenetic groups implies mutations affecting mitochondrion performance. Mutations in mitochondria DNA that could affect ATP production were found in all of 41 species of mammals that were examined (da Fonseca et al, 2010). But the situation could be more complex as mitochondrial function plastically responds to change through DNA transcription control, mitochondrial membrane composition and proton leak (Seebacher et al, 2010).

Because a value for the minimum rate of energy release by a skeletal muscle mitochondrion in the BMR state was not available, it was replaced by a non-dimensional parameter, e, which was named the ‘mitochondrion capability quotient’. The parameter e is defined as the minimum rate of energy release by a skeletal muscle mitochondrion in the BMR state for a particular phylogenetic group relative to the minimum rate of energy release by a skeletal muscle mitochondrion in the BMR state for walking/running placental mammals. This definition will be further restricted when walking/running placental mammals are addressed later in the present paper.

Although it was not addressed in the original paper, the mitochondrion capability quotient should be, among other things, a function of body temperature.

Skeletal muscle mitochondria require the support of the functions powered by the mitochondria in the non-skeletal musculature. MMLE theory considers this to be a constraint in that at levels of muscle activity that can be sustained long term without the accumulation of an oxygen debt, the total power produced by muscle mitochondria is proportional to the total power produced by mitochondria in the non-skeletal musculature. Since the total power produced in a tissue type is (e) X (the number of mitochondria in the tissue) X (the amplification constant appropriate for the tissue and the host animal’s activity state), it was concluded the power produced by skeletal muscle mitochondria in the BMR state is also proportional to the power produced by mitochondria in the non-skeletal musculature in the BMR state. Since BMR is the sum of these two power components, BMR is equal to the power produced by mitochondria in the non-skeletal muscle body component multiplied by an appropriate constant.

This summary of MMLE theory will use the term ‘appropriate constant’ for intermediate constants needed to make an intermediate relationship an equality. When the intermediate relationships have been combined into the final MMLE equations the constants occurring in those equations will be given unique symbols. The mathematically rigorous derivations are available in the original paper.

For skeletal muscle there are additional constraints. The power generated by mitochondria is also proportional to the mechanical power that a muscle must generate during normal activity. The mechanical power is (the force the muscle bears) X (the distance the muscle extends/contracts) X (the frequency of extension/contraction) where each term is evaluated for the host animal’s activity state. The power is also proportional to (e) X (the mass specific density of mitochondria in the muscle) X (the muscle’s mass) X (the amplification constant appropriate to the activity state of the animal). By analyzing these constraints with respect to the sliding filament theory of muscle contraction, the MMLE theoretical analysis concluded that they could be simultaneously satisfied if the mass of the skeletal muscles were proportional to BMR divided by the fundamental frequency of muscle activity.

The actual frequency at which a muscle operates is a multiple of the fundamental frequency. The multiplier is a function of an animal’s activity state.

Propulsion was considered to be the primary power consuming activity of skeletal muscles for vertebrates. For animals employing similar dynamics for propulsion, muscle mass can be equated to (an appropriate constant) X ( BMR) divided by (the fundamental propulsion frequency). To account for different propulsion dynamics, the fundamental frequency was multiplied by yet another appropriate constant.

This was how the original paper recognized that dynamic similarity could be used to scale muscle mass for animals of different sizes. Alexander (Alexander, 2005) addressed dynamic similarity in animals in terms of Froude, Reynolds, and Strouhal similarity. This is a physically more meaningful way to address dynamic similarity. MMLE theory in the present paper is modified to embrace these types of dynamic similarity.

In the non-skeletal muscle tissues that dominate BMR, the mitochondria are distributed in a relatively thin surface that surrounds material containing few mitochondria. Recognizing this, MMLE theory describes the mass of the non-skeletal musculature in terms of the total surface area of the energetically active surfaces.

Being a surface, the non-skeletal muscle surface can be mathematically described as the square of a length multiplied by an appropriate constant. Any length could be used as long as the constant is adjusted to make the relationship exact. For MMLE theory the selected length is one that is related to propulsion dynamics. This selected length is called the ‘characteristic length’, l. The proportionality constant includes the ‘sturdiness factor’, s. s is non-dimensional.

It is the expression of the sum of the areas of the metabolically active surfaces of the non-skeletal musculature as proportional to (sl)^2^ that is the fundamental innovation of MMLE theory.

The foregoing considerations were consolidated to express the mass of the non-skeletal musculature equal to ((sl)^2^mG_o_/e)^1/y^ where mG_o_ is the final appropriate proportionality constant. G_o_ has the same value and physical dimensions for all values of y. m is a dimensionality factor that adjusts the physical dimensions of this expression to mass. m is determined by y. m is not an independent variable. The dimensionality factor, m, was not part of the original paper.

The mass of the skeletal musculature is equal to (sl)^2^G_m_/kfe where f is the fundamental frequency of propulsion, and G_m_ and k are the final appropriate proportionality constants. Since total body mass, W, is the sum of these two components:

W = (sl)^2^G_m_/kfe + ((sl)^2^mG_o_/e)^1/y^ (1)

The basal metabolic rate, BMR, is:

BMR = G_r_(sl)^2^ (2)

where G_r_ is the final appropriate proportionality constant.

After analyzing data for running/walking mammals, rodents and bats a general formulation for the fundamental propulsion frequency appears to be f = c/l^r^ where c is the appropriate proportionality constant and r is an exponent with a value between 0.5 and 1.0 for species in these mammal orders. Substituting this expression for f in equation (1) yields:

W = s^2^ l^(2+r)^G_m_/kce + ((sl)^2^mG_o_/e)^1/y^ (3)

Animals that are dynamically similar have similar values for the exponent, r.

G_o_ is defined so that m is dimensionless with a value of 1.0 for geometrically similar non-skeletal musculature for which y = 2/3.

The equation for skeletal muscle mass includes two final appropriate constants: G_m_ and k. k is a function of the type of dynamic similarity that applies to the type of propulsion used by an animal. G_m_ and k were defined so that k is non-dimensional with a value of 1.0 for running/walking placental mammals.

The dimensions of the final appropriate constants in equations (1) and (2) are physically straight forward. In the meters kilograms seconds (MKS) system of physical units G_m_ has the dimensions (kg/m^2^ sec). G_o_ has the dimensions (kg^2/3^/m^2^) for y = 2/3. m has the dimensions necessary to make the expression for the mass of the non-skeletal musculature in equation (1) have the dimension of mass. G_r_ has the dimensions (watts/m^2^). MMLE avoids the physics challenge of the multiplicative constant a in relationships like aW^b^ when b varies among different phylogenetic groups as was found in (White, Blackburn & Seymour, 2009; Capellini, Venditti & Barton, 2010; Isaac & Carbone, 2010; Hudson, Isaac &, Reuman, 2013).

It is a signature feature of MMLE theory that the vertebrate body is represented as a combination of masses instead of a single mass. There are at least two masses: 1) the skeletal musculature which is governed by dynamic similarity and in which the power producing organelles are approximately uniformly distributed; and 2) the non-skeletal musculature in which the power producing organelles are concentrated in surfaces that surround material with few power producing organelles. This binary nature of the vertebrate body can lead to different exponents in the relationship BMR = aW^b^ for different phylogenetic groups within the same infraclass. As cited in (Hulbert & Else, 2004) Hemmingsen observed in 1960 that a body composed with this sort of binary distribution of power producing organelles could account for values of the exponent b that are different from the geometric similarity value of 2/3.

In MMLE theory equations (1) and (2) are exact once numerical values for the parameters have been established. G_m_, G_o_ and G_r_ are universal constants that should apply to all vertebrates. y and m should have the same value for all animals in a phylogenetic group . k is a constant whose value should be similar for all vertebrates that are dynamically similar. The fundamental propulsion frequency, f = c/l^r^, should be the same function of the characteristic length, l, for all vertebrates that are dynamically similar. e is a constant whose value should be approximately identical for all vertebrates in the same phylogenetic group with the same body temperature. The characteristic length, l, and the sturdiness factor, s, have unique values for each individual animal.

MMLE thusly uses 10 parameters to compute the absolute values of the body mass and BMR of a vertebrate. 10 is only one more than the number of factors that McNab (McNab, 2008) found influenced mammal BMR; and it is less than the over 22 parameters needed to best describe the overall mass and temperature dependence of BMR by the (White, Frappell & Chown, 2012) information-theoretic analysis. While that analysis did include insects, spiders, protists and prokaryotes as well as vertebrates, it and McNab’s findings do indicate that needing a large number of parameters to compute the absolute value of a vertebrate’s body mass and BMR should not be surprising. Even describing the relationships between BMR and body mass, W, and a skeletal dimension, l, for a collection of animals with the simple relationships BMR = aW^b^ and W = dl^x^ requires at least five parameters and as many as six if BMR and length data are not for the same individual animals. Simplicity is not necessarily better than complexity (White, Frappell & Chown, 2012).

In the original paper G_m_/k, G_o_ and G_r_ were considered nearly invariant among vertebrates so that there were only five degrees of freedom for equations (1) and (2).

When the gravitational force dominates the dynamics of animals’ movement, two animals are dynamically similar when the ratio of gravitational force to inertial force is the same at corresponding stages of their motions. The animals are Froude similar and they have equal Froude numbers, F, where F = u^2^/gl and u is speed, l is the characteristic length and g is the acceleration of gravity (Alexander, 2005). Running/walking mammals are Froude similar (Alexander et al, 1979; Alexander, 2005; Raichlen, Pontzer & Shapiro, 2013).

Strouhal similarity obtains when inertial forces are proportional to oscillatory forces. Similarity implies equal Strouhal numbers, St, where St = fl/u and f is the frequency, l is the characteristic length and u is speed. The Strouhal number governs a series of vortex growth and shedding regimes for foils undergoing pitching and heaving motions thereby describing the tail or wing kinematics of swimming or flying animals (Taylor, Nudds & Thomas, 2003).

Reynolds similarity obtains when inertial forces are proportional to viscous forces. Similarity implies equal Reynolds numbers, R, where R = ulρ/ν and u is speed, l is characteristic length, ρ is fluid density and ν is fluid viscosity (Alexander, 2005). Bats are the only animals examined in the present paper for which viscous drag, and hence Reynolds similarity, might be important. As will be seen when bats are addressed Strouhal similarity does apply to bats. If both Reynolds and Strouhal similarity simultaneously apply, then by solving for speed, u, in the definitions of Reynolds and Strouhal numbers, the frequency, f, is seen to be proportional to the inverse of the characteristic length squared , or f = R ν/St ρl^2^. This sort of dependence of the frequency on the characteristic length was not observed. It should be noted, however, that the characteristic length for viscous drag and that for vortex growth and shedding could be different body dimensions.

Two animals are geometrically similar if one can be made identical to the other by multiplying all its linear dimensions by the same factor (Alexander, 2005). Properties of geometric similarity include surface area, S, being proportional to the square of the characteristic length, l^2^, and simultaneously volume, V, being proportional to the cube of the characteristic length, l^3^. Since mass, W, is proportional to volume, mass is also proportional to l^3^. From equation (3) geometric similarity of the skeletal musculature means that the fundamental propulsion frequency exponent r = 1.0. The fundamental frequency constant, c, in equation (3) has the dimension of speed. If the non-skeletal musculature is also geometrically similar with y = 2/3, then the entire animal will be geometrically similar.

Froude and Strouhal dynamic similarity are separately compatible with geometric similarity.

If both Froude and Strouhal similarity simultaneously apply then equating speed in the definitions of the Froude and Strouhal numbers results with the frequency, f, being proportional to the pendulum frequency, (g/l)^0.5^. Substituting this expression for f in equation (1) shows that mass, W, is not proportional to l^3^ and thus geometric similarity does not apply.

**______________________________________________________________________________**

**
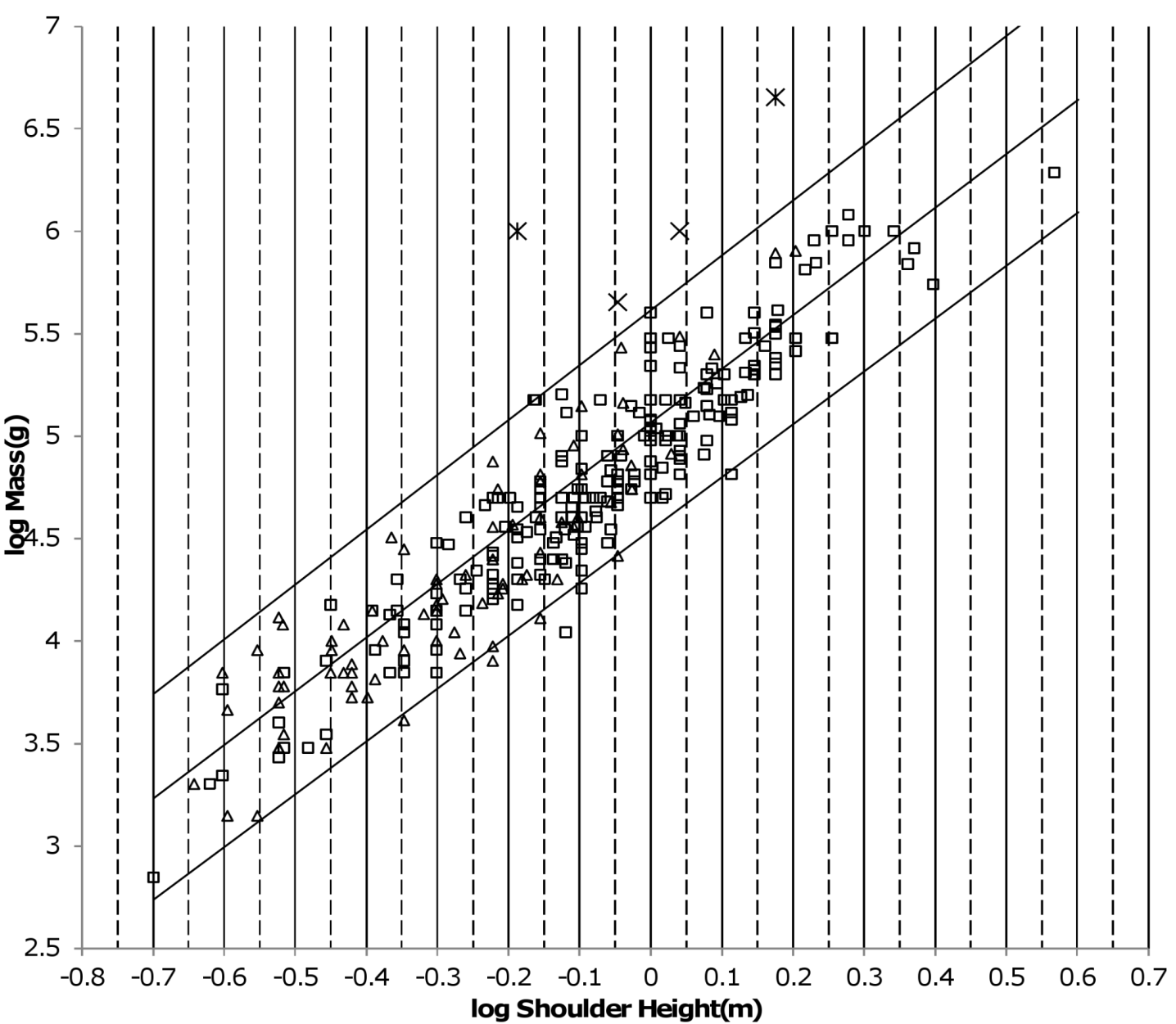
**

**Figure 1. Log body mass as a function of log shoulder height for running/walking Artiodactyla and Carnivora.** Data are from (Nowak, 1999). The upper and lower slanted solid lines are MMLE sturdiness factor boundaries for y = 2/3. The upper boundary was generated with a sturdiness factor, s, of the square root of 3, (3)^0.5^. The lower boundary was generated with s = (3)^-0.5^. The middle slanted line was generated with s = 1.0. The slanted lines are for Froude-Strouhal dynamic similarity. The Artiodactyla mass and shoulder height data are marked by open squares. The Carnivora mass and shoulder height data are marked by open triangles. Excluding *Hippopatamus amphibus* marked by crossed Xes and domestic cattle marked by Xes, R_M_^2^ = 0.9997. The solid vertical lines demark the AVG method first set of cohorts. The dashed vertical lines demark the second set of cohorts.

If only one or the other of the dynamic similarities apply without geometric similarity, there is no particular constraint on the fundamental frequency of propulsion, f. Consequently there is no particular constraint on the exponent r in equation (3).

From equations (2) and (3) geometric similarity means that BMR is proportional to body mass raised to the 2/3 power, W^2/3^. Simultaneous Froude and Strouhal similarity implies that BMR is proportional to body mass raised to a power greater than 2/3. If only one or the other of the dynamic similarities apply without geometric similarity, BMR could be proportional to body mass raised to a range of powers.

Hereafter, when it is stated that geometric similarity applies it also means that either Froude or Strouhal dynamic similarity also applies.

Examining body mass and BMR from the skeletal length perspective rather than from the body mass perspective lead to the concept of the sturdiness factor. The sturdiness factor is a most important concept of MMLE theory.

The sturdiness factor is best understood by looking at Fig.1. Figure1 plots 314 samples of log body mass versus log shoulder height for running/walking mammals from the orders Artiocactyla and Carnivora obtained from (Nowak, 1999). Shoulder height is a good surrogate for characteristic length for running/walking mammals as will be discussed in a subsequent section.

For each mass and length datum in Fig. 1, a sturdiness factor value can be found so that the mass is exactly computed by equation (3) using the found sturdiness factor value and the datum length. Also for each BMR, body mass datum from a data set such as (Kolokotrones et al, 2010) a sturdiness factor value and a characteristic length value can be found so that the datum BMR is exactly computed by equation (2) and the datum body mass is exactly computed by equation (3) using the found sturdiness factor value and the found characteristic length value.

Figure 1 only plots 314 samples. If every individual Artiodactyl and Carnivore in existence were plotted in Fig. 1 then the data, due to its density, would appear as a blackened area. It was found in the original paper that most of the blackened area would be bounded by an upper line computed using equation (3) with the sturdiness factor set to the square root of 3, (3)^0.5^, and a lower line computed with the sturdiness factor set to (3)^-0.5^. The blackened area would thusly appear to be a black band bounded by these two lines. Most data would have a sturdiness factor with a value between (3)^0.5^ and (3)^-0.5^. These boundaries are plotted as the upper and lower slanting lines in Fig.1. Excluding *Hippopatamus amphibus* and domestic cattle, over 97% of the data plotted in Fig.1 are contained between these boundary lines.

The data bordering the upper line are for sturdy animals such as a large American black bear (*Ursus americanus*) with W = 270 kg, l = 0.91 m and s = 1.63 or a large water chevrotain (*Hyemoschus aquaticus*) with W = 15 kg, l = 0.355 m and s = 1.35. The data bordering the lower line ~~is~~ are for gracile animals such as a small bob cat (*Felis rufus*) with W = 4.1 kg, l = 0.45 m and s = 0.556 or a large roe deer (*Capreolus pygargus)* with W = 50 kg, l = 1.0 m and s = 0.687. At the same characteristic length an animal with a greater sturdiness factor is more massive than an animal with a lesser sturdiness factor - hence the nomenclature ‘sturdiness’ factor.
